# Distribution and Diversity of Cytochrome P450 Monooxygenases in the Fungal Class *Tremellomycetes*

**DOI:** 10.3390/ijms20122889

**Published:** 2019-06-13

**Authors:** Olufunmilayo Olukemi Akapo, Tiara Padayachee, Wanping Chen, Abidemi Paul Kappo, Jae-Hyuk Yu, David R. Nelson, Khajamohiddin Syed

**Affiliations:** 1 Department of Biochemistry and Microbiology, Faculty of Science and Agriculture, University of Zululand, KwaDlangezwa 3886, South Africa; akapoolufunmilayo@gmail.com (O.O.A.); teez07padayachee@gmail.com (T.P.); KappoA@unizulu.ac.za (A.P.K.); 2 College of Food Science and Technology, Huazhong Agricultural University, Wuhan 430070, China; chenwanping@mail.hzau.edu.cn; 3 Department of Bacteriology, University of Wisconsin-Madison, 3155 MSB, 1550 Linden Drive, Madison, WI 53706, USA; jyu1@wisc.edu; 4 Department of Systems Biotechnology, Konkuk University, Seoul 05029, Korea; 5 Department of Microbiology, Immunology and Biochemistry, University of Tennessee Health Science Center, Memphis, TN 38163, USA

**Keywords:** *cryptococcus*, *cryptococcus neoformans*, cytochrome P450 monooxygenase, CYP51, fungal pathogens, genome data-mining, human pathogens, CYP diversity analysis, *tremellomycetes*, *trichosporon*

## Abstract

*Tremellomycetes*, a fungal class in the subphylum *Agaricomycotina*, contain well-known opportunistic and emerging human pathogens. The azole drug fluconazole, used in the treatment of diseases caused by some species of *Tremellomycetes*, inhibits cytochrome P450 monooxygenase CYP51, an enzyme that converts lanosterol into an essential component of the fungal cell membrane ergosterol. Studies indicate that mutations and over-expression of CYP51 in species of *Tremellomycetes* are one of the reasons for fluconazole resistance. Moreover, the novel drug, VT-1129, that is in the pipeline is reported to exert its effect by binding and inhibiting CYP51. Despite the importance of CYPs, the CYP repertoire in species of *Tremellomycetes* has not been reported to date. This study intends to address this research gap. Comprehensive genome-wide CYP analysis revealed the presence of 203 CYPs (excluding 16 pseudo-CYPs) in 23 species of *Tremellomycetes* that can be grouped into 38 CYP families and 72 CYP subfamilies. Twenty-three CYP families are new and three CYP families (CYP5139, CYP51 and CYP61) were conserved across 23 species of *Tremellomycetes*. Pathogenic cryptococcal species have 50% fewer CYP genes than non-pathogenic species. The results of this study will serve as reference for future annotation and characterization of CYPs in species of *Tremellomycetes*.

## 1. Introduction

Cryptococcosis is a fungal infectious disease ubiquitously distributed around the world [1]. Two fungal species, *Cryptococcus neoformans* and *C. gattii*, are the main infectious agents causing cryptococcal meningitis in both immunocompetent and immunocompromised humans [1,2,3,4]. This disease is the major cause of morbidity and mortality among people living with advanced HIV and annually accounts for 15% of all HIV-related deaths globally [5,6]. The burden of HIV-associated cryptococcal disease in Sub-Saharan Africa is alarming, as 73% of deaths in the world are reported in this region [5,6]. Apart from these opportunistic pathogens, the genus *Cryptococcus* contains species with biotechnological potential (Table 1). Among the cryptococcal species, *C. amylolentus* is closely related to the pathogenic *C. neoformans* and is extensively used for comparative studies to identify the pathogenic traits in *C. neoformans* [7].

The genus *Cryptococcus* belongs to *Tremellomycetes*, a fungal class in the subphylum *Agaricomycotina*, which contains organisms adapted to different niches and/or having different lifestyles (Table 1). Some of the organisms in this class are now regarded as emerging opportunistic human pathogens and some species are adapted to extreme ecological niches, such as cold regions (Table 1). Despite being fungi, *Naematella encephala* and *Tremella mesenterica* Fries exhibit fungal parasitism. The diverse lifestyles or characteristics of some species of *Tremellomycetes* are summarized in Table 1.

In countering cryptococcosis, three classes of antifungal agents are available: polyenes (such as amphotericin B), azoles (such as fluconazole) and the pyrimidine analogue to flucytosine [1]. The gold standard induction treatment includes giving amphotericin B along with flucytosine [23]. However, this combination therapy has substantial side effects and the need for intravenous medications poses a problem, as these are not readily available in developing countries, which are most affected by cryptococcosis [24]. To overcome this problem, a combination of fluconazole along with flucytosine has been recommended after initial therapy with amphotericin B and flucytosine [1,23].

Fluconazole binds to the fungal cytochrome P450 monooxygenase (CYP/P450) enzyme 14α-demethylase, named CYP51, which converts lanosterol into ergosterol, an essential component of the fungal cell membrane [25]. *C. neoformans* also has *CYP51* and quite a number of studies have indicated that the development of drug resistance to fluconazole is due to the mutations in the *CYP51* gene and to the elevated levels of CYP51 in cryptococcal species [26,27,28,29,30]. In addition to *C. neoformans*, drug resistance in other species of *Tremellomycetes* has also been reported owing to mutations in *CYP51* [31,32]. Recent studies have demonstrated that the new anti-cryptococcosis drug named VT-1129 that is in the pipeline strongly binds and inhibits CYP51 of *C. neoformans* and *C. gattii* [33,34,35].

Despite the importance of CYPs as drug targets, to date, the CYP repertoire in cryptococcal species or in other species of *Tremellomycetes* has not been elucidated. A few studies reported the CYP contingent of *C. neoformans* and *T. mesenterica* Fries with the purpose of comparing the CYP profiles with wood-degrading fungi [22,36,37]. Thus, in this study we present a comparative analysis of CYPs in species of *Tremellomycetes*.

## 2. Results and Discussion

### 2.1. Pathogenic Cryptococcal Species Have Few CYPs in Their Genomes

**Figure 1:**
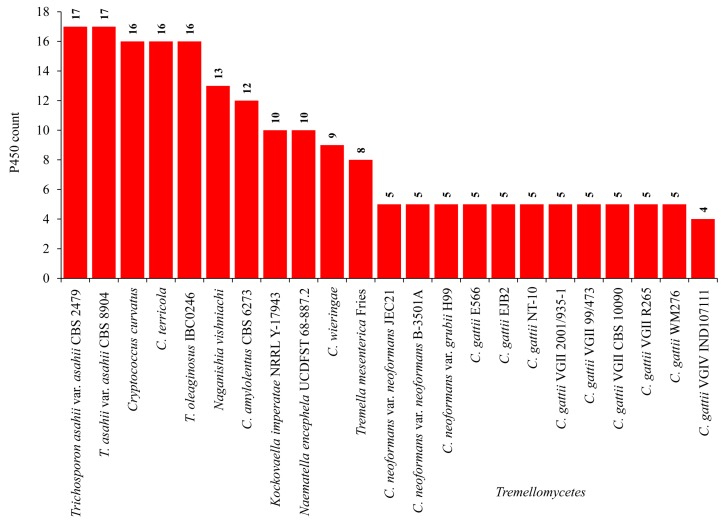
Comparative analysis of CYPs in the species of *Tremellomycetes*.

Genome-wide data mining of CYPs in 16 cryptococcal species revealed the presence of 112 CYPs in their genomes (Figure 1). *C. curvatus* and *C. terricola* have the highest number of CYPs (16 CYPs each), and *C. gattii* VGIV IND107 has the lowest number of CYPs (Figure 1). An interesting pattern was observed when comparing the CYP count among cryptococcal species. Almost 50% fewer CYPs were found in pathogenic cryptococcal species compared to non-pathogenic cryptococcal species (Figure 1). This suggests that adaptation to survive in a host (mainly animals) that has a rich source of simple nutrients might have led to the loss of CYPs. The same phenomenon was observed in fungal species belonging to the subphylum *Saccharomycotina*, where species lost a considerable number of CYPs owing to their adaptation to simpler carbon sources [38].

The comparison of cryptococcal species’ CYP count with other species belonging to the same subphylum *Agaricomycotina*, especially the well-studied wood-degrading fungi, is not logical, since the wood-degrading species have quite a large number of CYPs in their genomes [22]. As cryptococcal species fall under *Tremellomycetes*, in this study, a comprehensive comparative analysis of CYPs in *Tremellomycetes* was carried out (Figure 1). As shown in Figure 1, the comparison of CYPs among species of *Tremellomycetes* indicated that pathogenic cryptococcal species have a lower number of CYPs compared to other species of *Tremellomycetes*. Fungal parasites such as *T. mesenterica* Fries and *N. encephela* have eight and 10 CYPs in their genomes, somewhat lower than non-pathogens. It is interesting to note that the species belonging to the genus *Trichosporon* have the highest number of CYPs in their genomes, both pathogenic and non-pathogenic (Figure 1). It is well-known that most of the species belonging to this genus are considered commensals of the human skin and gastrointestinal tract, and these species are now increasingly causing superficial and invasive diseases in immunocompromised individuals and intensive care unit patients [18,39]. This indicates that these organisms have a long way to go to adapt better, similar to the cryptococcal species, and thus, in this process they may lose CYPs as well.

### 2.2. New CYP Families Were Found in Tremellomycetes

**Figure 2:**
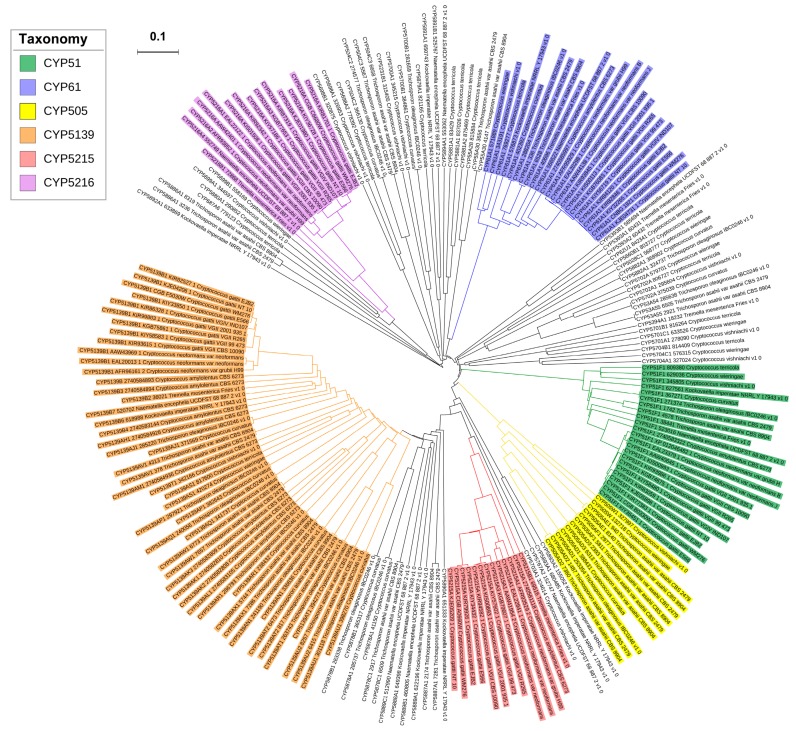
Phylogenetic analysis of CYPs from the species of *Tremellomycetes*. CYP families that are highly populated in species of *Tremellomycetes* are indicated in different colors. A high-resolution phylogenetic tree is provided in Supplementary Figure S1.

A total of 203 CYPs were found in 23 species of *Tremellomycetes* (Figure 2 and Supplementary Dataset 1). Sixteen CYPs were found to be pseudo/false positives, as they lack one of the CYP characteristic motifs and/or short fragments (listed in Supplementary Dataset 1). Thus, these CYPs were not included in the study. The annotation of CYPs as per International P450 Nomenclature Committee rules [40,41,42] in combination with phylogenetic analysis (Figure 2) revealed that 203 *Tremellomycetes* CYPs could be grouped into 38 CYP families and 72 CYP subfamilies (Figure 2 and Supplementary Dataset 2, sheet 1). Phylogenetic analysis of CYPs is critical in assigning the CYP family and subfamily for the CYPs that have a borderline percentage identity of around 40–41% (for a family) and 55–56% (for a subfamily) with the named fungal CYPs.

A total of 23 new CYP families, named CYP5215, CYP5216, CYP5393, CYP5394, CYP5698-CYP5702, CYP5878-CYP5882, and CYP5886-CYP5894A1, were identified in species of *Tremellomycetes. Kockovaella imperatae* NRRL Y-17943 have the highest number of new CYP families (six CYP families: CYP5888-CYP5893) followed by *Naganishia vishniacii* (five new CYP families: CYP5698-CYP5702), *C. terricola* (three new CYP families: CYP5879-CYP5881), *T. mesenterica* Fries (two new CYP families: CYP5393 and CYP5394), *T. oleaginosus* IBC0246 (CYP5878 and CYP5882) and two new CYP families (CYP5886 and CYP5887) were found in *T. asahii* var. *asahii* CBS 2479 and *T. asahii* var. *asahii* CBS 8904. Three species of *Tremellomycetes* have only one new CYP family: *C. neoformans* var. *grubii* H99 (CYP5215), *C. neoformans* var. *neoformans* B-3501A (CYP5216), and *N. encephela* UCDFST 68-887.2 (CYP5894).

### 2.3. Four CYP Families Are Conserved in Pathogenic Cryptococcal Species

**Figure 3:**
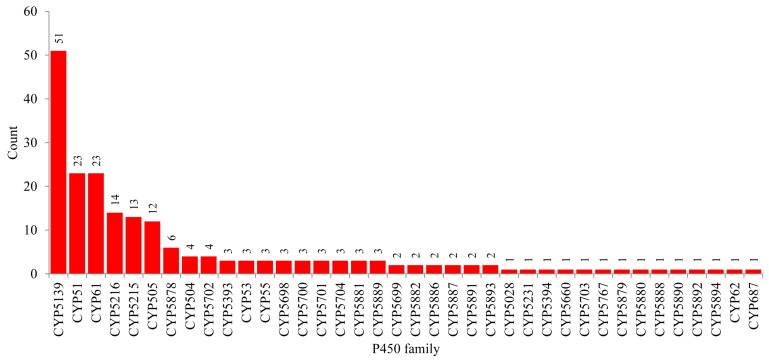
The CYP family-level comparative analysis in the species of *Tremellomycetes*. The numbers next to the family bar indicate the total number of CYPs. The data on the number of CYPs in each CYP family, along with subfamilies, are presented in Supplementary Dataset 2, sheet 1.

**Figure 4:**
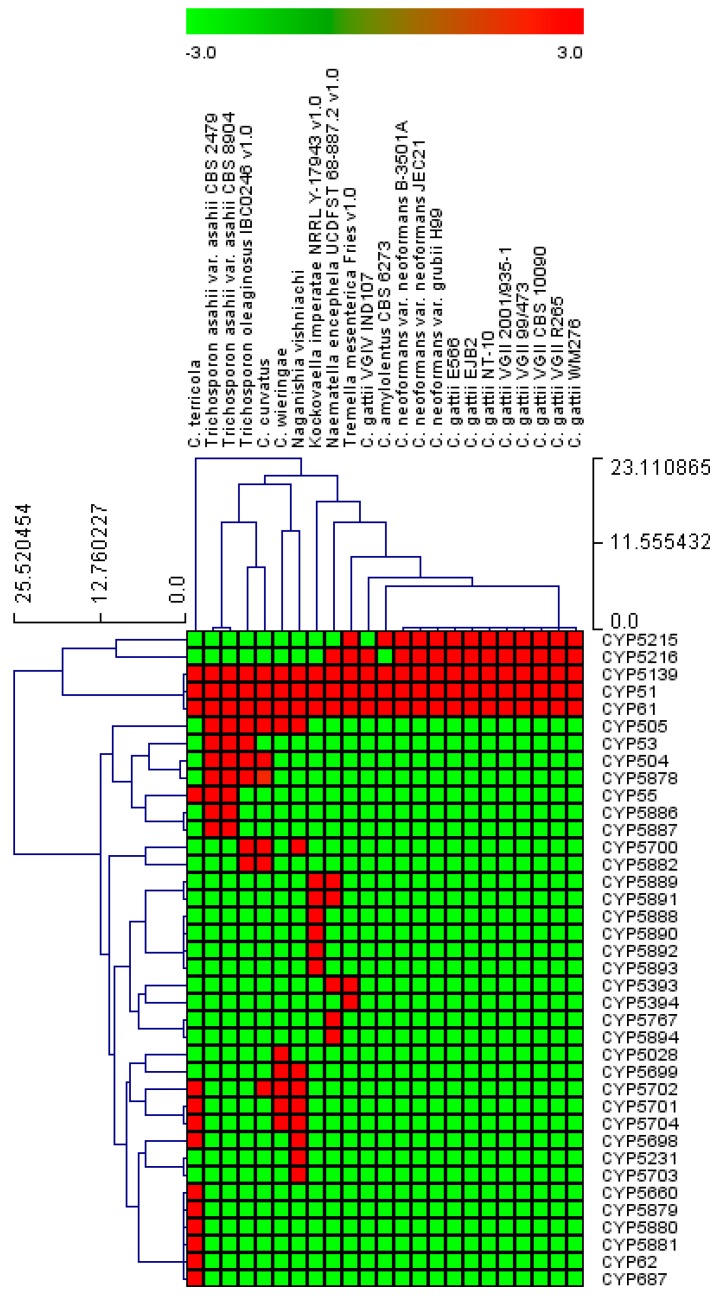
Heat map representing the presence or absence of cytochrome P450 families in 23 species of *Tremellomycetes*. The data have been represented as −3 for gene absence (green) and 3 for gene presence (red). Twenty-three species of *Tremellomycetes* form the horizontal axis and CYP families form the vertical axis. The data used in the generation of Figure 4 are presented in Supplementary Dataset 2, sheet 2.

CYP family-level comparative analysis revealed that among 38 CYP families, the CYP5139 family was found to be dominant in species of *Tremellomycetes* with 51 members, following the CYP51 and CYP61 families each with 23 members, the CYP5216 family with 14 members, the CYP5215 family with 13 members, and the CYP505 family with 12 members (Figure 3). Analysis of CYP family conservation revealed that three CYP families, namely CYP5139, CYP51, and CYP61, are conserved in all 23 species of *Tremellomycetes* (Figure 4). CYP family comparison among pathogenic cryptococcal species revealed conservation of two more CYP families, CYP5215 and CY5216, in all species except *C. gattii* VGIV IND107, which does not have CYP5215 (Figure 4). These two CYP families are also present in fungal parasites, *T. mesenterica* Fries (both families) and *N. encephela* UCDFST 68-887.2 (only CYP5216 family), and non-pathogenic *C. amylolentus* CBS 6273 (Figure 4). The CYP family CYP5231 found in *N. vishniacii* is also present in the fungal species, *Melampsora laricis-populina* and *Puccinia graminis*, belonging to the class *Pucciniomycotina*, where this family is bloomed in both species [36]. The presence of the CYP5126 family only in pathogenic or parasitic *Tremellomycetes* indicates that this CYP family might be playing a role in the adaptation of these organisms to their host. The analysis of CYP subfamilies revealed that the CYP5139 family has 17 CYP subfamilies, indicating the blooming of members in this family. The same was observed for quite a number of CYP families in other fungi [36,37].

### 2.4. Pathogenic Cryptococcal Species Have the Highest CYP Diversity

**Figure 5:**
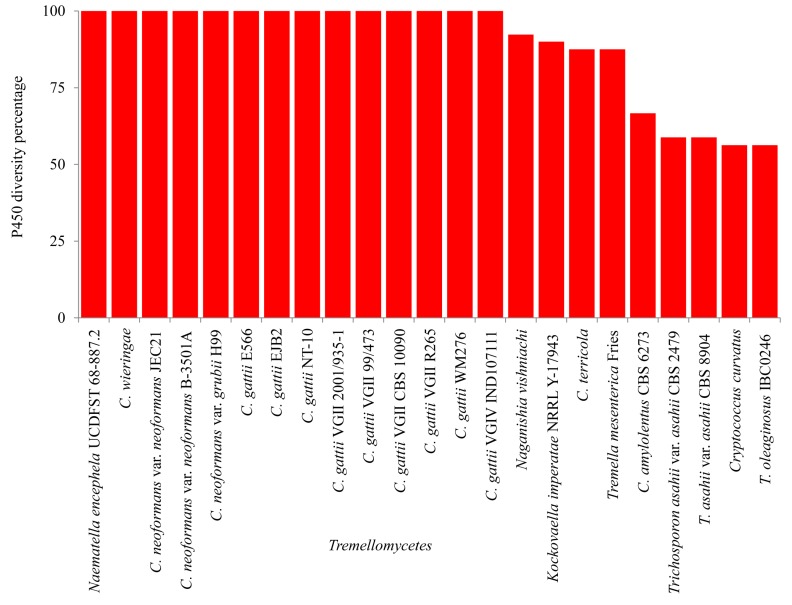
CYP diversity percentage analysis in *Tremellomycetes*.

CYP diversity analysis revealed that pathogenic cryptococcal species, along with non-pathogenic *C. wieringae* and the fungal parasite *N. encephela* UCDFST 68-887.2, have 100% CYP diversity in their genomes (Figure 5 and Supplementary Dataset 2, sheet 3). *Tremellomycetes* such as *C. curvatus*, *C. amylolentus* CBS 6273 and *T. asahii* var. *asahii* strains had the lowest CYP diversity percentage. This is due to the blooming of CYP5139 members in their genome (Supplementary Dataset 2, sheet 1). The highest CYP diversity observed in pathogenic cryptococcal species is perfectly matched with species belonging to the fungal subphylum *Saccharomycotina* [38]. One commonality can be found between the species belonging to *Tremellomycetes* and *Saccharomycotina*: It can be assumed that some species of *Tremellomycetes* lost CYPs, compared to their counterparts, which may be due to the adaptation to use simple carbon sources present in the host, as observed for species of *Saccharomycotina*, where the loss of CYPs in response to the adaptation to use simpler carbon sources was observed [38].

### 2.5. Most of the CYPs from the Species of Tremellomycetes Are Orphans with No Known Function

Among CYPs from the species of *Tremellomycetes*, CYP51F1 of *C. neoformans* has been shown to be involved in 14α-demethylation of lanosterol [30] and the *CYP51F1* gene was cloned from *T. asahii* ATCC MYA-1296 = OMU239 = TIMM4014 [27]. Apart from CYP51F1, some of the CYPs’ functions can be predicted based on characterized homolog CYPs. CYP61 family members are involved in membrane ergosterol biosynthesis where they catalyze C-22 sterol desaturase activity [43]. CYP505 family members are involved in oxidation of fatty acids [44]. CYP504 family members are involved in conversion of phenyl acetate to 2-hydroxyphenylacetate [45]. CYP53 family members are involved in detoxification of toxic molecules, including benzoate and its derived compounds [46,47,48]. The primary function of CYP53 is the conversion of benzoate to *para*-hydroxy-benzoate. A study reported that CYP53 could be an alternative antifungal drug target in view of its critical role in fungal organisms [49]. It is interesting to note that this CYP family is only present in three species belonging to the genus *Trichosporon* (Figure 4 and Supplementary Dataset 2, sheet 1). CYP55 family members are involved in the reduction of nitric oxide (NO) to nitrous oxide (N_2_O) [50,51]. It is interesting to note that in addition to CYP53, CYP55 family members are also found in two species belonging to the genus *Trichosporon* (Figure 4 and Supplementary Dataset 2, sheet 1). Apart from the CYP families listed above, the rest of the CYPs found in species of *Tremellomycetes* are orphans.

## 3. Materials and Methods

### 3.1. Species and Databases

Twenty-three species of *Tremellomycetes* were used in the study (Table 2). Different databases, such as NCBI (https://www.ncbi.nlm.nih.gov/), FungiDB [52] and Joint Genome Institute MycoCosm portal [8], were browsed for cryptococcal species genomes. All the species’ genomes used in the study have been published and are available for public use, with the exception of two species, *C. wieringae* and *N. vishniacii*, and permission to use these two species’ CYPs was obtained from the principal investigators listed at the respective species’ databases at the Joint Genome Institute MycoCosm portal [8].

### 3.2. CYP Mining and Annotation

Genome mining for CYPs and subsequent annotation was carried out following the protocol described elsewhere [36,38,53,54]. Briefly, proteomes cryptococcal species from different genome databases, listed in Section 3.1, were downloaded and subjected to an NCBI conserved domain search [55] to classify the proteins into different subfamilies. The proteins grouped under a CYP superfamily were selected and subjected to blast analysis against fungal CYPs [42] to identify homolog CYPs. Based on percentage identity with a named homolog CYP, the hit proteins were then assigned to different CYP families and CYP subfamilies, following the International P450 Nomenclature Committee rule, that is, >40% identity for a family and >55% identity for a subfamily [40,41]. CYPs that had less than 40% identity with named fungal CYPs [42] were assigned to a new family. For each species, CYPs from different databases were compared and duplicate CYPs were removed from the final CYP count.

### 3.3. Phylogenetic Analysis of CYPs

Phylogenetic analysis of CYPs was carried out following the procedure described elsewhere [36,53,56,57]. Briefly, first, the protein sequences were aligned by MAFFT v6.864 [58], and embedded on the T-REX web server [59]. Then, the alignments were automatically subjected to tree inferring and optimization by the T-REX web server. Finally, the best-inferred trees were visualized and colored by iTOL (http://itol.embl.de/about.cgi) [60].

### 3.4. Generation of CYP Profile Heat-Maps

The presence or absence of CYPs in species of *Tremellomycetes* was shown with heat-maps generated using CYP family data following the method described elsewhere [54,61]. The data were represented as −3 for gene absence (green) and 3 for gene presence (red). A tab-delimited file was imported into multi-experiment viewer (MeV) [62]. Hierarchical clustering using a Euclidean distance metric was used to cluster the data. Twenty-three species of *Tremellomycetes* form the horizontal axis and 38 CYP families form the vertical axis.

### 3.5. CYP Diversity Percentage Analysis

CYP diversity percentage analysis was carried out as described elsewhere [53,57,63,64]. Briefly, the CYP diversity percentage in species of *Tremellomycetes* was measured as a percentage contribution of the number of CYP families in the total number of CYPs.

### 3.6. Functional Prediction of CYPs

Literature was searched for characterized CYPs from species of *Tremellomycetes*, if any. Furthermore, functional prediction of CYPs was carried out based on the characterized homolog CYPs from different fungal organisms. CYP family level functional prediction was presented in the article.

## 4. Conclusions

Infections caused by human pathogenic species of *Tremellomycetes* are regarded as neglected diseases. Research on unraveling the infectious fungal pathogens’ physiology and development of novel drugs against these pathogens is seldom done because of the lack of a lucrative market. However, cryptococcal meningitis remains a huge killer among people living with HIV in Sub-Saharan Africa and some of the species in the genus *Trichosporon* are now emerging human pathogens. This study’s results provide insight into the CYP enzymes in the species of *Tremellomycetes*. This study revealed that cryptococcal species have almost 50% fewer CYP genes than their non-pathogenic counterparts and furthermore have the highest CYP diversity. Four CYP families were found to be conserved in pathogenic *Cryptococcus* species, indicating their important role in these pathogens. Interestingly, the CYP5139 family was bloomed with 17 CYP subfamilies in species of *Tremellomycetes*, indicating its possible key role in the physiology of these organisms. This study serves as a reference for future annotation of CYPs and has opened new vistas for the characterization of CYPs in the species of *Tremellomycetes*.

## Figures and Tables

**Table 1:** Some species of *Tremellomycetes* and their well-known characteristics.

Species Name	Information	References
*Cryptococcus neoformans*	*C. neoformans* causes meningitis in immunocompromised and apparently in immunocompetent humans. This organism is considered a major opportunistic pathogen and a leading cause of mortality in patients infected with HIV.	[2]
*Cryptococcus gattii*	*C. gattii* causes respiratory (pneumonia) and neurological (meningoencephalitis) diseases in humans and animals and it can infect immunocompetent hosts.	[3,4]
*Cryptococcus terricola* JCM 24523	*C. terricola* is oleaginous yeast and has been suggested as a candidate for the consolidated bioprocessing of hydrocarbon chemicals. It has the ability to accumulate unsaturated 18 carbon chain length fatty acids, with additional minor contributions of saturated 18 carbon and 16 carbon fatty acids.	[8,9,10]
*Cryptococcus curvatus*	*C. curvatus* is oleaginous yeast capable of accumulating 18 carbon chain length fatty acids while growing on low or negative cost feedstock. Thus, it is a potential candidate for the use in industrial fermentation processes. In a rare case *C. curvatus* was found to be involved in peritonitis associated with gastric lymphoma.	[8,11,12]
*Naganishia vishniacii* (formerly known as *Cryptococcus vishniacii*)	*N. vishniacii* is psychrophilic yeast adapted to live in extreme conditions, such as low-temperature oligotrophic deserts. It also has the ability to grow in a low-nutrient environment, without added vitamins.	[8,13,14]
*Cryptococcus wieringae*	This is associated with pectin hydrolysis during the dew-wetting process of flax and found at the beginning of grape wine fermentation.	[8,15]
*Cryptococcus amylolentus* CBS 6273	*C. amylolentus* is the most closely known related species of the pathogenic *Cryptococcus* species complex, and is non-pathogenic.	[7,16]
*Kockovaella imperatae* NRRL Y-17943	*K. imperatae* is a non-pathogenic fungus used in the analysis of widespread adenine N6-methylation of active genes in fungal species.	[17]
*Naematella encephela* UCDFST	It is a parasite of another fungus, *Stereum sanguinolentum*. This fungus’ genome sequencing was carried out for the analysis of widespread adenine N6-methylation of active genes in fungal species.	[17]
*Trichosporon asahii*	Some species belonging to the genus *Trichosporon* are considered emerging opportunistic human pathogens and are the third most commonly isolated non-*Candida* yeasts from humans. They live in soil and are adapted to colonize the skin, gastrointestinal, respiratory and urinary tracts of humans. *T. asahii* is the most important species causing disseminated disease in immunocompromised patients, while the inhalation of *T. asahii* spores is the most important cause of summer-type hypersensitivity pneumonitis in healthy individuals. Some *Trichosporon* species have also emerged as rare but frequently fatal pathogens causing disseminated infections (trichosporonosis) in immunocompromised individuals and intensive care unit patients.	[18,19,20]
*Trichosporon oleaginosus* IBC0246	*T. oleaginosus* is oleaginous yeast with the ability to accumulate lipids equivalent to biosynthetic kerosene, and thus is a biotechnologically valuable player for the generation of environmentally friendly (carbon-neutral) energy by converting agro-industrial waste to fuel (biodiesel).	[8,21]
*Tremella mesenterica* Fries	It is a parasite of crust fungus of the genus *Peniophora* and has a false appearance, as if it were growing on wood. Whereas in fact, it grows on the crust of fungal mycelium.	[22]

**Table 2:** Species of *Tremellomycetes* used in the study. The respective genome databases used for analysis of CYPs, along with the reference articles, are listed in the table. Abbreviations: NCBI, National Center for Biotechnology Information and JGI, Joint Genome Institute.

Species Name	Database	Reference
*Cryptococcus gattii* VGII R265	NCBI	[3,4]
*Cryptococcus gattii* NT-10		
*Cryptococcus gattii* VGII 99/473		
*Cryptococcus gattii* E566		
*Cryptococcus gattii* VGII 2001/935-1		
*Cryptococcus gattii* VGIV IND107		
*Cryptococcus gattii* VGII CBS 10090		
*Cryptococcus gattii* VGII 2001/935-1		
*Cryptococcus gattii* EJB2		
*Cryptococcus gattii* WM276	NCBI	[3]
*Cryptococcus terricola* JCM 24523 v1.0	JGI	[9]
*Cryptococcus curvatus* ATCC 20509	JGI	[11]
*Naganishia vishniacii* v1.0 (formerly known as *Cryptococcus vishniacii*)	JGI	
*Cryptococcus wieringae*	JGI	
*Cryptococcus neoformans* var. *neoformans* B-3501A	NCBI	[2]
*Cryptococcus neoformans* var. *neoformans* JEC21	NCBI	[2]
*Cryptococcus amylolentus* CBS 6273	JGI	[7,16]
*Kockovaella imperatae* NRRL Y-17943 v1.0	JGI	[17]
*Naematella encephela* UCDFST 68-887.2 v1.0	JGI	[17]
*Trichosporon asahii* var. *asahii* CBS 2479	JGI	[19]
*Trichosporon asahii* var. *asahii* CBS 8904	JGI	[20]
*Trichosporon oleaginosus* IBC0246 v1.0	JGI	[21]
*Tremella mesenterica* Fries v1.0	JGI	[22]
*Cryptococcus neoformans* var. *grubii* H99	NCBI	[2]

## Supplementary Materials

Supplementary materials can be found at https://www.mdpi.com/1422-0067/20/12/2889/s1.
