# Supplementary material for: Distribution and Diversity of Cytochrome P450 Monooxygenases in the Fungal Class Tremellomycetes
Source: Int J Mol Sci. 2019 Jun 13;20(12):2889. doi: 10.3390/ijms20122889 (PMC6627453; doi:10.3390/ijms20122889)
Supplement: Supplementary file 1 [file ijms-20-02889-s001.zip › Supplementary Information/Supplementary Dataset 1.docx]

Article

Distribution and diversity of cytochrome P450 monooxygenases in the fungal class *Tremellomycetes*

Olufunmilayo Olukemi Akapo ^1^, Tiara Padayachee ^1^, Wanping Chen ^2^, Abidemi Paul Kappo ^1^, Jae-Hyuk Yu ^3,4^ David R Nelson ^5^*, Khajamohiddin Syed ^1,^*

^1^ Department of Biochemistry and Microbiology, Faculty of Science and Agriculture, University of Zululand, KwaDlangezwa 3886, South Africa; akapoolufunmilayo@gmail.com (O.O.A); teez07padayachee@gmail.com (T.P.); KappoA@unizulu.ac.za (A.P.K.); khajamohiddinsyed@gmail.com (K.S.)

^2^ College of Food Science and Technology, Huazhong Agricultural University, Wuhan, Hubei Province, China; chenwanping@mail.hzau.edu.cn

^3^ Department of Bacteriology, University of Wisconsin-Madison, 3155 MSB, 1550 Linden Drive, Madison, WI 53706, USA; jyu1@wisc.edu

^4^ Department of Systems Biotechnology, Konkuk University, Seoul, 05029, Republic of Korea

^5^ Department of Microbiology, Immunology and Biochemistry, University of Tennessee Health Science Center, Memphis, TN, 38163; drnelson1@gmail.com

***** Correspondence: drnelson1@gmail.com (D.R.N.) and khajamohiddinsyed@gmail.com (K.S.)

**Supplementary Data Set 1:** CYP sequences identified in the species of *Tremellomycetes*. CYPs were presented with their given name, protein ID in parenthesis and species name. Proteins IDs represented from different databases listed in Table 2. List of pseudo-CYPs or false positive CYPs identified in the species of *Tremellomycetes* are presented at the end of CYP sequences in a table format. In the table, if given the CYP name for pseudo/false CYPs that is also listed.

>CYP5881A1(837026)Cryptococcus terricola

MSLLSQHTPPSLFSYLTDPYIFLPILIVSIYPLYQALLIILTPRPIPGIPHRPTSVLFGD

GLLMGKHLKEKGTPTVFFDDLVLELGDQGDGGICQAVFGFFKRNKVVVVSDRQEAEDILT

KRSHEDTINTFSPFIPNAQLSLRTNTRWKHHRRLMGPAMTSKNLSMFTPRAVSVFSRLVE

LWKFKAKRLGEGEWFNCDEDLQRTFMDAICEITFGESYRCLESAIEYLQSLPDDEIVDHS

EDGTVIFDSEPPAIFTATQVLFWAIPTTELFPKLAMWRTSLTTTFRRASNLVNSFLDRKI

VEGRDRANARGLDSVELADCVLDLVCARETGKDRLDDPSMRDELMQFLLAGQETSATTTC

WMISYLSKRTKLKKELVELGLSDRPPTFADMQADKAPYLEAVVHEVLRCGNTAGGVTRDT

IQDTTILGHFIPKSTLIYIPLATIQSFPGRQAKIDENRSETSKTSDKSILPWDPETISDF

EPERWLKDGIFDARAGPWMPFSMGSRGCYGRALAMLELKLFLAAINLNFFLEPIPEGKQP

EKIVEHLTRKPTVTYVKPRPW*

>CYP62U1(842341)Cryptococcus terricola

MSDFTSIRKSLVFSEYLERAARIFHAEPALLLSYLIAGYFAFKLLQILHTGFVGGKLRSV

PGPFLTRFTNWPLRWATIRGKQIFFIHELHCKYGPYVRICPNQVSISDLQSVKEIHKIGT

GFLKTDFYQRLTHHESDCLTSFAMRDPKQHIQRRKLLASPMSKNGLSQWEDLIHEKLQMA

IDGIKRSATVDGKANILAWFTFMTHDVISTLAFGDSFKSLEAGAVSLLITKTQYILDLEA

TLKHDSISIELPWLAPILDFLGPRTRLHAAGSEAYERTVDLEKKGYHRGTVFSKMFLEEG

QPPTVKQIKAEAVLFLVAGSDTTAVSLTYLIFEVLSNPAIRERVLEEVNSLPDDFTTHDA

LNLRYLSRVRQEVLRLHTAAPAGLPRTVPKGGRMMGGYYIDDCVIVSTQAYTLHRDPAAF

PDPLKFDPDRWLEPTQAMKDAFMPYGAGSRVCLGIHLATMEIMLTAVMFLRALPNVKLAQ

ETTCKAMEPENFFLIQPMAHACYVEL*

>CYP687A6(779123)Cryptococcus terricola

MFSSDVIADDFDIFASQVPADIYNTYDSLRQGCPLAYTSQYNGFWLLTRHEDIKKVAMDS

DTFISSNKAVIPSDPRGLRRPPLNFDAPHHTPYKKALNRSLKPTRIAKLRPTLMKHAEKE

LEPLLIAGGGDVSGTFGVRFPAWVEVEWLNLDSLIAPVLADTAAAWITAWRDGNGPEVGK

QSEKLYGIARDLLADRKVNPRDPDEDPASSLLLERDSHGQPLEEEYLIGALRQSLVVGMV

APPLIISSICVHLANDKNLQNLLRSDRSLIPSATEEFLRLYVPYRGFARTSSRDVEMHGR

LIRPGEPIAIAYSSANRDPAVWDDPNVFKFDRENITQHLGFGRGRHSCAGMPLARMMIQI

SLEVLLENTTDIEVDGELAYAKMPEMGVVKCPVRLIQ*

>CYP5660B1(803727)Cryptococcus terricola

MILDRLPMGVFVGAQSALVLTVLSGVAAYAVYIAYNLLLHPLARVPGPFLARSGALPFMA

IHCFRQDVAWTIEALHLQYGPTVRIGRNRIATIDPIAVHKVYGHGSRFNKSRFYDFFSIT

GQPSAFTSRDIHEHAVIRRTISNAFTMSSLIELEEYVDEVMVVLLRYLDGAAAAGKPIDL

GKVSQYFAMDVVGELGFGQGFNLLRDGKDQYNMLSSLDVGLWLSSILGSIPTIGHPIFKL

LLLLPVPGARYLDRTANVCVDERMRRANAAGKDGGDARKDMLTKLVKARNPNTKQPLSRE

EIITHASTIIGAGSDTTSITMRAFFAFVLARPRVLARLREEIDEAVEYGRVVLPASYAQG

VQLEYFQACLKETLRLWPAVAHPMERVVPAGEVTQFNQFVIPPGTVVGTSSLAFHRSYEA

FGEDAAQFRPERWLAVDETERKRLEKNNIAFGAGSRVCIGKNISLMELTKVIPQLIYRYD

MTFTARTVGSPHKHAIGRGPDGKTGEDVPYYVESNWFTNPKDFWCDISRRSREEEQ*

>CYP5879A1(821165)Cryptococcus terricola

MPTTQMSLDTVLYLLLSGLALVAAFTLKDAWKHRKNRWKSDSPLPPPHVQSWIPWLGSAL

ALAANPDKFLRKAQKKYGPLFSLKAAGKSMIYATSEATITYIYRNPKEFEFTSIRREMQI

AVFGLSPARDSDWMDNHLFPLNKRWSKLLDHSILSPAGIAPLLKSFATFTHEDMHNKWHS

LGSEEELVDVSKLVYDLIFSASSKAFFGPTFPSVGGFDGFLDFDAGFSLLAGGLPKFMCK

KAIESRSKALDVLEEWYQSILDEEAKGKNLLNDPEFPFFQMPAACGEEHFSRRDIAVILL

GGAWALLANAPVAAIWCVIKLIEHPDAMARVRSEIEIGLRESADGKIETLIQTLIINSDH

FGFLGSVIQETLRWTSSSYSIRRVTADTVIPYPVGSPGSLVHKGDMVVCVTSMNHSDPTI

YSNPGEWIAERFTTEFKKGRADRGEKDVTNGFLPFGGGVSMCEGRHFASSELRIAIICFL

YYFDVEKTTREPAQLDYSRLGLGILPVKGNCMLRLKKRSIA*

>CYP5881A1(83429)Cryptococcus terricola

MSSFLPPASYYDQLVSHLPIPWYTTTHPLLLLVVIVVLGCWSTYRIIRYLLTPKPFGFIP

HLSDQRMIVGDAGGLAKFIKKMNSHTSWFEGVVEKLGRPPLCEVVLGLGDHTIIVNDIQE

AEDILIRRSHEFDRSNLTLSLFSPVVPSAQIAIKTDAMWKHHRRVMGPTMTSSSIKRFTP

RASKTVRKMIQLWKLKYDKGLFDPKQDLEFATTDAICEITFGESLGCLDSAIETVKQPDK

VIQDSTGFVTYDYHPPAVFASMQWLFETISIRSIFPRIEHFFREMTPTYKQHTGLINKWI

YRKMDEGRQAFKARGKEAHELVDCVMDVICLKDEGADALTDPEIRDELIQYVIAGQDTGA

TSLLWAMKYFGRNPHVQRKLHKEILEMQLDDDPDYHDLAKMHYLEAVTWEVLRLAAAGPS

TNRKALVDVTVLGHVFPKGTVFYFPLRMFGNQPAPDHGSIVEKRRSPSSLEAGKREGKWD

PATIADFNPDRWLDSEGLFDPKAGPALPFGLGQRGCFGRSLAVVEMKLFLYHIIGNFFFE

PVPLELDGFEETENVTRKPAQCWIKICPWADSEL*

>CYP5881A2(875469)Cryptococcus terricola

MSNFLRQLTGAAPADHSTLLHYLRDPYILIPLLLVSAVPIYQALISIATPKPLKGIPHLP

KQAPIVGDAVEIGKHIKKFHSVTGWFDTVVERLGNEGDGGICQVIVGFGSSGKMVVVSDA

QEAEDILTKRTSEFDRSSDTIAVFSLILPNGQISMPTDSKWKHHRRLVGPSMTSKNLNMF

TPRAVDTISKMIELWQVKSERLGEDGYFDSVEDFARATMDAICEITFGESYQCLEMALDT

VKKMPKQAIERELDGSVDFPCEVPAIYTATQVISSSLPIVAVSPKLTFFFTSLTSKFRSS

HKILNGFLKRKIREGRARAVERGQDKAESVDCVLDLICLKESGSDRLDDESMRDELHQFL

IAGQETTATTLTWTMIYLAKNPDVQRKLHKELVELGLGERVPGFYDVQADKAPYLEAVVY

ESLRLAVTAGGVTRRAMTDTTILGHFIPKDTIIYVPLSTTQCRSGYQCKDDSLRSESSKG

IKPWDAEDISKFIPERWIVDGEFDGRAGPWMPFSAGARGCMGKPLALLELKLFVALINIA

FFLGPIPADKQPMEFTETVTRKPIVSMVRPVPW*

>CYP5882A1(368902)Cryptococcus curvatus

MEALKTFTAQLTPATVSAQSNILAGATALGVLAHLYWQRFEPDPISYGQFALALGGGLVY

FFYHFLGSLPTAILYTAEVFTVFNTVVLLSMVIYRLSPWHPLAKYPGPAAAKVSKIYWWY

QAKIGQTGYTQAKLHDQYGDFVRIGPNWISVRSADAIPIIYGGSKLGAGHGRAPWMKNDW

YEGAIRGATRISVHQEVDVDKHHIRRKLWDHGFTGKALADYKPEILDFCDKLVRNVNMVV

ARGNATGKPQAIDMGLQSSYFSFDVMGVLGFGEPFGMLDAGVKHLFVDTLEKAMRTLIPF

HEISWIRILLRKLLPLPERLKVFEKQNEERFERREKLGSARRDLFTYLLGEEKDGAETFK

LTRLELIADAGTIVVAGSDTTSSLLTWFWYYSTKHPETYKTLRDELAGLNADQLIDTTLL

SKLPYLNAALNETMRMQPAVPGGLRRLVPMGGVEIEGHYLPEGTVLSVPGYAIQHDPRFW

GKDCNEYRPERWLDTSGKYNKNAYLPFSYGSRGCPGKGLAMVEARLAVAQLATRFEFAFP

PGFSQREFEEGVRDHFTIQNMPLPLVPIPSPVAKAA*

>CYP5139AT1(394273)Cryptococcus curvatus

MPSLPLVSGAIDWPFLLAVGGGAAALWLAYSLFHLLVPEARLSSSFKNLPGPPNPRNWTR

ILGHQYEALTRGPALTSEEWHDKYGPTIRLSRPFGRSELSTTDPAALTFIYRATDERFVK

SEGMRQAISANVGEGVLSVEGHTHRRHRRVLNPAFGWPQIVEMVPSMWLKAYQLRDKMVS

NITDAWASPPADPRDVVPGARVLNMFNELSNAALDIIGLVAIGHDLGSLSDEPNELRDGY

NDVLRVGFQTDIWTVLRFALPITRKIPLERTRIVRAGRDAVVRFGQRVIDEKRRLLLNID

QGRIEKGTDVGKDVLSLVIKANAAADLRDDERLNDTEVIAQIATMLFTGHETTGTATAFT

LRHLSLNQRFQDKLRAELLTIDVDEPDFQVLDKLPYLHAVAREGLRFETPVPIMGRIATA

DVAIPLSIPVRGRDGKMIDVVQLKKGDLINAPYQAANRLTDVWGADAREFNPERWFRPNM

PAKKMPGVWGEIATFGGGPHNCIGHRMAVAEMKVLLFVLIRNFIFSPAPSGPVIKPKWMI

IQRAIVEGEEHKGPSMPLLVRPVAE*

>CYP5139AN1(336836)Cryptococcus curvatus

MVFRDHFGAWRNFPGPRPTHWFWGNIHEVMQTSPQQCHQRWLDKYGRVVRVHDVGGRAKL

VCSDPVATQYLLLHPDAFIKPAGPNRILTDALDKGLVTVEGRAHRRQRRVLNPAFGWVQI

QGLAPMIMDKVGEMRDKFARLIEEEMAGTGRVGRRMDVLHYMSETALDIIGEAGFGYDFN

AIGDANSPLRNAYTSAVQHAFEFDATVMLTVMDARFGAIPTSRVRNMHAARSAAQVIGRE

IVAEKKKQVINYNKGLDKESLGKDVLSLMIRANMDPALRPDQRLTDDEVVAQIATVLFAG

HETTSTALMWCLYHLSLHQDMQQRLRDELLEVPDERPDFDTLMNLPYLDKVVHESLRLES

PVPNISRVATQDGVVPLSAPVTGRDGRPIEAIRVRKGEVIEVPLTYMNTLAEVWGPDARE

FNPDRRDTGVPTQQLPGVWGGIMSFIGGPRNCIGHRLTILEMKAVLFALLRSFVFEQLPD

PPKIKRTYMVIQRAVVVGEEEYGPQMPLLVRPYVET*

>CYP5139AQ1(341737)Cryptococcus curvatus

MLAGLADAPRWALYSVAATILFTAFMIYRYPYRERVLHYRNLPGPPSSHFLFGDFPKVQS

HPTGRVMQAWFDEYGPTVRAKLLLGDNLIVTSDTTAMAFIMQHADHFIKPPAQTRMITRL

LGHGLLNVNFDTHRRQRRVLNPAFHPDAIRDMVPHMFTKAYELRDKLMEACADPEYYDPE

TAAPRPEDVVPGARKVNMNKYLINTTFDVIGLAGFDYEFGCLRTGNHIVTTFRNAMSELQ

RLTLFGVLQQMIPALDVIPSKRNKLADQARIGTSGVGRECIARKRREMEAMHQDDLNKGT

FVGKDLLSLMLKANMAKDLLPRDRLDDTEMAYQVSTFILAGSETTSNSLTWTLYRLAMNP

DVQQRLREELDTLASDEPSLEQLNTLPYLENVIHESLRLDPPVPESMRMVTQDVMLPLHT

PVKGRDGTMIDSVPMKKGDMIITGYMQVNHNKEIWGADADRFNPDRFDRPGIPARKAPGT

WGNITSFNGGNRNCIGYRFALAEIKAILFVLLRHFQFEMLPSSPTITAKNFIIMLPWVVG

EENVGSQMPLMVRPIVE*

>CYP504C2(366130)Cryptococcus curvatus

MSSKLSAFDLNNVQLGFNSQTLAIVGVALLAISVVQYVRYARGQIHLPGPTPLPIFGNLL

QLDDDAAQTYYKWSKKYGAVYKVTLGEREVVVVNTAEAAKYLFGDLGNIYISRPLFHNFH

NVVSSSAGFTIGSSPWDDSCKRKRRAAATALNKTAVQSYVPIIDRETLALIEDIYRDCGK

GTKEINPYSYLQRLALNVSLVVNYGARLDDISDAMFHEIIEVETTVANFRSLSNDMADYV

PLLRYLPTSKSTVDVAEDARKRRDKYMKKMLDDLKARVANGTDIPCIMGNILKDPEAQLT

ELELSSICLSMVSAGLDTLANTFIWSVGYLAKHPEVQEKAYKAINDVYHGAIPDSTEEVV

EYITALHKECSRYFSVLKLALPRATIGDSEWRGVHIPDGTTVFLNAWAIHHDAERYGDFE

TFRPERFLDKSEANQQAHYSFGAGRRMCAGVHLANRELYVAFCKLIYFFKLELGSEDFDI

NPATACSNPRGLSSQPHPFKIRFVPRDADTIEEWIADEKNRAELKLAASMTNKQ*

>CYP5139AP1(382643)Cryptococcus curvatus

MEGVVELARAHPILTLLSLATLFPAFLILWAYPYREYTLPYRNLPGPKPSSVFWGNYSEI

LKEPTNAPQTRWIADFGNAFRYRTLFGKPRICLADPVAIAHIAQHTYDYHKAGPTVYALE

LVLGRGLLTVEGDDHRRQRKIMSPAFGLPAIKSMHPIFMEKAWELQRKLSAILDTEEETY

CSTPVKPEDRVLGTKKVDMIPYWGQMTLDIIGLAGFDYDFGALAGRSTDLADSFRDLLRA

GNSPGILAILAAVIPPLRALPNKLTNAVGAAKAQTDRVAREIVQQKKVLVAAELATGEKD

ETLGRDLLSRVVRANMDPALRPEQRLTDEEVIAQVVTFIIAGHETTATALTWMVFRLAEN

WHVQKRLRDELRAFPHDNPSFDELNALPYLDKVVHEGLRLDPPVAGGVRVAAKHYIIPLG

QPIRGRDGKMISSIDVPVGTDIFIRVYGNLLTFFNGARNCIGYRLTLAEMKAALFCIFRH

LEVLPLPSNPEIRLKAQIVMRPLVVGEEEAGYQMPVLVRAIPDDD*

>CYP5139AU1(398270)Cryptococcus curvatus

MSTVLAQPLPLTLTPQVVGYVLLSLVVVAPLALWFYLYPYTAATLPFKNLRGPEPTSGFF

GSLITVSTKPIGQRYASLTEQYGASTVRFRGLVGRWRIMSTDTTAIAHVLRHTGQWHRNE

GFNGLIERMTGANVLCVEDEPHRRQRRILNSAFNGTSVSAMMPMFWEEAYDLKKNVEELL

VEGQEKGTQVDMLQVYVRTAMDIIGRAGFNYYFEQHKVNGENPLSKAFNDMINGMLENRK

LVLVQNIFPQALDLPTANHRRVAKSRAVLDGIARDIVQSRRAEIARDHISLDKGDYEGKD

VISLCLRANMGASERDRMTEAEIEGQIGALMLAGNETSATALSWATLHLFENPDIQAKLR

AEVMAVPDDEPSVEVLNGLPYLDGFVRELLRLDPPLPQVVRTAAVDCVVPLGTPVQGRDG

SLISEIRVSKGTDFVVPISEMNRDPRIWGADADKFNPDRYDGTVPRVSLPGVWGGLLSFI

GGPHHCLGFRFALLEIKAVLFVVLRNFAFEPVPGKQFIRFSAGIIQRSIVIGEESKGMQM

PVIVRALESE*

>CYP5139AR1(238432)Cryptococcus curvatus

MTESLIASGAARAVAAITSLSLLQVTGYLVLGVLVGLVGTWAYLYPYAAYFSSSRNLPGP

PSEHPIAGNLLYVLRRQHPRPVYQAWIDKYGPTGRIRGLLGVERIYTSDPAVISHIFQHA

DMWPKSKSTNIMLRRMLGNGLITAESSDHRRQRRVLNPAFSPRAVHEMAPIFFEKARAVR

EKFAQLLEESATTRDRVVLVDTDAMQATDNKGGKIDVGLLMEQATLDTIGAAGFDYDFAT

LFSRRSELLDAFHSAVKALQASALISALQNRFVLFNALPTPGLKLGRASRATMNRVGDRI

VAEKTRAITNMGIEKDTDLATADLLSRLIRANMAVDLEERARLSHAEVQAQICTFIFAGS

ETTGVALTWALYRLATDKAMQDRLRAELVTISENPDLDEIHSLPFLEKFTREILRMDSPV

PAVGREPAAKTTLPLSRPVRGRDGKMMDSVVVDKGTEVVILVGNVNRDPAIWGPDSNEFN

PDRYDRPPLASVPGVYGNLLTFIGGPRNCIGWRLALAEFKIILFTLLRAYELAEVPGTTL

HSETTLGIMRTRSNEEGVVVPLLVRPVGE*

>CYP5139AJ1(371569)Cryptococcus curvatus

MSTMSSLTDIPFVAKLADIPVVSTLAAHPYITAVLLTLVLGYIYCFPYSDYRYGIHNVPG

PPNPHLFWGNLQEVIKAPPNAMHEQWFNTYGNVIRYKFLMGAQRLTSVDMGFIGYVLQHN

DEFIKPVRAQEMLERLLGNGVLIAEHAAHRRQRRVLNPAFSLAAIRDMTPIFYDKSYELQ

RKLAELIEEPSELDQAAPTPAKPEDVVKGARKIDVMRYMGAATLDVIGLAGFDYDFNALR

DNKNELAEAFRTMFSAGTSPSAMNILQNFIPILQFVPTKAREATRRSRAVTVRIGKELMD

NKKAAVRALFGEVEKGSDIGKDLLSLVIKANMASDLRPDQQLTDDEVLAQITTFMLAGNE

TSSTALTWLLYRLALHKDVQDRLRAECQAVPTDRPSMDEINALPFLDQCVHESLRFDNPV

ASTIREAVSDQVIPLSEPVMGRDGKMMSEIHVKKGTTIFLPIMNVNRSKEIWGEDADKFN

PDRFSVGGIPRKHVPGVFGNLLTFLGGARNCIGYRFALIEIKMILFVLMRSFEFDELPSK

PEYEAKSSIVMRPRVVGEEDAGLQMPLLVRLLEQ*

>CYP505AD1(388431)Cryptococcus curvatus

MPPCNPLTTTMPSKEVTRPAGKSGEKGRPIPQPAPKLIVGNIFDIDPLKSMQSMMRLARI

HGEIYQLKLFEMIIFVSSQELVHYISDETKFEKCVSKPLQEVRNFAGDGLFTAFNSEHNW

TLAHRILIPAFGPLAIKKMQPMMLDVITQMLMFWEHHAGEPLEAADQFTRLTFDTIGYCA

FKYRFNSFHTEKVHPFIDIMVKLLAESQARSRRPGLMQSVMWSSEAEFRANVKELHRLCD

EIVAARRRNPDPNAHDLLNNMILDSDPKTGEHLSDENIRYQMVTFLIAGHETTSGMLSFA

TYYMLKNPSTFAKAREEADAAIKKAGGSLLKVNPADLKYIDAICKEALRLQPTAPAWAVV

PKSEKGETLPGGYYIAPGQNVVALLLMLHRDPKAWGEDAEAFRPQRWLDGRVIPPDAWKP

FGNGQRGCIGRGFAMQEAVLAIALIVARFDLEMADPTYDLEIKETLTLKPENFKIIARPR

FGRNQSILSELLASANGSNNYTYTTEAKGDVATSSGGHGEKIYVLYGSNSGSCEGLSKEL

VSEGDARGFNMRIGELDSVAGGGVLPTDAPVIIVTASYEGQPTDNARMFVAALAANKNTD

SLKGVRYMVMGAGHHDWAQTFHKIPKFIDTRLAELGAERLVPLSTGNAAEDIVGDFEAFK

ARVWKHFGGGPASTDAAAMPADESAQPFLLLPRTANPAAATVGCGIETVGTVIEQDVLTR

VSENSLQTNQITIQLPKGQQYRAGDYLAVFPKNPKPTVDRALEHFGLHTEDTVVFNRPTS

FFPLKLPMRVGDMLSSFVELGQPVSKTVLPQLLPHCDEQATKRLEALEANYVQEVVGPRL

SLLDLVLTTPGCKPPMDVFLANLPKMKIRQYSISSTPLEEPDKVTLTFTVHTAPSAVGDR

VLGVASNYLAFLNRGEDLLCTVKGSAGFHLPADPEVPVVMFAAGSGIAPFLGFIAERAQM

AAGGRKVGRTVLYYGTRTSVEMVRKDKFAQWVKDGVLDFRPVLSRSNAKEVAGVPGVALL

PEAKYVQDRVWVDKDDMADLFAAGAQYYTCGSGQRLGHDLKQTLVKIIAEKKPEGDNETP

EAIMERFARERYRTDVFL*

>CYP5028C1(568777)Cryptococcus werringae

MLDSVILSTLEPYIGLKTLAVVLALSLPIWIAYHLFFSPLSSIPGPLVARMGLSIIKPRS

AVSLTWVWDLERLHEQYGPIVRVSCNHLSVSDPDALPIIYRIANPLHKTNFYHTFMAIPG

KPSLFSDTDFESHKTRKKAVGPAFAMTFLTNLEESVENVTETLTGRLRDKIEQDGGSTAV

EFDKLAHYFAMDAVGEIAFGRGFDLLSPDSFSSEETIIQNDKFLRGVSNLSQWGGMAGGL

PPWMGRRALDLFKRWTSQPGSEVIADVTRTRVAARYNEVLNGKEARKDMLNKFMNAKHPD

GTPYSQNDVLSTATSIVAAGADTTAVALGSLLASLTSHPAVYAKLQDEIDEAFCDGLLCK

PVAYGKAAQLPYLQACIKESLRIHPPISMEIPRVVPSEGMILCDRFIPGGTSVGISPYIV

HRQKAAYGDDADTFRPERWIEAELAEKEEGDGGQARRRLEKYYFVFGGSTTSCIGKNISL

MEISKVLPELLHDFAFATIPRGPRSPHALPPRHRDGNRYEPSGNPAPWYCSSSWFLETQD

LWLRVSERNPEQV*

>CYP5139B4(2740583144)Cryptococcus amylolentus CBS 6273

MAFSAGASIQWLQPSTSGMSYTQSFAIVLTSIFFVLLGTYLLLWPIAEWRLPASFRNLPG

PRDDSWIAGVLPSLLTEPVNAPHTRWTNTYGLTLRYRIFFGIPRLLTIDPTVLSYVLYHP

DLFPKCNATKRMLVDMVGKGLLVVEGEEHRKQRRALSACFTPNALKGMTSIFYDKAYELK

DKLANMIEGVDPEICAPTPTKAADEVEGGKQIDVMRYLAKTTLDVIGLAGFSYDFNSLSE

SRNELADAFGSMFAAGAEIGFVGFLQIVFPVLKWIPTARSKAIARAKEVTQRIGLRIIQD

KKGEIMATHEKDLEKNVDLGKDLLSILIKANMAADLKPEDRLTDQEVLDQITTFMFAGNE

TTSTAVTWCLYQFAKKPEVQERLREEVLAVADERPSLDTLHSLKYMDIVVREVLRLYAPV

PTGIRKAGQDIVIPLGTPVIGRDQQIITSVEVSKGTAIFVPTGSINTSPLLWGPDATEFR

PERFSQDLSTGSTGGWGQIPGVWGNVMTFLGGPHNCIGYRFSLAEIKVLLFVLLRNFEFQ

ELHSKPEIEKKASVVMRPKVVGEEEAGPQLPLMVRPISAL

>CYP5139B3(2740584894)Cryptococcus amylolentus CBS 6273

MDTLASPAYPFATMPRFFILPSVLTVLYSLVAVSLLSAGLYIYLYPYRLFRLSFRNLPGP

EPGHWFWGHTQTLIAQQPNAMHTVWTNKHGPTIRYPIFLGIQRFLTIDPSALNYILTHPE

SFEKPSETRGELTTMLGNGLLVAEGSDHKKQRKALGPSFSPAAIRGMIPAFYDKAYELNN

KLMGLVEGADTEVASPTPAKDIDFVPGGKKIDVMSYLSKTALDIIGTCGFAYDFKALSEP

SNELSDAYRNMFMAVMDVRVWDFIVSKIPIIRSFPTKRSKVVAASKKLTRSIGMSLLREK

KDIVMGAHGGDLEKKADIGKDLISILVKANMAAGLKPEQRLSDEEVLDQITTFMLAGNET

TSTGLTWILYNLSQNQDVQEKLRQEVQSVSEDRPSLDTLNTLPYMEAVVHESLRISPPAS

FTLREATQDAILPLANPVTGNDGKVMHEVAIDKGTSIFIPVITVNTSPSIWGDDAAKFIP

ERWLSSSKDGFQPGPVPGVWGNTLTFLGGGRNCIGYRFALAEIKVILFVLMRSLMFEELK

SKPVISKKTGIVMRCSIEGEEEAGLQMPLMVRPVAA

>CYP5139AH1(2740584928)Cryptococcus amylolentus CBS 6273

MGLHYPLELTASSSWTSCLLRFLLYGMAGFTVILLVAYLYIFPLQERSLAYRNLPGPKSP

SIFWGSIKTVLAHSHPNAAYDIWLAAHGPTLRYNILLGMSRVLTIDTTAISYILSHPDLF

PKPVHGRAILGDMLGKGWCFSPNSIKGMAPIFFDKAYELKRRLLGLIDDDAESQASPTPP

LEQDKQPNGRKIDMLKWTGKATLDVIGLAGFDYDFQTLEGGENELAEAYREMFEIGMGMS

AMSVIQGLFPALRFIPTERTQVLNRSRSITRRVGMEIIQRKKQVVQTYYAGFLEKNVDIG

NDLLSILIKANMAHDILPEDRLSDDEVLDQITTFMLAGNETSSTALTWVLYTLSQHPTVQ

HRLRAELQSVPDDHPPIETLLALPYMDAVVKEVLRLSAPIPMALREAAQDCVLPLATPVV

GRDGRVLDSVALNRGTTVLMPNLNVNTSEDIWGPTASQFIPDRFLDPIPELYPATTKAVP

GVYHNLMTFLGGSRNCIGYRFALAEMKVMLFVLMRGFEFEELNSRPEIERKVSVVMRPRV

VGEEEAGLQLPLFVKPLSDLC

>CYP5139AM1(2740584936)Cryptococcus amylolentus CBS 6273

MGHLLSLPTSLLFAVASRFLAAALLLVAAAYLYFWPIRRYFSHHRNLPGPYSSSVFWGAL

PYVYSFPHPTTAYQNWLTTYGPTFQYPILLGNSRIITADLAAISYILSHPAIFPKPVHVR

AALREMIGDGLVSEEGDVHKRTRKNLNPSLGPAAVRKMVPIFYDKSRELAERLSSDLDDQ

RDNRINFLWYISRATLDMMGVAQFDHDFHALSGEAEEIVDAYTGMFQVGTEVSVMTVLQF

LLPVFKRIPTNRTAVLDRSLRTARRFGRKIIREKRDNLRSFDLEKDQDHDIGLLASMREV

QSLEDITDKVTVKNQATSCPDERLTDEEILDQVNTFMLAGYETTSTALCWCIYSLAKHPE

IQERLRQELLDISEQQPDMQVVASGSWALLMVTSDTLNALPYLDAVIKESLRLSAPVPLA

LREASQDISIPLSTPITGKDGNLMDRVEVGKGTTILMPISNANTQLSIWGPRAEEFLPSR

WLTPTPETYPGSAKSVPGVYSNLLTFWGGSRSCIGFRFALAEIKVMLFVLIKSFRFEELE

NRPLIEKKFSIVSHPWVVEEENAGFQLPLKVSGVQMT

>CYP61A(CGB_F1500C)Cryptococcus gattii WM276

MESHTILRPTAIPDLAAIKTWGLEGLTKAKFSFDSKTTTATVLTLILSLLVLEQLVYRAK

KAHLPGAKWTIPVIGKFADSLNPTLANYKAQWNSGPLSAVSVFNIFIVIGSSNEMARKIL

NSPNHAEPCLVASAKKVLLPENWVFLHGKVHADYRKALNVLFTKQALSIYLPIQERIYRS

YFNKWMSDPAPAQQYMMKMRDLNMETSLSVFIGPYLTEAQKQEINEKYWLITISLELVNF

PLAIPGTKVYNAIQARKIVMKYLSAASAASKIRMEDDDAEPECLLDHWVRAMILARRAQD

DGEETKLLSREYSDHEIAMVLLSFLFASQDAMSSALVYTFQLTADHPEVLAKVREEQYRV

RGNDLERPLTLDLLDDMVYTRATIKEVLRFRPPVIMVPYMTTKPFPVSPEYTAPKNSMII

PAFWNSLHDETCYPEPDRFLPERWLPQADGSAPIADSKPQNYLVWGSGPHKCIGGQYASM

HLAATLGTASVLMDWKHERTELSDEVQVIAAIFPKDHCLLKFTPRAPPS

>CYP61A(343191) Naganishia vishniacii v1.0

MQNASQALAGALPTANADNPSLLINLQQSLQLSSLPTTYLNLFQPTTTTNTAAASPSWLS

KSVLGSLLALVATLLIVEQVVYRSKKAHLPGARWTIPVIGKFADSMNPKLDNYKKQWASG

ALSVVSVFNIFIVIASSNEYSRKILNSPNYAEPCLVASAKPILLKENWVFLHGKVHADYR

KALNVLFTKKALSIYLPIQERVYRKYFKSWINDPCPTAKPYMMAMRDLNMETSLRVFLGD

YISDQGAQEISDKYWLITLALQLVNFPLAIPGTNVWNAIKARKIAMVHLTAAAGASKRRM

AQGGEPDCLLDAWISEMISARAGDGDEDSKRVLSREYSDHEIAMVVLSFLFASQDAMTSA

IVYLFQLMADHPEILAKVREEQYRIRGADVEGSTTLDMVDDMVYTRACIKETLRLMPPVI

MVPYLAKKAFKISEDYTAPAGTMVIPSFWNSLHDEHVYPKPDEFIPERWMPLPEGGVPLA

EQSPQNYMVWGSGPHKCIGVQYASMHLAAVIGSASVLMDWEHDKTPKSDEAQVLCTLFPK

DGLPLKFRPRAPPS*

>CYP61A(769277)Cryptococcus terricola

VFLHGKVHADYRKALNVLFTKKALSIYLPIQEAIYHKFFNSWMSKTNSSPEPYMMQMRDL

NMETSLRVFCGDYISEAGAQEISDKYWLITVALELVNFPLAIPGTKVYNAIQARKIAMKH

LEAASAASKIRMASGAEPSCLLDAWVSEMIQARAGNGEDEQQRRVLSREYSDHEIAMVVL

SFLFASQDAMTSAIVFLFQYMANHPDVLAKVREEQYRVRNNDVMAPLTLEVVDEMVYTRA

CVKELLRLLPPVIMVPYLTKKDFPIAPDYTVPKGSMIIPSFWNSLHDEHVYPEPDKFLPE

RWMPLPEGGIPLAEQSPQNYMVWGSGPHKCIGVQYASMHLAAVIGSASVLMNWEHERTKE

SDQVQVIATIFPKDGLRLKFTPRPAPA*

>CYP61A(571889)Cryptococcus werringae

MAGIISQPASLASSTSNPLSLLPLPSVVADSLAKFGIFQFAGNGKQALGRLLFGFGKEGW

VTSLLVLEQLVYRAKKAHLPGAKWTIPIIGKFADSMDPSLDGYKRQWASGALSVTSVFNI

FIVIASSNEHSRKILNSPTHAEPCLTSSAKPILLKENWVFLHGKVHADYRKALNVLFTKK

ALSIYLPIQEKIYRKYFKSWLADPAPAAKPYMMQMRDLNMETSLRVFVGDHVSENAAQEI

SDKYWLITLALQLVNFPLAIPGTNVYNAIQARKIAMKHLTAAAASSKVSMARGDEPSCLL

DAWISEMISARQGAGEDEQRKVLSREYTDHEIAMVVLSFLFASQDAMTSAIVYLFQHMAD

HPEILEKVREEQYRIRGGDVDAPTTLEMVDDMVYTRACVKESLRLVPPVIMVPYKAKKPF

KISEDYTVPTGTMVIPSFWDSTHDSTAYPEPEKFLPERWLPTEMGEQPLADKYPQNYMVW

GSGPHKCIGVQYASMHLAAVIGSASVTMDWEHERTPQSDETQVLCTLFPKDGLRLKFTPR

APPS*

>CYP61A(402392)Cryptococcus curvatus

MSSQAIPRATSLADKTWGLDSLTKARFSFDSKTTAATILTVVISLLILEQIVYRTKKAHL

PGDKWTIPVIGKFADSLNPTLENYKRQWYSGPLSCVSVFNIFIVIGSSNKMARKILNSPN

HAEPCLVASAKKVLDPENWVFLHGKVHADYRKALNVLFTQKALAIYLPIQERIYRRFFKE

WVSYKGARPMMMRMRDLNMETSLSVFIGPYLTDADKDLINKKYWDITTALELVNFPFAIP

GTKVYNAIQARKVVMKYLMNASASSKVRMADMQNEPECLLDEWTRAMILSKNGNSEEAKL

LSREYSDKEIAMVVLSFLFASQDAMSSAIVYAFQLTADHPEILAKIREEQMRVRGGDLDA

PLTLDLVEDMVYTRAVVKEVLRLRPPVIMVPYLTTREFPITEDYTVPKGAMIIPAFWNSL

HDETCFPEPDKFMPERWLPNEDGSAPLAESKPQNYLVWGSGPHKCIGPQYAGMHLAATLG

TASVLLDWEHVRTKDSDEVKVIAAIFPKDEFIASFTERAAM*

>CYP61A(2740585997)Cryptococcus amylolentus CBS 6273

MASHAILNPTAIPDFDSIKTWGFEGLTKANFSFTFDTKTTTATLLTLLLSLLVLEQVVYR

SKKAHLPGNKWTIPLIGAFADSLNPTLANYKAQWDSGALSAVSVFNIFIVIGSSNEMARK

IMNSPNHAEPCLVASAKKVLLPENWVFGHGKGHADYRKALNVLFTKQALSTYLPIQEKIY

RSYFTKWMTDPQPAFPYMMPMRDLNMDTSLSVFCGPYISEEEKVDINKKYWLITLALELV

NFPFAIPGTKVYNAIQARKTVMKVLTRSSAESKIKMADFANEPECLLDEWTRAMINAQKA

QDDGETTKLLQREYSDHEIAMVLLSFLFASQDAMSSAIVYSFQLTADHPEVLAKIREEQY

RIRGNDLERGLTLDMIDDMVYTRAVVKEVLRIRPPVIMVPYLTTKPFPVSPEYTVPKNSM

IIPAFWNSLHDDAVYTNPDAFAPERWLPNSDGSSPLAESKPQNYMVWGSGPHKCIGVQYA

SMHLAATLGTASVLMDWEHEKTELSDDVQVIAAIFPKDHCKLKFTPRAPPA

>CYP61A(KGB76083.1)Cryptococcus gattii VGII R265

MESHTILRPTAIPDLAAIKTWGLEGLTKAKFSFDSKTTTATILTLILSLLVLEQLVYRAK

KAHLPGAKWTIPVIGKFADSLNPTLANYKAQWNSGPLSAVSVFNIFIVIGSSNEMARKIL

NSPNHAEPCLVASAKKVLLPENWVFLHGKVHADYRKALNVLFTKQALSIYLPIQEKIYRS

YFNKWTSDPAPAQQYMMKMRDLNMETSLSVFIGPYLTEAQKQEINEKYWLITISLELVNF

PLAIPGTKVYNAIQARKIVMKYLSAASAASKIRMEDDDAEPECLLDHWVRAMILARRAQD

DGEETKLLSREYSDHEIAMVLLSFLFASQDAMSSALVYTFQLTADHPEVLAKVREEQYRV

RGNDLERPLTLDLLDDMVYTRATIKEVLRFRPPVIMVPYMTTKPFPVSPEYTAPKNSMII

PAFWNSLHDETCYPEPDRFLPERWLPQADGSAPIADSKPQNYLVWGSGPHKCIGGQYASM

HLAATLGTASVLMDWKHERTELSDEVQVIAAIFPKDHCLLKFTPRAPPS

>CYP61A(KIY56719.1)Cryptococcus gattii VGII 99/473

MESHTILRPTAIPDLAAIKTWGLEGLTKAKFSFDSKTTTATILTLILSLLVLEQLVYRAK

KAHLPGAKWTIPVIGKFADSLNPTLANYKAQWNSGPLSAVSVFNIFIVIGSSNEMARKIL

NSPNHAEPCLVASAKKVLLPENWVFLHGKVHADYRKALNVLFTKQALSIYLPIQEKIYRS

YFNKWTSDPAPAQQYMMKMRDLNMETSLSVFIGPYLTEAQKQEINEKYWLITISLELVNF

PLAIPGTKVYNAIQARKIVMKYLSAASAASKIRMEDDDAEPECLLDHWVRAMILARRAQD

DGEETKLLSREYSDHEIAMVLLSFLFASQDAMSSALVYTFQLTADHPEVLAKVREEQYRV

RGNDLERPLTLDLLDDMVYTRATIKEVLRFRPPVIMVPYMTTKPFPVSPEYTAPKNSMII

PAFWNSLHDETCYPEPDRFLPERWLPQADGSAPIADSKPQNYLVWGSGPHKCIGGQYASM

HLAATLGTASVLMDWKHERTELSDEVQVIAAIFPKDHCLLKFTPRAPPS

>CYP61A(KIS00113.1)Cryptococcus gattii VGII 2001/935-1

MESHTILRPTAIPDLAAIKTWGLEGLTKAKFSFDSKTTTATILTLILSLLVLEQLVYRAK

KAHLPGAKWTIPVIGKFADSLNPTLANYKAQWNSGPLSAVSVFNIFIVIGSSNEMARKIL

NSPNHAEPCLVASAKKVLLPENWVFLHGKVHADYRKALNVLFTKQALSIYLPIQEKIYRS

YFNKWTSDPAPAQQYMMKMRDLNMETSLSVFIGPYLTEAQKQEINEKYWLITISLELVNF

PLAIPGTKVYNAIQARKIVMKYLSAASAASKIRMEDDDAEPECLLDHWVRAMILARRAQD

DGEETKLLSREYSDHEIAMVLLSFLFASQDAMSSALVYTFQLTADHPEVLAKVREEQYRV

RGNDLERPLTLDLLDDMVYTRATIKEVLRFRPPVIMVPYMTTKPFPVSPEYTAPKNSMII

PAFWNSLHDETCYPEPDRFLPERWLPQADGSAPIADSKPQNYLVWGSGPHKCIGGQYASM

HLAATLGTASVLMDWKHERTELSDEVQVIAAIFPKDHCLLKFTPRAPPS

>CYP61A(KIR93845.1)Cryptococcus gattii VGII CBS 10090

MESHTILRPTAIPDLAAIKTWGLEGLTKAKFSFDSKTTTATILTLILSLLVLEQLVYRAK

KAHLPGAKWTIPVIGKFADSLNPTLANYKAQWNSGPLSAVSVFNIFIVIGSSNEMARKIL

NSPNHAEPCLVASAKKVLLPENWVFLHGKVHADYRKALNVLFTKQALSIYLPIQEKIYRS

YFNKWTSDPAPAQQYMMKMRDLNMETSLSVFIGPYLTEAQKQEINEKYWLITISLELVNF

PLAIPGTKVYNAIQARKIVMKYLSAASAASKIRMEDDDAEPECLLDHWVRAMILARRAQD

DGEETKLLSREYSDHEIAMVLLSFLFASQDAMSSALVYTFQLTADHPEVLAKVREEQYRV

RGNDLERPLTLDLLDDMVYTRATIKEVLRFRPPVIMVPYMTTKPFPVSPEYTAPKNSMII

PAFWNSLHDETCYPEPDRFLPERWLPQADGSAPIADSKPQNYLVWGSGPHKCIGGQYASM

HLAATLGTASVLMDWKHERTELSDEVQVIAAIFPKDHCLLKFTPRAPPS

>CYP61A(KJE04731.1)Cryptococcus gattii NT-10

MESHTILRPTAIPDLAAIKTWGLEGLTKAKFSFDSKTTTATVLTLILSLLVLEQLVYRAK

KAHLPGAKWTIPVIGKFADSLNPTLANYKAQWNSGPLSAVSVFNIFIVIGSSNEMARKIL

NSPNHAEPCLVASAKKVLLPENWVFLHGKVHADYRKALNVLFTKQALSIYLPIQERIYRS

YFNKWMSDPAPAQQYMMKMRDLNMETSLSVFIGPYLTEAQKQEINEKYWLITISLELVNF

PLAIPGTKVYNAIQARKIVMKYLSAASAASKIRMEDDDAEPECLLDHWVRAMILARRAQD

DGEETKLLSREYSDHEIAMVLLSFLFASQDAMSSALVYTFQLTADHPEVLAKVREEQYRV

RGNDLERPLTLDLLDDMVYTRATIKEVLRFRPPVIMVPYMTTKPFPVSPEYTAPKNSMII

PAFWNSLHDETCYPEPDRFLPERWLPQADGSAPIADSKPQNYLVWGSGPHKCIGGQYASM

HLAATLGTASVLMDWKHERTELSDEVQVIAAIFPKDHCLLKFTPRAPPS

>CYP61A(KIY32265.1)Cryptococcus gattii E566

MESHTILRPTAIPDLAAIKTWGLEGLTKAKFSFDSKTTTATVLTLILSLLVLEQLVYRAK

KAHLPGAKWTIPVIGKFADSLNPTLANYKAQWNSGPLSAVSVFNIFIVIGSSNEMARKIL

NSPNHAEPCLVASAKKVLLPENWVFLHGKVHADYRKALNVLFTKQALSIYLPIQERIYRS

YFNKWMSDPAPAQQYMMKMRDLNMETSLSVFIGPYLTEAQKQEINEKYWLITISLELVNF

PLAIPGTKVYNAIQARKIVMKYLSAASAASKIRMEDDDAEPECLLDHWVRAMILARRAQD

DGEETKLLSREYSDHEIAMVLLSFLFASQDAMSSALVYTFQLTADHPEVLAKVREEQYRV

RGNDLERPLTLDLLDDMVYTRATIKEVLRFRPPVIMVPYMTTKPFPVSPEYTAPKNSMII

PAFWNSLHDETCYPEPDRFLPERWLPQADGSAPIADSKPQNYLVWGSGPHKCIGGQYASM

HLAATLGTASVLMDWKHERTELSDEVQVIAAIFPKDHCLLKFTPRAPPS

>CYP61A(KIR82846.1)Cryptococcus gattii EJB2

MESHTILRPTAIPDLAAIKTWGLEGLTKAKFSFDSKTTTATVLTLILSLLVLEQLVYRAK

KAHLPGAKWTIPVIGKFADSLNPTLANYKAQWNSGPLSAVSVFNIFIVIGSSNEMARKIL

NSPNHAEPCLVASAKKVLLPENWVFLHGKVHADYRKALNVLFTKQALSIYLPIQERIYRS

YFNKWMSDPAPAQQYMMKMRDLNMETSLSVFIGPYLTEAQKQEINEKYWLITISLELVNF

PLAIPGTKVYNAIQARKIVMKYLSAASAASKIRMEDDDAEPECLLDHWVRAMILARRAQD

DGEETKLLSREYSDHEIAMVLLSFLFASQDAMSSALVYTFQLTADHPEVLAKVREEQYRV

RGNDLERPLTLDLLDDMVYTRATIKEVLRFRPPVIMVPYMTTKPFPVSPEYTAPKNSMII

PAFWNSLHDETCYPEPDRFLPERWLPQADGSAPIADSKPQNYLVWGSGPHKCIGGQYASM

HLAATLGTASVLMDWKHERTELSDEVQVIAAIFPKDHCLLKFTPRAPPS

>CYP61A(KIR84120.1)Cryptococcus gattii VGIV IND107

MESHTILRPTAIPDLAAIKTWGLEGLTKAKFSFDSKTTTATILTLILSLLVLEQLVYRAK

KAHLPGAKWTIPVIGKFADSLNPTLANYKAQWNSGPLSAVSVFNIFIVIGSSNEMARKIL

NSPNHAEPCLVASAKKVLLPENWVFLHGKVHADYRKALNVLFTKQALSIYLPIQERIYRS

YFNKWMSDPAPAQQYMMKMRDLNMETSLSVFIGPYLTEAQKQEINEKYWLITISLELVNF

PLAIPGTKVYNAIQARKIVMKYLSAASAASKIRMEDDDAEPECLLDHWVRAMILARRAQD

DGEETKLLSREYSDHEIAMVLLSFLFASQDAMSSALVYTFQLTADHPEVLAKVREEQYRV

RGNDLERPLTLDLLDDMVYTRATIKEVLRFRPPVIMVPYMTTKPFPVSPEYTAPKNSMII

PAFWNSLHDETCYPEPDRFLPERWLPQADGSAPIADSKPQNYLVWGSGPHKCIGGQYASM

HLAATLGTASVLMDWKHERTELSDEVQVIAAIFPKDHCLLKFTPRTPPS

>CYP61A(AFR95929.1)Cryptococcus neoformans var. grubii H99

MESHTILRPTAIPDLAAIKTWGLEGLTKAKFSFDTKTTTATILTLILSLLVLEQLVYRAK

KAHLPGAKWTIPVIGKFADSLNPTLANYKAQWNSGPLSAVSVFNIFIVIGSSNEMARKIL

NSPNHAEPCLVASAKKVLLPENWVFLHGKVHADYRKALNVLFTKQALSIYLPIQEKIYRS

YFNKWMSDPAPAQQYMMKMRDLNMDTSLSVFIGPYLTEAQKQEINDKYWLITISLELVNF

PLAIPGTKVYNAIQARKTVMKYLSAASAASKIRMEDDEAEPECLLDHWVRAMILARRAKD

DGEQTRLLSREYSDHEIAMVLLSFLFASQDAMSSALVYTFQLTADHPEVLEKVREEQYRV

RGNDLERPLTLDMLDDMVYTRATIKEVLRFRPPVIMVPYTTKPFPVSPEYTAPKNSMIIP

AFWNSLHDETCYPEPDRFLPERWLPQADGSPPIADSKPQNYLVWGSGPHKCIGGQYASMH

LAATLGTASVLMDWKHERTELSDEVQVIAAIFPKDHCLLKFTPRAPPS

>CYP61A(EAL20298.1)Cryptococcus neoformans var. neoformans B-3501A

MESHTVLRPTAIPDLAAIKTWGLEGLTKAKFSFDTKTTTATILTLILSLLVLEQLVYRAK

KAHLPGAKWTIPVIGKFADSLNPTLANYKAQWNSGPLSAVSVFNIFIVIGSSNEMARKIL

NSPNHAEPCLVASAKKVLLPENWVFLHGKVHADYRKALNVLFTKQALSIYLPIQEKIYRS

YFNKWMSDPAPAQQYMMKMRDLNMDTSLSVFIGPYLTEAQKQEINDKYWLITISLELVNF

PLAIPGTKVYNAIQARKIVMKYLSAASAASKIRMEDDDAEPECLLDHWVRAMILARRAKD

DGEQTRLLSREYSDHEIAMVLLSFLFASQDAMSSALVYTFQLTADHPEVLEKVREEQYRV

RGNDLERPLTLDMLDDMVYTRATIKEVLRFRPPVIMVPYMTTKPFPVSPEYTAPKNSMII

PAFWNSLHDETCYPEPDRFLPERWLPQADGSAPIADSKPQNYLVWGSGPHKCIGGQYASM

HLAATLGTASVLMDWKHERTELSDEVQVIAAIFPKDHCLLKFTPRAPPS

>CYP61A(AAW44138.1)Cryptococcus neoformans var. neoformans JEC21

MESHTVLRPTAIPDLAAIKTWGLEGLTKAKFSFDTKTTTATILTLILSLLVLEQLVYRAK

KAHLPGAKWTIPVIGKFADSLNPTLANYKAQWNSGPLSAVSVFNIFIVIGSSNEMARKIL

NSPNHAEPCLVASAKKVLLPENWVFLHGKVHADYRKALNVLFTKQALSIYLPIQEKIYRS

YFNKWMSDPAPAQQYMMKMRDLNMDTSLSVFIGPYLTEAQKQEINDKYWLITISLELVNF

PLAIPGTKVYNAIQARKIVMKYLSAASAASKIRMEDDDAEPECLLDHWVRAMILARRAKD

DGEQTRLLSREYSDHEIAMVLLSFLFASQDAMSSALVYTFQLTADHPEVLEKVREEQYRV

RGNDLERPLTLDMLDDMVYTRATIKEVLRFRPPVIMVPYMTTKPFPVSPEYTAPKNSMII

PAFWNSLHDETCYPEPDRFLPERWLPQADGSAPIADSKPQNYLVWGSGPHKCIGGQYASM

HLAATLGTASVLMDWKHERTELSDEVQVIAAIFPKDHCLLKFTPRAPPS

>CYP5216A(AAW42401.1)Cryptococcus neoformans var. neoformans JEC21

MKGGRFLLPALFKTLLLPPLIAALFIHHFPSLFLPFSALIYLISFPALYVFRSYASLVAS

SWKASALGAIDIPRVNGTWPLNIDILLNWAKSGTEEEVGRMMVLMGRQYGGTYNTRVLGE

DQIISSDPKVIKHVLIDDFDNFVKGQKFKERAQDFLGDGIFNSDGDNWKFHRSFMRPFFQ

PKYISPLHFSTNVQNFFAKLPSYGKAFDMQARIGQLALELAIMWLCGEDMSGDASNVART

EEWKKAKGEISWAMTEAQKIVGKRVKIGTIWPLFEVRHDPLERPMKVIRAFFRPIISRAL

NRKRQRCKLDNTEDVYMIDRLVEATDDIKLVEDQLINVLLASRDTLASLLTFSVYAIVLH

PDFAARLKDEVFTIANQECEVTKDTIRQLRYCRAFINEVLRLFPPVPLNIRRTLRPSLLP

TPNHLAYMPANTSIILATILMQRDPAVWGEDAQVFNPDRWLEGGGLGKEKEGFMSWNLGP

RMCLGQSFALAITHTFLVYFFRHIAHVGTLVGHNTTVQLALDAQPPEVLPKEWMTPEGGD

GRARGGRDKVWIVADVVLAIKGGLWIRFGAEETE

>CYP5216A(CGB_C6680W)Cryptococcus gattii WM276

MKGGQFLLPALFKTLILPPLIAALFTHRFHSYSLSLTALIYLISFPAFYVFRSYVSLVTS

SRKASALGAVDIPRVKGAWPLNIDVLLNWAKSGTEEEVGRMMVLMGRQYGETYNTRVLGE

DQIISSDPKVIKHVLTDDFDNFVKGQKFKDRAQDFLGDGIFNSDGDGWKFHRSLMRPFFH

PTYISLLHFTGNVQNFFISLPSYGKAFDMQACIGQLALELAIMWLCGEDMSADASNVERT

EEWWQAKREIGWAMTEAQKAVGKRVKIGTVWPLFEITHDPLERPMKVIRAFFRPIISQAL

NRKRQRCKLDDQEDVYMIDRLVEATDDVKLVEDQLINVLLASRDTLASLLTFSVYAIALH

PDIADRLKNEIFSVACRESEVTKDTIRQLRYSRAFINEVLRLFPPVPLNIRRTLRPSLLP

TTNHLAYMPANTSIILATILMQRDPAVWGEDALVFNPDRWLGEGLGKERESFASWNLGPR

MCLGQPFALTITHTFLVYFFRHIAHVGTLAGQNTTIQLALDAQPPETVLPKEWMTSEGSD

GRTRGGRDRVWIVADVVLAIKGGLWVRFGAENTE

>CYP5216A1(EAL22122.1)Cryptococcus neoformans var. neoformans B-3501A

MKGGRFLLPALFKTLLLPPLIAALFIHHFPSLFPTFTALIYLISFPALYVFRSYASLVAS

SWKASALGAIDIPRVNGTWPLNIDILLNWAKSGTEEEVGRMMVLMGRQYGGTYNTRVLGE

DQIISSDPKVIKHVLIDDFDNFVKGQKFKERAQDFLGDGIFNSDGDNWKFHRSFMRPFFQ

PKYISPLHFSTNVQNFFAKLPSYGKVFDMQAHIGQLALELAIMWLCGEDMSRDTNNVAWT

EEWKKAKGEISWAMTEAQKIVGKRVKIGTIWPLFEVRHDPLERPMKVIRAFFRPIISRAL

NRKRQRCKLDNTEDVYMIDRLVEATDDIKLVEDQLINVLLASRDTLASLLTFSVYAIVLH

PDIAVRLKDEVFTIANQECEVTKDTIRQLRYCRAFINEVLRLFPPVPLNIRRTLRPSLLP

TPNHLAYMPANTSIILATILMQRDPAVWGEDAQVFNPDRWLEGGGLGKEKEGFMSWNLGP

RMCLGQSFALAITHTFLVYFFRHIAHVGTLVGHNTTVQLALDAQPPETVLPKEWMTPEGG

DGRARGGRDKVWIVADVVLAIKGGLWIRFGAEETE

>CYP5216A2(AFR93852.1)Cryptococcus neoformans var. grubii H99

MKGGRFLLPALFKTLLLPPLIAALFTHHFPSLSLSFTALIYLLSFPALYVFRSYTSLVAS

SWKASALGAIDIPRVRGTWPLNIDVLLNWAKSGTADEVGRMMVLMVRQYGGTYNTRVLGE

DQIISSDPRVIKHVLIDDFDNFVKGQKFKERAQDFLGDGIFNSDGDNWKFHRSFMRPFFH

PTYISPLHLATSVQNFFTRLPSYGEAFDIQARIGQLALELAMMWLCGEDMSAGASNVART

EEWKRAKGQISWAMTEAQKIVGKRVKIGTVWPLFELTHDPLERPMKVIRAFFRPIISEAL

NRKRQRCKLDNTEDVYMIDRLVEATDDIKLVEDQLINVFLASRDTLASLLTFSAYAIVLH

PDIAARLKDEVFTVANQESEVTKDTVRQLRYCRAFINEVRLFPPVPLNIRRTLRPSLLPT

PNHLAYMPANTSIILATILMQRDPAVWGEDAQVFNPDRWLEGGLGKEREGFVSWNLGPRM

CLGQSLALTITHTFLVYFFRHIAHVGTLVGQNITTQLAPDAQPPETALPKEWMTLEGGDG

RARGGRDRVWIVADVVLAIKGGLWIRFGAEGTE

>CYP5216A(KIR81701.1)Cryptococcus gattii EJB2

MKGGQFLLPALFKTLILPPLIAALFTHRFHSYSLSLTALIYLISFPAFYVFRSYVSLVTS

SRKASALGAVDIPRVKGAWPLNIDVLLNWAKSGTEEEVGRMMVLMGRQYGETYNTRVLGE

DQIISSDPKVIKHVLTDDFDNFVKGQKFKDRAQDFLGDGIFNSDGDGWKFHRSLMRPFFH

PTYISLLHFTGNVQNFFISLPSYGKAFDMQACIGQLALELAIMWLCGEDMSADASNVERT

EEWWQAKREIGWAMTEAQKAVGKRVKIGTVWPLFEITHDPLERPMKVIRAFFRPIISQAL

NRKRQRCKLDDQEDVYMIDRLVEATDDVKLVEDQLINVLLASRDTLASLLTFSVYAIALH

PDIADRLKNEIFSVACRESEVTKDTIRQLRYSRAFINEVLRLFPPVPLNIRRTLRPSLLP

TTNHLAYMPANTSIILATILMQRDPAVWGEDALVFNPDRWLGEGLGKERESFASWNLGPR

MCLGQPFALTITHTFLVYFFRHIAHVGTLAGQNTTIQLALDAQPPETVLPKEWMTSEGSD

GRTRGGRDRVWIVADVVLAIKGGLWVRFGAENTE

>CYP5216A(KIY36728.1)Cryptococcus gattii E566

MKGGQFLLPALFKTLILPPLIAALFTHRFHSYSLSLTALIYLISFPAFYVFRSYVSLVTS

SRKASALGAVDIPRVKGAWPLNIDVLLNWAKSGTEEEVGRMMVLMGRQYGETYNTRVLGE

DQIISSDPKVIKHVLTDDFDNFVKGQKFKDRAQDFLGDGIFNSDGDGWKFHRSLMRPFFH

PTYISLLHFTGNVQNFFISLPSYGKAFDMQACIGQLALELAIMWLCGEDMSADASNVERT

EEWWQAKREIGWAMTEAQKAVGKRVKIGTVWPLFEITHDPLERPMKVIRAFFRPIISQAL

NRKRQRCKLDDQEDVYMIDRLVEATDDVKLVEDQLINVLLASRDTLASLLTFSVYAIALH

PDIADRLKNEIFSVACRESEVTKDTIRQLRYSRAFINEVLRLFPPVPLNIRRTLRPSLLP

TTNHLAYMPANTSIILATILMQRDPAVWGEDALVFNPDRWLGEGLGKERESFASWNLGPR

MCLGQPFALTITHTFLVYFFRHIAHVGTLAGQNTTIQLALDAQPPETVLPKEWMTSEGSD

GRTRGGRDRVWIVADVVLAIKGGLWVRFGAENTE

>CYP5216A(KJE01980.1)Cryptococcus gattii NT-10

MKGGQFLLPALFKTLILPPLIAALFTHRFHSYSLSLTALIYLISFPAFYVFRSYVSLVTS

SRKASALGAVDIPRVKGAWPLNIDVLLNWAKSGTEEEVGRMMVLMGRQYGETYNTRVLGD

QIISSDPKVIKHVLTDDFDNFVKGQKFKDRAQDFLGDGIFNSDGDGWKFHRSLMRPFFHP

TYISLLHFTGNVQNFFISLPSYGKAFDMQACIGQLALELAIMWLCGEDMSADASNVERTE

EWWQAKREIGWAMTEAQKAVGKRVKIGTVWPLFEITHDPLERPMKVIRAFFRPIISQALN

RKRQRCKLDDQEDVYMIDRLVEATDDVKVEDQLINVLLASRDTLASLLTFSVYAIALHPD

IADRLKNEIFSVACRESEVTKDTIRQLRYSRAFINEVLRLFPPVPLNIRRTLRPSLLPTT

NHLAYMPANTSIILATILMQRDPAVWGEDALVFNPDRWLGEGLGKERESFASWNLGPRMC

LGQPFALTITHTFLVYFFRHIAHVGTLAGQNTTIQLALDAQPPETVLPKEWMTSEGSDGR

TRGGRDRVWIVADVVLAIKGGLWVRFGAENTE

>CYP5216A(KIR87319.1)Cryptococcus gattii VGIV IND107

MKRGRFLLLALFKTLILPPLIAALFTHHFPSLSLSLTALIYLISFPALYVFRSYVSLVTS

SRKASALGAVDIPRVKGAWPLNIDVLLNWAKSGTEEEVGRMMVLMGRQYGETYNTRVLGE

DQIISSDPKVIKHVLIDDFDNFVKGQKFKDRAQDFLGDGIFNSDGDGWKFHRSLMRPFFH

PTYISPLHFTVNVQNFFISLPSYGKAFDMQACIGQLALELAIMWLCGEDMSADASNVERT

EEWGKAKREIGWAMTEAQKVVGKRVKIGTVWPLFEIIHDPLERPMKVIRAFFRPIISQAL

NRKRQRCKLDDQEDAYMIDRLVEATDDIKLVEDQLINVLLASRDTLASLLTFSVYAIALH

PHIAARLKNEIFSVACRESEVTKDTIRQLRYSRAFINEVLRLFPPVPLNIRRTLRPSLLP

TTNHLAYMPANTSIILATILMQRDPAVWGGDALVFNPDRWLGGGLGKERESFASWNLGPR

MCLGQQFALAITHTFLVYFFRHIAHVGTLAGQNTTIQLALDAQPPETVLPKRWMTSEGSD

GRTRGGRDRVWIVADVVLAVKGGLWVRFGAENTEW

>CYP5216A(KIY57186.1)Cryptococcus gattii VGII 99/473

MKGGRFLLPALFKTLILPPLIAALFTHHFPSLSLSLTALIYLISLPALYVFRSYVSLVTS

SRRAAALGAVDIPRVKGAWPLNIDVLLNWAKSGTEEEVGRMMVLMGRQYGGTYNTRVLGE

DQIISSDPKVIKHVLIDDFDNFVKGQKFKDRAQNFLGDGIFNSDGDGWKFHRSLMRPFFH

PMYISPLHFTVNIQAFFNSLPLYGKAFDMQACIGQLALELAIMWLCGEDMSADASNVERT

DEWRKAKREIGWAMTEAQKVVGKRVKIGTVWPLFEITHDPLERPMKVIRAFFRPIISQAL

NRKRQRCKSDDQEDAYMIDRLVEATDDVKLVEDQLINVLLASRDTLASLLIFSVYAIALH

PDIAARLKNEIFSVASRESEVTKDTIRQLRYSRAFINEVLRLFPPVPLNIRRTLRPSLLP

TTSHLVYMPANTSIILATILMQRDPAVWGEDALVFNPDRWLAGGLGKQRENFASWNLGPR

MCLGQPFALTITHTFLVYFFRHIAHVGTLVGQNTTIQLALDAQPPKTLLPKEWMTSEGSD

GRTRGGRDRVWIVADVVLAIKGGLWVRFGAENTE

>CYP5216A3(KGB77653.1)Cryptococcus gattii VGII R265

MKGGRFLLPALFKTLILPPLIAALFTHHFPSLSLSLTALIYLISLPALYVFRSYVSLVTS

SRRAAALGAVDIPRVKGAWPLNIDVLLNWAKSGTEEEVGRMMVLMGRQYGGTYNTRVLGE

DQIISSDPKVIKHVLIDDFDNFVKGQKFKDRAQNFLGDGIFNSDGDGWKFHRSLMRPFFH

PMYISPLHFTVNIQAFFNSLPLYGKAFDMQACIGQLALELAIMWLCGEDMSADASNVERT

DEWRKAKREIGWAMTEAQKVVGKRVKIGTVWPLFEITHDPLERPMKVIRAFFRPIISQAL

NRKRQRCKSDDQEDAYMIDRLVEATDDVKLVEDQLINVLLASRDTLASLLIFSVYAIALH

PDIAARLKNEIFSVASRESEVTKDTIRQLRYSRAFINEVLRLFPPVPLNIRRTLRPSLLP

TTSHLVYMPANTSIILATILMQRDPAVWGEDALVFNPDRWLAGGLGKQRENFASWNLGPR

MCLGQPFALTITHTFLVYFFRHIAHVGTLVGQNTTIQLALDAQPPKSLLPKEWMTSEGSD

GRTRGGRDRVWIVADVVLAIKGGLWVRFGAENTE

>CYP5216A(KIR92342.1)Cryptococcus gattii VGII CBS 10090

MKGGRFLLPALFKTLILPPLIAALFTHHFPSLSLSLTALIYLISLPALYVFRSYVSLVTS

SRRAAALGAVDIPRVKGAWPLNIDVLLNWAKSGTEEEVGRMMALMGRQYGGTYNTRVLGE

DQIISSDPKVIKHVLIDDFDNFVKGQKFKDRAQNFLGDGIFNSDGDGWKFHRSLMRPFFH

PMYISPLHFTVNIQAFFNSLPLYGKAFDMQACIGQLALELAIMWLCGEDMSADASNVERT

DEWRKAKREIGWAMTEAQKVVGKRVKIGTVWPLFEITHDPLERPMKVIRAFFRPIISQAL

NRKRQRCKSDDQEDAYMIDRLVEATDDVKLVEDQLINVLLASRDTLASLLIFSVYAIALH

PDIAARLKNEIFSVASRESEVTKDTIRQLRYSRAFINEVLRLFPPVPLNIRRTLRPSLLP

TTSHLVYMPANTSIILATILMQRDPAVWGEDALVFNPDRWLAGGLGKQRENFASWNLGPR

MCLGQPFALTITHTFLVYFFRHIAHVGTLVGQNTTIQLALDAQPPKTLLPKEWMTSEGSD

GRTRGGRDRVWIVADVVLAIKGGLWVRFGAENTE

>CYP5216A(KIS01508.1)Cryptococcus gattii VGII 2001/935-1

MKGGRFLLPALFKTLILPPLIAALFTHHFPSLSLSLTALIYLISLPALYVFRSYVSLVTS

SRRAAALGAVDIPRVKGAWPLNIDVLLNWAKSGTEEEVGRMMVLMGRQYGGTYNTRVLGE

DQIISSDPKVIKHVLIDDFDNFVKGQKFKDRAQNFLGDGIFNSDGDGWKFHRSLMRPFFH

PMYISPLHFVNIQAFFNSLPLYGKAFDMQACIGQLALELAIMWLCGEDMSADASNVERTD

EWRKAKREIGWAMTEAQKVVGKRVKIGTVWPLFEITHDPLERPMKVIRAFFRPIISQALN

RKRQRCKSDDQEDAYMIDRLVEATDDVKLVEDQLINVLLASRDTLASLLIFSVYAIALHP

DIAARLKNEIFSVASRESEVTKDTIRQLRYSRAFINEVLRLFPPVPLNIRRTLRPSLLPT

TSHLVYMPANTSIILATILMQRDPAVWGEDALVFNPDRWLAGGLGKQRENFASWNLGPRM

CLGQPFALTITHTFLVYFFRHIAHVGTLVGQNTTIQLALDAQPPKTLLPKEWMTSEGSDG

RTRGGRDRVWIVADVVLAIKGGLWVRFGAENTE

>CYP51F1(809380)(Cryptococcus terricola)

MDPYKFMFGCRAKYGDLFTFILMGREMTVALGPKGNNLILGGKLSQVSAEEAYTHLTTPV

FGKGVVYDCPNDMLMQQKRMVKFGLSTENLKSYVPKITRETLDFLSHGLHTSATKWQSFN

ALDKLAELITLTASDCLQGREVRAGLDKTFAKRIEALDKGFTPMNFVFPNLPLPAYWNRD

KAQKEMSEFYQEIIRKRREGTHDHEHDMLEALMGSSYKNGTPLTDSDIAHMMIALLMAGQ

HTSSATSSWTLLHLAQRHDIYEALYQEQKEKFGNADGTFRDLTYEDLKDLPLLDSCIRET

LRLHAPLHSIMRKVISDIPVPNSLSSPSQTNDASYVIPKGHFLMACPGVSQMDPLIWQDS

TTWNPYRWSDERGVAAQAVEEYQGEKGEKVDYGFGSVSKGTESPYQPFGAGRHRCIGESF

AYVQLQTIIATVVRNLTLKFEGDFPSPNYQTMVVLPKGGEILFKSRQ*

>CYP51F(629036)Cryptococcus werringae

MRLSTCLAVATAIVAPAKAIIQHPVSVYLLPSPDASPSTVKAPTLDGDEAFDVFANHFGV

GSQSTFEKYQKKSWAHLVDPSHSSELTGEEQAKVIIIQGDLSPSDILPPTYQDSLADFEL

PSFPSYETMNFLQPFMIRGQKVVGHVLGENQDGLEEGFDRVWSNVKGSQVGKKMASAFDL

NTSLHAQALHAELQALSALAMNLPVDARLERNRNVWDSVVISSLGTNDSTGVDAEVREMG

LNVIRASLQALAANPKQPRVVLVVVPTTSGAAFAPYVAAVKKFFTRLTTSSDQSILLGFL

PTLFSSFGTIGSVLLLLLSLPVLLVAGNVFRQKVLPRDPTLPPLVFHYIPWFGSAATYGM

DPYKFLFDCREQYGDLFTFILLGRKMTVALGPKGNNLILGGKLSQVSAEDAYTHLTTPVF

GKGVVYDCPNEMLMQQKRMVKFGLSTENFRVYVPLIKQETLDFLGKDLKPSNEWKGFNAL

HAMAELTILTASATLQGKEVRGKLDKTFAKRYEALDGGFTPINFMFPNLPLPSYKRRDKA

QAEMSEFYQSIIRGRREGASEHDHDMIAALMASTYRDGTPLSDSDIAHMMIALLMAGQHT

SSATSSWTLLHLADRTDVYAELYEEQKEKFGNPDGTFRDMTYEDLKDLPKLDSVIRETLR

MHAPIHSIMRKVMTDMPVPNALAAPTANEKSSYIVPKGHFVLASPGVAQMDPLIWSDASI

WNPHRWTDEKGMASQALEEYTSGDKVDYGYGSVSKGTESPYQPFGAGRHRCIGETFAYLQ

LQTILATFIRNVEMKLDGPFKTNYQTMVVLPLPGTKILYRNRK*

>CYP51F(367271)Cryptococcus curvatus

MAMDPSAVLDWAHALPLPVKIVGAVIGVPLLVIVLNVLRQLIVPTDPTAPPVVFHYIPWF

GSAAYYGMDPYKFMFECRDKYGDVFTFILLGRRMTVALGPKGNNLSLGGKVSHVSAEDAY

THLTTPVFGKGVVYDCPNDMLMQQKKFIKHGLTTEALQSYASLMPAECHGYFNNELKITK

ANPGPKTVDVLHVMAELIVLTASRTLQGKEVRENMSIRFAKLLEDLDKGFTPLNFMFPNL

PLPSYRRRDLAQKEMSDFYMSIMAKRRTGEHDHEPDMIQALQGSVYRNGTPLSDRDIAHM

MIALTMAGQHTSSATSSWFLLHLAHDQDIQKRLYEEQVEYFGNPDGTFRPMTYEDTKKLP

LMDSCIRETLRLHAPIHSIYRKVLQPIVVPQSLAAPSEDKAYVIPKGNFIVAAPGVSQMD

PKIWDDAPRWNPLRWLVDGGIAKTANEQYQAGEKVDYGFGAVSKGTESPYQPFGAGRHRC

IGEQFAYLQLTLVVGEVVRNYKLTAAQREFPKTNYQTMIVLPLDPKITFEARV*

>CYP51F(2740583322)Cryptococcus amylolentus CBS 6273

MSAIVPQLQRIAHEAGAYVPGWYTALPLALRVLVTVVGSIATIIGLNVLQQLVIPKRKDL

PPVVFHYIPWFGSAAYYGEDPYKFMFECRDKYGDVFTFILMGRRVTVALGPKGNNLSLGG

KISQVSAEDAYTHLTTPVFGKGVVYDCPNEMLMQQKKFIKSGLTTESLQSYPPLITAECE

EFFTKEAKITPSSPSATMDLLKAMSELIILTASRTLQGKEVRESLNGHFAKYYEDLDGGF

TPLNFMFPNLPLPSYKRRDDAQKAMSNFYIKIMEDRRKGESDHDQDMIANLQNCKYRNGV

ALTDRDISHIMIALLMAGQHTSSATSTWTLLHLADRPDIVEGLYKEQKEKLTNPDGSFKE

YKYEDLKELPLMDAIIRETLRLHAPIHSIYRKVMSDIPVPASLSAPSENSTYVIPKGHYI

LAAPGVSQMDPRIWADSTTWNPARWLETGGVAATAADEYTKGDQVDYGFGAVSKGTESPY

QPFGAGRHRCVGEQFAYTQLSTIFTYVIRNFELKLAVPEFPKTNYRTMIVQPHNPLVTFT

LRQPEKQEV

>CYP51F1(EAL23379.1)Cryptococcus neoformans var. neoformans B-3501A

MSAIIPQVQQLLGQVAQFFPPWFAALPTSLKVAIAVVGIPALIIGLNVFQQLCLPRRKDL

PPVVFHYIPWFGSAAYYGEDPYKFLFECRDKYGDLFTFILMGRRITVALGPKGNNLSLGG

KISQVSAEEAYTHLTTPVFGKGVVYDCPNEMLMQQKKFIKSGLTTESLQSYPPMITSECE

DFFTKEVGISPQKPSATLDLLKAMSELIILTASRTLQGKEVRESLNGQFAKYYEDLDGGF

TPLNFMFPNLPLPSYKRRDEAQKAMSDFYLKIMENRRKGESDHEHDMIENLQSCKYRNGV

PLSDRDIAHIMIALLMAGQHTSSATSSWTLLHLADRPDVVEALYQEQKQKLGNPDGTFRD

YRYEDLKELPIMDSIIRETLRMHAPIHSIYRKVLSDIPVPPSLSAPSENGQYIIPKGHYI

MAAPGVSQMDPRIWQDAKVWNPARWHDEKGFAAAAMVQYTKAEQVDYGFGSVSKGTESPY

QPFGAGRHRCVGEQFAYTQLSTIFTYVVRNFTLKLAVPKFPETNYRTMIVQPNNPLVTFT

LRNAEVKQEV

>CYP51F(AAW40645.1)Cryptococcus neoformans var. neoformans JEC21

MSAIIPQVQQLLGQVAQFFPPWFAALPTSLKVAIAVVGIPALIIGLNVFQQLCLPRRKDL

PPVVFHYIPWFGSAAYYGEDPYKFLFECRDKYGDLFTFILMGRRITVALGPKGNNLSLGG

KISQVSAEEAYTHLTTPVFGKGVVYDCPNEMLMQQKKFIKSGLTTESLQSYPPMITSECE

DFFTKEVGISPQKPSATLDLLKAMSELIILTASRTLQGKEVRESLNGQFAKYYEDLDGGF

TPLNFMFPNLPLPSYKRRDEAQKAMSDFYLKIMENRRKGESDHEHDMIENLQSCKYRNGV

PLSDRDIAHIMIALLMAGQHTSSATSSWTLLHLADRPDVVEALYQEQKQKLGNPDGTFRD

YRYEDLKELPIMDSIIRETLRMHAPIHSIYRKVLSDIPVPPSLSAPSENGQYIIPKGHYI

MAAPGVSQMDPRIWQDAKVWNPARWHDEKGFAAAAMVQYTKAEQVDYGFGSVSKGTESPY

QPFGAGRHRCVGEQFAYTQLSTIFTYVVRNFTLKLAVPKFPETNYRTMIVQPNNPLVTFT

LRNAEVKQEV

>CYP51F(CGB_B0330W)Cryptococcus gattii WM276

MSAIIPQVQQLLGQLAQYIPPWFTALPTSLKIVIAVIGIPAFIIGLNVFQQLCLPRTKDL

PPVVFHYIPWFGSAAYYGEDPYKFLFECRDKYGDLFTFILMGRRITVALGPKGNNLSLGG

KISQVSAEEAYTHLTTPVFGKGVVYDCPNEMLMQQKKFIKSGLTTESLQSYPPMITSECE

DFFTKEVGISSQKPSATLDLLKSMSELIILTASRTLQGKEVRESLNGQFAKYYEDLDGGF

TPLNFMFPNLPLPSYRRRDEAQKAMSDFYLKIMENRRKGESDHEHDMIENLQGCKYRNGV

PLSDRDVAHIMIALLMAGQHTSSATSSWTLLHLADRPDIVEALYQEQKEKLGNPDGTFRD

YKYEDLKELPIMDSIIRETLRMHAPIHSIYRKVLSDIPVPPSLAAPSENGQYIIPKGHYI

MAAPGVSQMDPRIWQDAKVWNPARWHDEKGFAAAAMAQYTKAEQVDYGFGSVSKGTESPY

QPFGAGRHRCVGEQFAYTQLSTIFTYVVRNFTLKLAVPKFPETNYRTMIVQPTNPLVTFT

LRNAEVKQEV

>CYP51F(345805) Naganishia vishniacii v1.0

MSSSRILSLLPLEQLSWASIAAIILVGLPALLVVGNIIRQKVLPKDPSLPPVVFHLIPWF

GSAATYGMDPYKFLFDCREQYGDLFTFVLLGRKMTVALGPKGNNLILGGRLNQVSAEEAY

THLTTPVFGEGVVYDCSNDLLMQQKRMVKFGLSTENLRAYVGMITEETLGFLTNELKASP

TAWQGFDALNAMSELTILTASRTLQGKEVRAGLDKTFAERYEHLDGGFTPINFMFPNLPL

PSYRRRDKAQKAMSEFYQGIIRKRREGTHDHEYDMISALQSSKYKDGTPLSDRDIAHMMI

ALLMAGQHTSSATSSWTLLHLAERTDIWEELYREQKNKFGNPDGTFRDLTYEELKELPVL

DHVIRETLRLHAPIHSIMRKVISDIPVPNTLSSPKQHENATYVIPKGHFVLASPGVAQMD

PLIWQNAEEWNPHRWDDESGVAAMAAEQYTEGDKVDYGYGSVSKGTESPYQPFGAGRHRC

IGESFAYVQLQTILATLCRRVVLKLEGPFPAPNYQTMIVLPLKGQTKIMYKLRA*

>CYP51F(CNAG_00040)Cryptococcus neoformans var. grubii H99

MSAIIPQVQQLLGQVAQFIPPWFAALPTSVKVVIAVIGIPALVICLNVFQQLCLPRRKDL

PPVVFHYIPWFGSAAYYGEDPYKFLFECRDKYGDLFTFILMGRRVTVALGPKGNNLSLGG

KISQVSAEEAYTHLTTPVFGKGVVYDCPNEMLMQQKKFIKSGLTTESLQSYPPMITSECE

DFFTKEVGISPQKPSATLDLLKSMSELIILTASRTLQGKEVRESLNGQFAKYYEDLDGGF

TPLNFMFPNLPLPSYKRRDEAQKAMSDFYLKIMENRRKGESDHEHDMIENLQSCKYRNGV

PLSDRDIAHIMIALLMAGQHTSSATSSWTLLHLADRPDVVEALYQEQKQKLGNPDGTFRD

YKYEDLKELPIMDSIIRETLRMHAPIHSIYRKVLSDIPVPPSLSAPSENGQYIIPKGHYI

MAAPGVSQMDPRIWQDAKVWNPARWHDEKGFAAAAMAQYSKAEQVDYGFGSVSKGTESPY

QPFGAGRHRCVGEQFAYTQLSTIFTYVVRNFTLKLAVPKFPETNYRTMIVQPNNPLVTFT

LRNAEVK

>CYP51F1(KGB74675.1)Cryptococcus gattii VGII R265

MSAIIPQVQQLLGQVAQYIPHWFTALPTSLKIVIAVIGIPAFIIGLNVFHQLCLPRRRDL

PPVVFHYIPWFGSAAYYGEDPYKFLFECRDKYGDLFTFILMGRRITVALGPKGNNLSLGG

KISQVSAEEAYTHLTTPVFGKGVVYDCPNEMLMQQKKFIKSGLTTESLQSYPPMITSECE

DFFTKEVGISSQKPSITLDLLKSMSELIILTASRTLQGKEVRESLNGQFAKYYEDLDGGF

TPLNFMFPNLPLPSYRRRDEAQKAMSDFYLKIMENRRKGESDHEHDMIENLQGCKYRNGV

PLSDRDVAHIMIALLMAGQHTSSATSSWTLLHLADRPDVVEALYQEQKEKLGNPDGTFRD

YNSSIYRKVLSDIPVPPSLAAPSENGQYIIPKGHYIMAAPGVSQMDPRIWQDAKVWNPAR

WHDEKGFAAAAMAQYTKAEQVDYGFGSVSKGTESPYQPFGAGRHRCVGEQFAYTQLSTIF

TYVVRNFTLKLAVPKFPETNYRTMIVQPNNPLVTFTLRNAEVKQEV

>CYP51F(KIY58480.1)Cryptococcus gattii VGII 99/473

MSAIIPQVQQLLGQVAQYIPHWFTALPTSLKIVIAVIGIPAFIIGLNVFHQLCLPRRRDL

PPVVFHYIPWFGSAAYYGEDPYKFLFECRDKYGDLFTFILMGRRITVALGPKGNNLSLGG

KISQVSAEEAYTHLTTPVFGKGVVYDCPNEMLMQQKKFIKSGLTTESLQSYPPMITSECE

DFFTKEVGISSQKPSITLDLLKSMSELIILTASRTLQGKEVRESLNGQFAKYYEDLDGGF

TPLNFMFPNLPLPSYRRRDEAQKAMSDFYLKIMENRRKGESDHEHDMIENLQGCKYRNGV

PLSDRDVAHIMIALLMAGQHTSSATSSWTLLHLADRPDVVEALYQEQKEKLGNPDGTFRD

YNSSIYRKVLSDIPVPPSLAAPSENGQYIIPKGHYIMAAPGVSQMDPRIWQDAKVWNPAR

WHDEKGFAAAAMAQYTKAEQVDYGFGSVSKGTESPYQPFGAGRHRCVGEQFAYTQLSTIF

TYVVRNFTLKLAVPKFPETNYRTMIVQPNNPLVTFTLRNAEVKQEV

>CYP51F(KIR93986.1)Cryptococcus gattii VGII CBS 10090

MSAIIPQVQQLLGQVAQYIPHWFTALPTSLKIVIAVIGIPAFIIGLNVFHQLCLPRRRDL

PPVVFHYIPWFGSAAYYGEDPYKFLFECRDKYGDLFTFILMGRRITVALGPKGNNLSLGG

KISQVSAEEAYTHLTTPVFGKGVVYDCPNEMLMQQKKFIKSGLTTESLQSYPPMITSECE

DFFTKEVGISSQKPSITLDLLKSMSELIILTASRTLQGKEVRESLNGQFAKYYEDLDGGF

TPLNFMFPNLPLPSYRRRDEAQKAMSDFYLKIMENRRKGESDHEHDMIENLQGCKYRNGV

PLSDRDVAHIMIALLMAGQHTSSATSSWTLLHLADRPDVVEALYQEQKEKLGNPDGTFRD

YNSSIYRKVLSDIPVPPSLAAPSENGQYIIPKGHYIMAAPGVSQMDPRIWQDAKVWNPAR

WHDEKGFAAAAMAQYTKAEQVDYGFGSVSKGTESPYQPFGAGRHRCVGEQFAYTQLSTIF

TYVVRNFTLKLAVPKFPETNYRTMIVQPNNPLVTFTLRNAEVKQEV

>CYP51F(KIS00993.1)Cryptococcus gattii VGII 2001/935-1

MSAIIPQVQQLLGQVAQYIPHWFTALPTSLKIVIAVIGIPAFIIGLNVFNQLCLPRRRDL

PPVVFHYIPWFGSAAYYGEDPYKFLFECRDKYGDLFTFILMGRRITVALGPKGNNLSLGG

KISQVSAEEAYTHLTTPVFGKGVVYDCPNEMLMQQKKFIKSGLTTESLQSYPPMITSECE

DFFTKEVGISSQKPSITLDLLKSMSELIILTASRTLQGKEVRESLNGQFAKYYEDLDGGF

TPLNFMFPNLPLPSYRRRDEAQKAMSDFYLKIMENRRKGESDHEHDMIENLQGCKYRNGV

PLSDRDVAHIMIALLMAGQHTSSATSSWTLLHLADRPDVVEALYQEQKEKLGNPDGTFRD

YNSSIYRKVLSDIPVPPSLAAPSENGQYIIPKGHYIMAAPGVSQMDPRIWQDAKVWNPAR

WHDEKGFAAAAMAQYTKAEQVDYGFGSVSKGTESPYQPFGAGRHRCVGEQFAYTQLSTIF

TYVVRNFTLKLAVPKFPETNYRTMIVQPNNPLVTFTLRNAEVKQEV

>CYP51F(KIR83059.1)Cryptococcus gattii VGIV IND107

MSAIIPQVQQLLGQVAQYIPPWFTALPTSLKIVIAVIGIPAFIIGLNVFRQLCLPRRKDL

PPVVFHYIPWFGSAAYYGEDPYKFLFECRDKYGDLFTFILMGRRITVALGPKGNNLSLGG

KISQVSAEEAYTHLTTPVFGKGVVYDCPNEMLMQQKKFIKSGLTTESLQSYPPIITSECE

DFFTKDVGISSQKPSATLDLLKSMSELIILTASRTLQGKEVRESLNGQFAKYYEDLDGGF

TPLNFMFPNLPLPSYRRRDEAQKAMSDFYLKIMENRRKGESDHEHDMIENLQGCKYRNGV

PLSDRDVAHIMIALLMAGQHTSSATSSWTLLHLADRPDVVEALYQEQKEKLGNPDGTFRD

YKYEDLKELPIMDSIIRETLRMHAPIHSIYRKVLSDISVPPSLAAPSENGQYIIPKGHYI

MAAPGVSQMDPRIWQDAKVWNPARWHDEKGFAAAAMAQYTKAEQVDYGFGSVSKGTESPY

QPFGAGRHRCVGEQFAYTQLSTIFTYVVRNFTLKLAVPKFPETNYRTMIVQPNNPLVTFT

LRNAEVKEEV

>CYP51F(KJE05898.1)Cryptococcus gattii NT-10

MSAIIPQVQQLLGQLAQYIPPWFTALPTSLKIVIAVIGIPAFIIGLNVFQQLCLPRTKDL

PPVVFHYIPWFGSAAYYGEDPYKFLFECRDKYGDLFTFILMGRRITVALGPKGNNLSLGG

KISQVSAEEAYTHLTTPVFGKGVVYDCPNEMLMQQKKFIKSGLTTESLQSYPPMITSECE

DFFTKEVGISQKPSATLDLLKSMSELIILTASRTLQGKEVRESLNGQFAKYYEDLDGGFT

PLNFMFPNLPLPSYRRRDEAQKAMSDFYLKIMENRRKGESDHEHDMIENLQGCKYRNGVP

LSDRDVAHIMIALLMAGQHTSSATSSWTLLHLADRPDIVEALYQEQKEKLGNPDGTFRDY

KYEDLKELPIMDSIIRETLRMHAPIHSIYRKVLSDIPVPPSLAAPSENGQYIIPKGHYIM

AAPGVSQMDPRIWQDAKVWNPARWHDEKGFAAAAMAQYTKAEQVDYGFGSVSKGTESPYQ

PFGAGRHRCVGEQFAYTQLSTIFTYVVRNFTLKLAVPKFPETNYRTMIVQPTNPLVTFTL

RNAEVKQEV

>CYP51F(KIY36451.1)Cryptococcus gattii E566

MSAIIPQVQQLLGQLAQYIPPWFTALPTSLKIVIAVIGIPAFIIGLNVFQQLCLPRTKDL

PPVVFHYIPWFGSAAYYGEDPYKFLFECRDKYGDLFTFILMGRRITVALGPKGNNLSLGG

KISQVSAEEAYTHLTTPVFGKGVVYDCPNEMLMQQKKFIKSGLTTESLQSYPPMITSECE

DFFTKEVGISSQKPSATLDLLKSMSELIILTASRTLQGKEVRESLNGQFAKYYEDLDGGF

TPLNFMFPNLPLPSYRRRDEAQKAMSDFYLKIMENRRKGESDHEHDMIENLQGCKYRNGV

PLSDRDVAHIMIALLMAGQHTSSATSSWTLLHLADRPDIVEALYQEQKEKLGNPDGTFRD

YKYEDLKELPIMDSIIRETLRMHAPIHSIYRKVLSDIPVPPSLAAPSENGQYIIPKGHYI

MAAPGVSQMDPRIWQDAKVWNPARWHDEKGFAAAAMAQYTKAEQVDYGFGSVSKGTESPY

QPFGAGRHRCVGEQFAYTQLSTIFTYVVRNFTLKLAVPKFPETNYRTMIVQPTNPLVTFT

LRNAEVKQEV

>CYP51F(KIR77383.1)Cryptococcus gattii EJB2

MSAIIPQVQQLLGQLAQYIPPWFTALPTSLKIVIAVIGIPAFIIGLNVFQQLCLPRTKDL

PPVVFHYIPWFGSAAYYGEDPYKFLFECRDKYGDLFTFILMGRRITVALGPKGNNLSLGG

KISQVSAEEAYTHLTTPVFGKGVVYDCPNEMLMQQKKFIKSGLTTESLQSYPPMITSECE

DFFTKEVGISSQKPSATLDLLKSMSELIILTASRTLQGKEVRESLNGQFAKYYEDLDGGF

TPLNFMFPNLPLPSYRRRDEAQKAMSDFYLKIMENRRKGESDHEHDMIENLQGCKYRNGV

PLSDRDVAHIMIALLMAGQHTSSATSSWTLLHLADRPDIVEALYQEQKEKLGNPDGTFRD

YKYEDLKELPIMDSIIRETLRMHAPIHSIYRKVLSDIPVPPSLAAPSENGQYIIPKGHYI

MAAPGVSQMDPRIWQDAKVWNPARWHDEKGFAAAAMAQYTKAEQVDYGFGSVSKGTESPY

QPFGAGRHRCVGEQFAYTQLSTIFTYVVRNFTLKLAVPKFPETNYRTMIVQPTNPLVTFT

LRNAEVKQEV

>CYP55A28(815884)Cryptococcus terricola

MATFTLKSRPALHLSSATKWTRTLASAVTPPKFPFSRPSGVLPPVEYAQLRAQEPVSKVE

LWDGSHPWLVVKHKDVCSVLTDERLSKIRTRPGFPEMSAGGKAAAGNKPTFVDMDPPEHM

QQRSMVEPLFTREHIEKMRPDIQTTVNSLLGTMVDAGCKNPVDLVEKFALPVPSHIIYGI

LGTPLEDLAYLTSCNAIRTSGSSTASQASSASNELLQYLGKLVDAKIKSPGKDLISKLVV

EQLKPGHLAREDVVQIAFLMLVAGNATMVNMINLGVVTLLDHPKQLMELKKDPSLAKSFV

EELCRFHTASSFATRRVAKEDITLGGKTIKAGEGIIAATQSANRDESIFPNPDTFDMHRA

AGPALGFGYGAHRCVAEWLARAELEIVFSTLFQKLPNLKLAVPASELQYTPPTMDVGISA

LPVVW*

>CYP505W1(327997) Naganishia vishniacii v1.0

MVTARPIPGPTPKPIIGNLSDVDAELGALSIAHLIQRYRELIQLNFLGNRRLFAGSRRIV

HELCDQSRFEKKVTGSLEQARNLAGDAHQNEPSWEIAHRILIPAFGLLSIREMYPGMLDI

AEQLISKWHRFGDAKIDVVSDFTKLTLDIISNEVISKGDVQMKRHIVLEIPPEATYRAGD

YLGLPPTTPLPVVMRALVRQTCMWTTPLHSKEPAQEGPCLLVDHTALSACSRNNLEQPAT

VKQIKTLAELSEVSQDKRVSLLALLEEFQGLPISVAEFVEMLLSIKMRQYSISSSPLDNP

HRVTLTFSVLDAPHLSGRGQRYYGTATHFLSKLQPGVKIHAAVRPSNEGFHPPADPKVPM

IMGCAGTGITPFRSFIQERALQKAAGREVGPTLLFYGCHSADTDLLYDTEMAQWQQQGVV

QVRHAFSKGANQSEGCRHVQDRVWHDREEVAALFKNGARIYV*

>CYP505W(132718)(Cryptococcus werringae)

MSFIPEQELKPIPQPPPQFIIGNLRDIDPENAVGSICNMFSRYGEVVKLQLPAAPKEGRI

FIGSQRLVHEVCDQERFEKIVTGGLEQARNVAGDGLFTAHGSEESWAIAHRILVPAFGPL

SIRNMFPGMVDIATQLIEKWTRFGDAKIDVVADFTRLTLDTIALCTFSHRFNSFYTEGSP

PFVEAMASSLHESGARNRRIPGTNWMYTKANQQYKDDIALQHGIADELIAERIKDMDSAP

DDLLSTMLKGRDPKTGQGLSPENIRFQLVTFLIAGHETTSGMLSFLFHFLMKNPRAYHEI

REEMGRVCGNNPVKYEHLQKLKYIDAALKETLRLKSTAPGWTVAAKKDEVLAGKYRVYKG

QSILVVLDALHRDPAVWGDDVEDFRPERMLDGKFEALPPDSWKPFGNGMRGCIGRPFAWQ

EALLVVAMVIQHFDCRPDDESYQLAIKTSLTIKPSGFFMHAKKRHNSPQLGLAQSINKVV

AGTDAQETDAEVDSNPDGPRLGIYYGSNAGTCKALAYKMKTEAKSRGLNVSIDHLNSLSS

QSVPRDRPIAIVTASYEGQPTDDANFFVEWLKTAEDKSQAGVKYAVFGCGHPDWVATFHA

IPTLIDRRLAECGAERLCKRGEGNAAAAELFDQFDDWEAEFLDTIGTSASTSSEPTLTAE

IDRDIRKNLLQQEFMRSITILANDVISKGDVEIKRRIRLELPKEMQYRAGDYIGLLPVNL

PVVQRALIRFALHADDVITLKGSGGAYTLPMNMPISAFDLLGGFVELEQPATLKQVERLK

QYVEEESPDSQTLKHYCEANVYEKELAAKRVDLLSLLEELTDVEMPFTEFLASLPSIRMR

QYSISSSPLNVPNQAELTIAVVDAPHISGRKNRYYGIATHYLSNLLPGSRMRAGVKPSNE

QFHPPTDPSISMLMAASGSGIAPFRGFIQERALQKAAGRDVGTAMLFYGCRSSDTDLLYD

DEMQEWERQGVVKVYHAFSRDSDRTEGCKYVQDRIYHERKTVVEAFKTGARVYLCGSSRM

ATGVKEICAQIYADEKGVGLEKGKEWLSTVQAARFATDIFG*

>CYP5702A(375039)Cryptococcus curvatus

MDSKHVNVKLPAVIQDLPAWAPFAAVAGCVLWYYLYPYFVTYGALRDIPAPFPAQFTNWW

LFLVVRRGDRYATADREHKRLGKMIRLAPNHVSIADDSAIKAIYGHGNGFLKSDFYDAFV

SIRRGLFNTRDRAEHTRKRKVVSHTFSPKSVREFEPYMQENLHLFVKQWDNLIAKEKMNP

KSNGQPKVDCLDWFNFLAFDMIGDLAFGAPFGMLEAGADIAELRSTPDAPATFAPAIEIL

NRRGEISATLGVFPQIRPYAKYLPDPFFSKGIEAVQNLAKIAIARVKQRLDNPPADARND

LLSRLQEGRDHKGQPIDREELTAEALTQLIAGSDTTSNSSCALLYYATKTPGVLAKLQRE

LDEAIPEGTDVPTFDMIKSLPYLEAVFNETLRYHSTSGIGLPREIPPGSPGVTICGHYFP

AGTVLSVPTYTIHHSKEIWGPDADEFNPERWFNLTARQKEAFIPFSHGPRSCVGRNVAEM

EMKLIAATWGNRYHPTLLQDVMETREGFLRKPLALNIEFKQRRRAVV*

>CYP5702A(579701)Cryptococcus werringae

MVTTSLLLASVILIAVLAILVSPNRKLDKYPGPLAAKFTRLWLARQAGKGNRYEVVHQLH

KTHGKFVKLAPDHVSIASPAAIPIVYGHGTGFIKADFYDAFVSIRRGLFNTRDRKEHTRK

RKIVSHVFSQKNVLNFEQHIHTVIHNLFDKWDTMAKDDVQLAKGFDILDCKSYPAVRYHG

RQPANPPPYPTGLNFFAFDVIGSLAFGRPFGMVSAGKDMAPVASPTTGQIESLPAITILN

RRGEFSATMGCIPPILRPLAKKLPWFRMGLESVRRLAGIAIAAVAVRLEAEESGMMDDRR

DLLSELIKGKDENGLPMGKEELTAEALTQLIAGSDTTSNSSCAILYYLSTSPRIMAKLVD

SLAPIAREKPSDFHSFEYGEVKDLTYLQACIDEALRLHSTSALGLPRIVPVGGAVVCDEF

FPEGTTLSVPAYTVHRDPTVWGEDADTYRPERWFERDKSTMNASFIPFSFGPRSCVGRNL

ASMELLLIISSTVWRYEITPMRPGERLNTVEGFLRKPTGFQARIGRRDHKRELE*

>CYP5702A(806727)Cryptococcus terricola

MSSVIDQILVHAKLPLIAFGVTVGLILLNVVRIYLVDPYGLRSYPGPKAAALSELWLARA

AGRGNRYQVVHNEHKKHGKFVRLAPTHLSIADPAAIQIVYGHGTGFLKSDFYDCFVSIRR

GLFNTRDRTEHTRKRKIVSHVFSQKNVLGFEKHIHDALKELVKQWDNMCYQAGKVGDNKE

VAEFDVLNWLNFFAFDVIGSLAFGRPFGMLVSGKDIAPVKVEGSDKTEYLSAIQILNDRG

EYSATMGVMRPWMRPIAKKFPWFRRGDDAVKKLAGIAIAAVTQRLENPSDRDDLLSKLQA

GKDENGNPLGREELTAEALTQLIAGSDTTSNSSCAILYHLSNNPRVMKKLQAELDKVAEE

RREDDVHFLYEEVKNVEYLQACIDEALRIHSTSSLGLPRDVPAGGAEVCGRWFPEGTTLS

VPAYTIHRDKAIWGEDSEDYRPERWFEQDKEAMAKAYVPFSFGPRSCVGRNLASMELLLL

ISTVVYRYDVTPLHPGEPLKTIEGFLRKPTEFDIKLKRRDIE*

>CYP5702A1(295604) Naganishia vishniacii v1.0

MTNSVVLQQVSDLIHRLSDPIVGIPVVIAVALLSILIPFILDPNGFRKYPGPLPAALSKL

WLARQAGNGNRYEIVHDQHQKFGKFVRIAPDHVSIADHNAIHEIYGHGNGFLKTEFYDAF

VSIRRGLFNTRDRAEHTRKRKIVSHVYSMKNVNSFEPHIAAAMVELFKQWDAMCEKAAKY

PGLDGNSAKGLAVFDILNWFNFFAFDVISTLCFGKSFGMLASGKDIAPVKLEGSNETVYL

PAIQILNDRGEYSATLGVLPMWMRPWAKKLPWFSRGVKSVKNLAGIAIAAVGDRLENPSD

REDLLAKLQAGKDETGKPMGREELTAEALTQLIAGSDTTSNSSCAIVYYLSNNPRVMKKL

REELDKAAEEKAGGDEVLHFDYEEVKSLPYLQACIDEALRLHSTSAIGLPREVPAGGATV

LGEFFPGGTTVSVPAYTIHRDPSVWGEDSEAYRPERWFERDPKLLQSSFIPFSYGPRSCV

GRNLASMELSLLISSVIHRYDIEPLVSGKKLPTAEGFLRKPLEYNVAVKRRAV*

>CYP5701B1(816264)Cryptococcus terricola

MDKVTQTIQGRTWDLMVPGMGRAWVVEDPACVKHVLSTRHDAFGKGEFAGSRMRELFGNG

IVIATGSSWKLQRLAGNQAFNRPQPLKIFAEKILGKHINVLLRGFEEVCVEVDAKSNSKG

KGNIVDVMDVAEEYAMAVFGELAYDSNFSRISSSFSIPFSRASDITSARFFNPFYRFTER

VTPRGRKFHNDVKEVKKFGRELVREMIGKVEDMEIGETPEEDGDTRGLLLKELIKAHGGE

SGDEEFLADACMNFLTAGKDTTAQALAWSIYELLIHPTWIPLIRREINLHPSADSNISYQ

DLTSQSGPFRCTNAFISEVLRLRPPVPIEILQNTLNETVLLPDQTVVKPGENVLWSPWIM

GRSIRIWGDDARTFRPERWMDEETGEKGTRHPNQKTAYEFPVFHGGPRSCLGKPLARAEL

AYSLLAIVGRYDLEPAWDTTKEKGMGGGLLSPVDGGVPVRVRRRRRFRV*

>CYP5701C1(633526)Cryptococcus werringae

MSTLLIPIALILPLLYWIYHHCARTYTPLPLIGHLPWFALQSRQGSLRWISNGSDRFEGR

GWKLRLPLIGQAWVVEDPACLKHILSTKHDHFGKGQFATSRMKDLFGEGIILTQTHWSTQ

RQAGSRSFLAPANLRAFVHRILPRHLEGVFELMDPRAGGDVDVDGKGKDDGEKKVDLQEV

AYVYAMAVFGELAYHASPFPIPPSTLSRINCKLISDPLEFDGQSNFPRISEKFTKPFDRS

AILIHSRFFNPIWRWTERLTISGWKLRRDLRQLRRVGLDLVRAGKEGLRREEEGLEGDEK

GDDGSGEGKGSGLLLRELVRENGAVRDETFLADSCLNFLTAGRDTTAQALSWTVFLLLTH

PSTLERVTEELRDSPSPETYLASHALGDLTSSNDDSYCPFSNAVITESLRLHPPVPLEVH

ENTGTQPILLPDRTIVEPGEKVVWSPWAMARSTRIWGSDAASFKPERWLPFTAPEGERKP

GLRRTAFEFPVFHAGPRSCLGKQLARIELIFILREVVRRYELTPDWDVAVDRVVGQGLTG

PIEGGLPVRVRRRTA*

>CYP5701A1(278090) Naganishia vishniacii v1.0

MFWLLYLAGIALIGLYIIRARIRNFQGLPLVGHLPFFALQSRQNLLDWIYRGTEHSQGHS

WRIDVPFIGRGWIIENPACLKDVLSSKHDSFGKGQLASTRMKDLFGEWIILVEGHKWKAQ

RLAGHHAFSSNANLQTFCETILPKHLDTLLEPFERAIERDEELDVQDVTYDFAMVVFGEL

AYNSDFARASASFTLPFERASTVTQARFTNPMYRIMERVLPYGRRFKDDVAYVKAFGERI

VRSMTERLKEEEEGKMDLPEPEEAKGAGSTVPSPGLLLRELVREYGKDDDYGFLADACLN

FLTAGKDTTAQALAWTIYAILREPMILANLQVEIDKAPVDASIADLTATDGPLQYMNAVI

SESLRLHPPVPLEIHENISNQAIALADGSIVEPGEIVLWSPWAMGRSTRLWGADAADFKP

TRWFPEVKPHASYDSNISHPLKKTAFEFPVFHAGPRSCLGKTLAKIELSYALMQLLRRYD

MVPTWPLNREKSVGTALTGPVLGGLKVRARRRERL*

>CYP5704C1(576315)Cryptococcus werringae

MIISNLISTTTSLGLFPTLIALYLSWLVYKFISAYKLAASINHHPGYRGLFGVRTLIGRF

LPSHLPYITESINSVWHSKRLPYTRANTSILSDISLFGNHVYLNVATPGAVRVLNAERKT

FRKPTEVYKLLKGFGENLVVTEGEEWRRHRKIAAPGFNEEVFDLVSRTTTEIFHLMFHSN

HWQTLSPGDHAVVSDVNNMLLKMALTVLATASFGAPLTWSDSSSSLPDPSQNVNANAAGT

GTAGMTEREREEKRELMGKGHELTFVQAITGVSENIFLMILLPKWLRWLPFKKVKETNLA

QKELNAYMYGMIRTRKEEIKRGEKGGQGRKDLFNQLIEASLREEGGKGLTDEELVGNVFI

FALAGHETSAHSLSFTFAYLATHPEIQEWLFEEVAAVFPDGHLPSYKDFPALPRTLAVLY

ETLRLHPSVNVIPKYATEDTVLMDDPLAWFTEEPPVGGWLGGKVDGDGVGERRKVFVPQN

AQVQIDVVNLHRHPVYWPEPDTFKPERFLGEYNRDAFVPFSAGARGCIGRRFAEVEAVTF

LALIVRHFQLVPNPLTVPPGETKGETMERLLSAKEGLTLTPKGIDLRFVRREGMVGREEF

FV*

>CYP5704B1(814409)Cryptococcus terricola

MSTITILTAIVVVAGLFIRRCVAFYQQIQATHGQSGFRTLLSPQTLLGNLVPANDTLNPQ

LTFGWRTKRFFYDKFRSSIITLVAFNSPETIYSVSNPEAVRILTTDRRRWAKPVESYRIL

ALYGDNVLTTEGEKWRKHRKIVAPGFSEKVFDLVWRTSTTVVHEMMDAEGFSALKPNERT

DIKDITHLTIRLALAVLATASFGSKLKWSTGTQKYIPSGHTMTMADAITTSEKIIWLLLA

PRALFKLPIAYLQHIKASSDELLSFMTDMIRSRRSELAVGAANQRYDLFNQLIENSGADI

AAGEKGEGGLKDDELVGNIFIFALAGHETSAHSLAFAFAYLALYPKKQQWLREEVMQVMP

DGHAPSYKDFPKLERCLAVLYETLRLHPAVTIIPKVALEDTYLPDDTYPLPLLKESESEI

PTPFGQRRAFIPKGSHVHIDTTALHLHPSHWADPAAFKPERFLEDYNKDAFLPFSAGARA

CIGRRFAEVESVCILALMIRTFDITPIPSNESFEQKRERLLAAVNKLTLTPKNPLPLRLI

KRDVRDLDWDASS*

>CYP5704A1(327024) Naganishia vishniacii v1.0

MGSTISLASRPNLWLTAAFLLASWITYRFVLFRKSIKRINSHPGFRSFLSDMNIIAYLLP

KGIPYVNAHIGWQWRQKRELYRKSGTSILSTVSFIDNRTTYSVSSPEACRILNSDRRKFQ

KPVKLYEILKIVAPGFSEKVFELVWNTSSWITFELFKSEGWEALQPGQSMDVPNIPELTL

RVTLAVLSTASFGAPLSWKQDPKESRGVGHDMSFVEAISEVSENIFWLLAMPKFVYKLPI

RKIQRVGLADRELRSYMHQMIQARREEIRHGESSAKDDLFDALVTASMQEESEGKTGSGG

LTDDELVGNTFITALAGHETSSHTLAFAIAYLALHPEKQAWLFDELIQVYPPSHVPTYAD

FPHLVRTLAVIYEALRLHPAVVYIPKYATEDTWLPDDPEDGADGEEPLRVFVPKGTECGI

DTVGLQRHPRYWKNPNAFEPERFLEDYNRDAFAPFSAGARACIGRKFAEVEMVCVLSLLV

RTYQILPHDTPDPSVVAPLPSSHEPTLPDLLTEKLLETKPGVTLTPRDIGIRLVKRREGS

VNRPEVEL*

>CYP5215A(2740581118)Cryptococcus amylolentus CBS 6273

MGIPIRPLTVCESLGLALLAFVVWAVHIFLYKPLTSPLRHLPCPEGGWAVGGHIDHILDL

GSEVPNQWIDELGPTFMVRGFFAVHHRIFTVDPRALDHVLQNTDVYTKPDILRSLVKRYM

KNGLIVAEGTRHRIQRKVTQKLFSTVSTRAMGVLVQDKSNQLREILRHLCASPATSTPFS

QANPTLPAVTRQVDVYSSASRCMFDVIGAVAVDSVNPPFNAAGDYTSFGAHLFHKYEKMQ

LLCEGAMGLRMFFSLYFPWIDSIWPSENTKRVNDGMGSLLDFAKHQMAERRAELEAMNGK

KGDVAERRDLLTLMLKHNMMKNLSPTDKLRDDEISGQLSTFMFAGSETTAGTISFGLYDL

ARHSEIQAKLRAEILECAETFTSEQLDELPYLDAVVKEIMRCNPSLPGTVRMATRDDMIP

LAVPVKLSDERVVSEIRIRKGQLIHVPIDHLHACTHIWGPTANEFDPSRFLDAPSTYPSS

TPTTPSFPPDAGPVLSSRRTSVPSIVPAGPGIWPNFMTFIDGPRRCIGYKLAVMEIKMVL

FTLLREFEVEPVKGIRIGRWNMMSNRPYVSGTLMSEGSRLPLNFRLYKGENENVAQDAKL

>CYP5215A3(KGB79607.1)Cryptococcus gattii VGII R265

MMPEQLSLHLPTTIESMFALTVFALLWLLRVFVYKPFKSPLKEVSCPPGGTGSQGHIAEI

LDLQGTKVHDWIKAYGTTFIVRGPFGVHHRIFSIDPRALNHVLKHTNIYTKSDLLRDLVR

RYMEGGLIVGEGEGEGHKVQRKVSQKLFSMGGPKSMGQVVQDKSNQRDILPNLCEDPTVS

NPPPVNPTLSTGSREVDVYSTASRCIFDLVGSVGVDHQFGSLGNWEGSGGKLFQKYERMQ

LLCPGAMGFRMLLSLTWPLIDKIWPSENIKRVNDAMGSLQKFAKEMMTERRLELSIIGSK

KGDVPDRKGLLTLMLRHNMAQKINAADSLQDHEISGQLSTFLFAGSETTAGTISFGLYDL

TCHPDIQSRLRTEILECGDNLPFDQIDELPYLDAVVKEIMRINPSLPGTVRQAQKDDIIP

LAKPVTLTNGKVVTDIHIRKGQLVHVPIEHLHTLEHIWGPAAEFDPSRFFSSPQSSAFSQ

PTLGSHSAATSSARRDAVPSYVPEGPGIWPNFMTFIDGPRRCIGYKLAVMEIRTIIFTLV

GDFEIELMKGQHILRWNMMSNRPFVANTSWSKGSRLPLHFKLYKGGGQYEESVEFVSS*

>CYP5215A(CGB_A0660W)Cryptococcus gattii WM276

MFSMMLEQLPSLQLLTTTGSIFALIWLLHVFVYKPFTSPLKNVPCPPGGTGSQGHIAEIM

DLQGTKIYDWIKAYGSTFMVRGPFGVHHRIFSVDPRVLNHVLKHTNIYTKSDLLRDLVRR

YMKEGLIVAEGERHKVQRKVSQKLFSMGGLKSMGQVVQDKSNQLRDILLNLCANLTASNP

YSPVNPTLSPGSREVDVYSTVSRCTFDLIGSIGVGHQFDSLGNWEGSGGKLFQKYERMQL

LCPGAMGFRMLLSLTWPLIDKIWPSENTKRVNDAMGSLQKFAKEKMIERQLELATVDSKK

GDIPDRKDLLILMLRHNLAQKINAADKLRDHEIFGQLSTFLFAGSETTAGTISFGLYDLA

YHPDIQSRLRAEILECGDNLSFDQIDELPYLDAVVKEIMRINPSLPGTVRQAQKDDIIPL

AKPVTLTNGKVVADIHIRKGQLVSSFLFLMQSLFRPLKG

>CYP5215A(KIS00893.1)Cryptococcus gattii VGII 2001/935-1

MFALTVFALLWLLRVFVYKPFKSPLKEVSCPPGGTGSQGHIAEILDLQGTKVHDWIKAYG

TTFIVRGPFGVHHRIFSIDPRALNHVLKHTNIYTKSDLLRDLVRRYMEGGLIVGEASRYP

SQSFREVDVYSTASRCIFDLVGSVGVDHQFGSLGNWEGSGGKLFQKYERMQLLCPGAMGF

RMLLSLTWPLIDKIWPSENIKRVNDAMGSLQKFAKEMMTERRLELSIIGSKKGDVPDRKG

LLTLMLRHNMAQKINAADSLQDHEISGQLSTFLFAGSETTAGTISFGLYDLTCHPDIQSR

LRTEILECGDNLPFDQIDELPYLDAVVKEIMRINPSLPGTVRQAQKDDIIPLAKPVTLTN

GKVVTDIHIRKGQLVHVPIEHLHTLEHIWGPAA

>CYP5215A(KIR94582.1)Cryptococcus gattii VGII CBS 10090

MFALTVFALLWLLRVFVYKPFKSPLKEVSCPPGGTGSQGHIAEILDLQGTKVHDWIKAYG

TTFIVRGPFGVHHRIFSIDPRALNHVLKHTNIYTKSDLLRDLVRRYMEGGLIVGEASRYP

SQSFREVDVYSTASRCIFDLVGSVGVDHQFGSLGNWEGSGGKLFQKYERMQLLCPGAMGF

RMLLSLTWPLIDKIWPSENIKRVNDAMGSLQKFAKEMMTERRLELSIIGSKKGDVPDRKG

LLTLMLRHNMAQKINAADSLQDHEISGQLSTFLFAGSETTAGTISFGLYDLTCHPDIQSR

LRTEILECGDNLPFDQIDELPYLDAVVKEIMRINPSLPGTVRQAQKDDIIPLAKPVTLTN

GKVVTDIHIRKGQLVHVPIEHLHTLEHIWGPAA

>CYP5215A(KIY57602.1)Cryptococcus gattii VGII 99/473

MFALTVFALLWLLRVFVYKPFKSPLKEVSCPPGGTGSQGHIAEILDLQGIKVHDWIKAYG

TTFIVRGPFGVHHRIFSIDPRALNHVLKHTNIYTKSDLLRDLVRRYMEGGLIVGEASRYP

SQSFREVDVYSTASRCIFDLVGSVGVDHQFGSLGNWEGSGGKLFQKYERMQLLCPGAMGF

RMLLSLTWPLIDKIWPSENIKRVNDAMGSLQKFAKEMMTERRLELSIIGSKKGDVPDRKG

LLTLMLRHNMAQKINAADSLQDHEISGQLSTFLFAGSETTAGTISFGLYDLTCHPDIQSR

LRTEILECGDNLPFDQIDELPYLDAVVKEIMRINPSLPGTVRQAQKDDIIPLAKPVTLTN

GKVVTDIHIRKGQLVHVPIEHLHTLEHIWGPAA

>CYP5215A(KJE05209.1)Cryptococcus gattii NT-10

MFSMMLEQLPSLQLLTTTGSIFALIWLLHVFVYKPFTSPLKNVPCPPGGTGSQGHIAEIM

DLQGTKIYDWIKAYGSTFMVRGPFGVHHRIFSVDPRVLNHVLKHTNIYTKSDLLRDLVRR

YMKEGLIVAEGERHKVQRKVSQKLFSMGGLKSMGQVVQDKSNQLRDILLNLCANLTASNP

YSPVNPTLSPGSREVDVYSTVSRCTFDLIGSIGVGHQFDSLGNWEGSGGKLFQKYERMQL

LCPGAMGFRMLLSLTWPLIDKIWPSENTKRVNDAMGSLQKFAKEKMIERQLELATVDSKK

GDIPDRKDLLILMLRHNLAQKINAADKLRDHEIFGQLSTFLFAGSETTAGTISFGLYDLA

YHPDIQSRLRAEILECGDNLSFDQIDELPYLDAVVKEIMRINPSLPGTVRQAQKDDIIPL

AKPVTLTNGKVVADIHIRKGQLVSSFLFLMQSLFRPLKG

>CYP5215A(KIR79926.1)Cryptococcus gattii EJB2

MFSMMLEQLPSLQLLTTTGSIFALIWLLHVFVYKPFTSPLKNVPCPPGGTGSQGHIAEIM

DLQGTKIYDWIKAYGSTFMVRGPFGVHHRIFSVDPRVLNHVLKHTNIYTKSDLLRDLVRR

YMKEGLIVAEGERHKVQRKVSQKLFSMGGLKSMGQVVQDKSNQLRDILLNLCANLTASNP

YSPVNPTLSPGSREVDVYSTVSRCTFDLIGSIGVGHQFDSLGNWEGSGGKLFQKYERMQL

LCPGAMGFRMLLSLTWPLIDKIWPSENTKRVNDAMGSLQKFAKEKMIERQLELATVDSKK

GDIPDRKDLLILMLRHNLAQKINAADKLRDHEIFGQLSTFLFAGSETTAGTISFGLYDLA

YHPDIQSRLRAEILECGDNLSFDQIDELPYLDAVVKEIMRINPSLPGTVRQAQKDDIIPL

AKPVTLTNGKVVADIHIRKGQLVSSFLFLMQSLFRPLKG

>CYP5215A(KIY34432.1)Cryptococcus gattii E566

MFSMMLEQLPSLQLLTTTGSIFALIWLLHVFVYKPFTSPLKNVPCPPGGTGSQGHIAEIM

DLQGTKIYDWIKAYGSTFMVRGPFGVHHRIFSVDPRVLNHVLKHTNIYTKSDLLRDLVRR

YMKEGLIVAEGERHKVQRKVSQKLFSMGGLKSMGQVVQDKSNQLRDILLNLCANLTASNP

YSPVNPTLSGSREVDVYSTVSRCTFDLIGSIGVGHQFDSLGNWEGSGGKLFQKYERMQLL

CPGAMGFRMLLSLTWPLIDKIWPSENTKRVNDAMGSLQKFAKEKMIERQLELATVDSKKG

DIPDRKDLLILMLRHNLAQKINAADKLRDHEIFGQLSTFLFAGSETTAGTISFGLYDLAY

HPDIQSRLRAEILECGDNLSFDQIDELPYLDAVVKEIMRNPSLPGTVRQAQKDDIIPLAK

PVTLTNGKVVADIHIRKGQLVSSFLFLMQSLFRPLKG

>CYP5215A1(AFR93529.2)Cryptococcus neoformans var. grubii H99

MMLKQLSFQPPTMIGSILLALVILVVWLLHVFVYKSFTSALKNVPCPPGGNGSQGHIGEI

MNLQGIEVHQWIRTYGSTFMVRGPFGVHHRIFTIDPRALSHVLQHTNIYTKSDLLRDLVR

RYMKEGLIVAEGERHKVQRKVSQKLFSMGGLKSMGQIVQDKSNQLRDILLNLCANPTASN

PYSPVNRTLPPGSREVDVYSAASRCTFDLIGSIGVDHQFDSIADWEGSGGKLFHKYEQMQ

LLCPGAMGIRMLLSLTWPLVDRIWPSENTKRVNDAMGSLEKFAKEKMIERQQELLTMDSK

KGDVPDRRDLLTLMLRHNMSRKISPADKLRDHEISGQLSTFMFAGSETTAGTISFGLYDL

ARHPDVQSRLRAEIFECGDNLPFDQIDELPYLDAVVKEIMRINPSLPGTVRQAQKDDIIP

LAEPVTLTNGKVGTDIHIQKGQLVHVPIEHLHTSEHIWGPTAKEFDPSRFISTSQPAAFS

DQLTLASNPVATFSARRDAVPSYVPEGPGIWPNFMTFIDGPRRCIGYKLAVMEIKTVIFT

LLREFEIEPVEGQHIFRWNMMSNRPFVANTLWSKGSRLPLHFKLYKGEQ

>CYP5215A1(EAL22841.1)Cryptococcus neoformans var. neoformans B-3501A

MLKQLSFQSPRTIEIIPLALVVLVVWLFRVFVYKPFTSPLKNVPCPPGGTSSQGHIAEIM

NLQGTEVNQWIKTYGSTFMVRGPFGVHHRIFTVDPRALSHVLQHTNIYTKSDLLRGLVRR

YMKEGLIVAEGERHKAQRKVSQKLFSMGGLKSMGQVVQDKSNQLRDILLNLCTNPTASNP

YSPVNRTLPPGSREVDVYSTASRCTFDLIGSIGVDHQFDSIGDWEGSGGKLFHKYERMQL

LCPGTTGFRMLLSLAWPLIDKIWVNNIFPLPSENTKRVNDAMGSLEKFAKEKMIERQQEL

LTIDSKKGDVPDRGDLLTLMLRHNMTRKISPADKLRDHEISGQLSTFMFAGSETTAGTIS

FGLCDLARHPDIQSRLRAEILECGDNLPFNQIDELPYLDAVVKEIMRINPSLPGTVRQAQ

KDDIIPLAEPVILTNGKVVTDIHIRKGQLVGSFLFFIQSLFHS

>CYP5215A1(AAW41954.2)Cryptococcus neoformans var. neoformans JEC21

MLKQLSFQSPRTIEIIPLALVVLVVWLFRVFVYKPFTSPLKNVPCPPGGTSSQGHIAEIM

NLQGTEVNQWIKTYGSTFMVRGPFGVHHRIFTVDPRALSHILQHTNIYTKSDLLRGLVRR

YMKEGLIVAEGERHKAQRKVSQKLFSMGGLKSMGQVVQDKSNQLRDILLNLCTNPTASNP

YSPVNRTLPPGSREVDVYSTASRCTFDLIGSIGVDHQFDSIGDWEGSGGKLFHKYERMQL

LCPGTTGFRMLLSLAWPLIDKIWPSENTKRVNDAMGSLEKFAKEKMIERQQELLTIDSKK

GDVPDRRDLLTLMLRHNMTRKISPADKLRDHEISGQLSTFMFAGYETTAGTISFGLYDLA

RHPDIQSRLRAEILECGDNLPFNQIDELPYLDAVVKEIMRINPSLPGTVRQAQKDDIIPL

AEPVILTNGKVVTDIHIRKGQLVHVPIEHLHTSEHIWGPTAKEFNPSRFLSTTQPSAFSG

RPTLASNPAATSSARRDAVPNYVPEGPGIWPNFMTFIDGPRRCIGYKLAVMEIKTVIFTL

LREFEIELMEGQHIFRWNMMSNRPFVANTLRSKGSRLPLHFKLYKGEQQHEKTGGS

>CYP5878A1(41150)Cryptococcus curvatus

MEHAQSLLSALDTWPVRVVLGLTLFYVCLRLAFLPPYSPKQTREVANLPGPPPVPYFGWL

IGNMADLAEYRDTVMHPDWVAMGWTGRCQHIMGQETIWTYDPVAMGSILQSADVWERAKN

TERLLRRITGSGMLTAKGAAHRRQRRIVNPAFSTTAIKAMVPAMFEKAELCATLLGRCVD

DDSLESFASRYPPKPEDRVPGARKVDMLALCSKLTQDVIGAAGFNTDLESLRPADNPLDN

SIQYMLNTIFDDTIISMGQNLYWLLDKIPTRTRRAMKTCRTEMRVSERLMTERAAKQEGS

VVDADEDKVPDMLDLLLKANSAEREDQRLSDEEVRSQLSTLLFAGSTTTAGTICSLLRFL

ALHPDIQARLREEIEATEERPSFETLNALPLLDATVRETLRLEPAATCTVRTNTKDTVIP

LSVPVRGRDGTMIEKALPVAKGSYVLLSISSLQRHPDVWGPDAEEFNPDRYKDPSIPKMN

IPGMWGGLASFISGPHHCIGWRLALAEIKIVTFTLLRHFAFEELPSKPDIFIVMNVASRP

EVKGESGGQMPLLVRRIEA

>CYP5878B1(365317)Cryptococcus curvatus

MANTTLRVPLLPFSGGWASIPLASWWAPLALTLALWLISRLLKPPHSPRNTPEVRALPGP

ARPRFGWLLGNMAQLADHRETLIHPDWFQWGWTGVYESLFAQKRIYTFDPAAVGYIFAHP

DEFERPRDLRTILGHIMGADSLVVNEGEKHRRMRRALNPAFSAGAIKEMMPAMLEKAGEM

VALFERLIDEEALESYAARAPAPEDRVPGARKIDVFKFAGNLTTDIIGIAGFEHDFNSLS

PEDDSPALLANRFNRLVDAANGMVLLTEAQNIMPVLDKIPTQSRRVIGEVRGQMERLSDT

LFARRKEVGYDPGAKDLLSLLVKSASATKSDAAMSHDEVRAQMASLLFAGSTTTAATISF

ILFELAKLSEWQEKLRAEVLAGHARPSFEALNAFPLLDAVIRETLRLHPPASCTCRATTK

DMVLPLAKPIPLRDGRTIDALPVSKGSYFFISMVCLNRLKEIWGDDAEEFNPARHADPAL

PRMQVPGVWGNVSSFISGPHACIGYRLAIIEIKVVLHALLRAFEFELLPSRPHIQHFSSL

ATQPQVKGEPGGQMPLLVRRHRV*

>CYP5139B1(KGB75861.1)Cryptococcus gattii VGII R265

MELFKTLHLETSQFFSNCIQPSPVACIALYTFTGVTILLFTIYLWLWPFQYATLHFRNLP

GPPSDSWFWGVVPTLIKSPPSVPHSMWTDEYGPTIRYRVALGAQRFLTIDPTALNYILSH

IDLFPKPSRVRKALSDLLGNGLLTAEGYTHKKQRKALNPSFSPAAIRGMVPVFYDKAYEL

KAKLLGIISDDETEQASPTPCIKEDEVEGGKKIDVMKYLGKTTLDVIGIVGFSYDFKALS

EPHNELSEAYSKMFQASMDANFWDFLRGAIPLVNKLPNKRATEIAARKAVTLRIGKKIVE

DKKREVMSAHSEGLEKREDIGNDLLSILIKANMASDVKPEQKLSDEEVLDQITTFMLAGN

ETSSTALTWILYSLTQHPECQERLREEVLAVADDRPSLETLNSLPYMDAVIRETLRLNAP

APGTLREAKQDTVIPLSMPVTGRDGKQINSVRINKGTVVFIPILTVNTSPAIWGPDARDF

NPDRHLKTASNSFGGANMHVPGVWGNMLSFLGGARNCIGYKLALAEISTILFVLMRSFEF

QELKSKPEVEKKASVVMRPRIKGEESAGLQMPLMVKPL

>CYP5139B1(CGB_F5030W)Cryptococcus gattii WM276

MELLRHLGTWQFFSNCIRPSSLACIALYSFGGITILLFTVYLWLWPFQYATLHFRNLPGP

PSDHWFWGVIPTLIKSPPSVPHNIWTDEYGPTIRYRVALGAQRFLTIDPTALNYILTHVD

IFPKPSRVRKALSDLLGNGLLTAEGYTHKKQRKALNPSFSPAAIRGMVPIFYDKAYELKA

KLLGIISDDETEQASSTPCIKEDEVEGGKKIDVMKYLGKTTLDVIGIVGFNYDFKALSEP

HNELSEAYSKMFQASMDPDIWDFLRAVIPLVKKLPNKRAAEIAARKAVTLRIGKKIVEDK

KREVMSAHSEGLEKREDIGNDLLSILIKANMASDVKPEQKLSDEEVLDQITTFMLAGNET

SSTALTWILYSLTQHPECQERLREEVLAVADDRPSLETLNSLPYMDAVIRETLRLNAPAP

GTLREAKQDTVIPLSMPVIGRDGKQINNVRINKGTVVFIPILTVNTSPAIWGPDARDFNP

DRHLKTSSNSFGGANMHVPGVWGNMLSFLGGARNCIGYKLALAEISAILFVLMRSFEFQE

LKSKPEVEKKTSVVMRPRIKGEESAGLQMPLMVKPL

>CYP5139B(2740584893)Cryptococcus amylolentus CBS 6273

MPLPSLFGALFPAFHLHSWVHIALSLLALLAATLFATYLYAYPIRLARLQFYNLPGPEPE

SFLWGSLPTLLRSEPNVPQGEWTAKYGHTIRYRAQLGAQRFITLDATGLNYILSHPDVFP

KPDVARRALADMLGNGLLTSEGEDHRRQRRALNPSFSVGAIKNMVPTFYDKAYELQAKLL

ALVENDDPSEKASPTPAKEEDFVPGGKKIDMMKYLGKTTLDVIGVTGFGYDFKTLSEPNN

ALSDAYAQMFSAGMTVNFKDFVLNRIPLIRQFPTEKSRTIASSRKITRDIGAKLVKDKKE

AVMASFGDDLEKGQDIGKDLLSILIQANMAADLKPEQRLTDEEVLNQITTFMLAGNETSS

TGLSWTLYNLSLNMDVQAKLREEVMSIPDERPDVETLNSLTYMDAVLREALRLCPPAPGT

IRQAKEDVVIPLGVPLKGRDGTMMESVGINKGTIVFIPITNVNCSEAIWGPNASKFDPSR

FLSSSTEVKPTHVPGVWGNLLTFLGGTRNCIGYKFALAEMKVILFVLMKNFEFQELKSKP

EVEKKSAVVMRCRIKGEEDAGLQMPLMVVPLEAV

>CYP5139B1(KIR99883.1)Cryptococcus gattii VGII 2001/935-1

MELFKTLHLETSQFFSNCIQPSPVACIALYTFTGVTILLFTIYLWLWPFQYATLHFRNLP

GPPSDSWFWGVVPTLIKSPPSVPHSMWTDEYGPTIRYRVALGAQRFLTIDPTALNYILSH

IDLFPKPSRVRKALSDLLGNGLLTAEGYTHKKQRKALNPSFSPAAIRGMVPVFYDKAYEL

KAKLLGIISDDETEQASPTPCIKEDEVEGGKKIDVMKYLGKTTLDVIGIVGFSYDFKALS

EPHNELSEAYSKMFQASMDANFWDFLRGAIPLVNKLPNKRATEIAARKAVTLRIGKKIVE

DKKREVMSAHSEGLEKREDIGNDLLSILIKANMASDVKPEQKLSDEEVLDQITTFMLAGN

ETSSTALTWILYSLTQHPECQERLREEVLAVADDRPSLETLNSLPYMDAVIRETLRLNAP

APGTLREAKQDTVIPLSMPVTGRDGKQINSVRINKGTVVFIPILTVNTSPAIWGPDARDF

NPDRHLKTASNSFGGANMHVPGVWGNMLSFLGGARNCIGYKLALAEISTILFVLMRSFEF

QELKSKPEVEKKASVVMRPRIKGEESAGLQMPLMVKPL

>CYP5139B1(KIY58583.1)Cryptococcus gattii VGII 99/473

MELFKTLHLETSQFFSNCIQPSPVACIALYTFTGVTILLFTIYLWLWPFQYATLHFRNLP

GPPSDSWFWGVVPTLIKSPPSVPHSMWTDEYGPTIRYRVALGAQRFLTIDPTALNYILSH

IDLFPKPSRVRKALSDLLGNGLLTAEGYTHKKQRKALNPSFSPAAIRGMVPVFYDKAYEL

KAKLLGIISDDETEQASPTPCIKEDEVEGGKKIDVMKYLGKTTLDVIGIVGFSYDFKALS

EPHNELSEAYSKMFQASMDANFWDFLRGAIPLVNKLPNKRATEIAARKAVTLRIGKKIVE

DKKREVMSAHSEGLEKREDIGNDLLSILIKANMASDVKPEQKLSDEEVLDQITTFMLAGN

ETSSTALTWILYSLTQHPECQERLREEVLAVADDRPSLETLNSLPYMDAVIRETLRLNAP

APGTLREAKQDTVIPLSMPVTGRDGKQINSVRINKGTVVFIPILTVNTSPAIWGPDARDF

NPDRHLKTASNSFGGANMHVPGVWGNMLSFLGGARNCIGYKLALAEISTILFVLMRSFEF

QELKSKPEVEKKASVVMRPRIKGEESAGLQMPLMVKPL

>CYP5139B1(KIR93615.1)Cryptococcus gattii VGII CBS 10090

MELFKTLHLETSQFFSNCIQPSPVACIALYTFTGVTILLFTIYLWLWPFQYATLHFRNLP

GPPSDSWFWGVVPTLIKSPPSVPHSMWTDEYGPTIRYRVALGAQRFLTIDPTALNYILSH

IDLFPKPSRVRKALSDLLGNGLLTAEGYTHKKQRKALNPSFSPAAIRGMVPVFYDKAYEL

KAKLLGIISDDETEQASPTPCIKEDEVEGGKKIDVMKYLGKTTLDVIGIVGFSYDFKALS

EPHNELSEAYSKMFQASMDANFWDFLRGAIPLVNKLPNKRATEIAARKAVTLRIGKKIVE

DKKREVMSAHSEGLEKREDIGNDLLSILIKANMASDVKPEQKLSDEEVLDQITTFMLAGN

ETSSTALTWILYSLTQHPECQERLREEVLAVADDRPSLETLNSLPYMDAVIRETLRLNAP

APGTLREAKQDTVIPLSMPVTGRDGKQINSVRINKGTVVFIPILTVNTSPAIWGPDARDF

NPDRHLKTASNSFGGANMHVPGVWGNMLSFLGGARNCIGYKLALAEISTILFVLMRSFEF

QELKSKPEVEKKASVVMRPRIKGEESAGLQMPLMVKPL

>CYP5139B1(KIR86328.1)Cryptococcus gattii VGIV IND107

MELLRTLHLETSQFFSSCIRPSSVACIALYTFGGIAIPLFTIYLWLWPFQYATLHFRNLP

GPPSDHWFWGVIPTLIKSPPSVPHSIWTDEYGPTIRYRVALGAQRFLTIDPTALNYILSH

VDIFPKPSRVRKALSDLLGNGLLTAEGYTHKKQRKALNPSFSPAAIRGMVPVFYDKAYEL

KAKLLSIINDDETEQASPTPCIKEDEVEGGKKIDVMKYLGKTTLDVIGIVGFNYDFKALS

EPHNELSEAYSKMFQASMDPDFWDFLRAVIPLVKKLPNKRAAEIAARKAVTLRIGKKIVE

DKKREVMSAHSEGLEKREDIGNDLLSILIKANMASDVKPEQKLSDEEVLDQITTFMLAGN

ETSSTALTWILYSLTQHPECQERLREEVLAVADDRPSLETLNSLPYMDAVIRETLRLNAP

APGTLREAKQDTVIPLSMPVKGRDGKQISSVRINKGTVVFIPILTVNTSPAIWGPDARDF

NPDRHLKTSSNSFGGANMHVPGVWGNMLSFFGGARSCIGYKLALAEISTILFVLMRSFEF

QELKSKPEVEKKASVVMRPRIKGEESAGLQMPLMVKPL

>CYP5139B1(KJE04206.1)Cryptococcus gattii NT-10

MELLRHLGTSQFFSNCIRPSSLACIALYSFGGITILLFTVYLWLWPFQYATLHFRNLPGP

PSDHWFWGVIPTLIKSPPSVPHNIWTDEYGPTIRYRVALGAQRFLTIDPTALNYILTHVD

IFPKPSRVRKALSDLLGNGLLTAEGYTHKKQRKALNPSFSPAAIRGMVPIFYDKAYELKA

KLLGIISDDETEQASSTPCIKEDEVEGGKKIDVMKYLGKTTLDVIGIVGFNYDFKALSEP

HNELSEAYSKMFQASMDPDIWDFLRAVIPLVKKLPNKRAAEIAARKAVTLRIGKKIVEDK

KREVMSAHSEGLEKREDIGNDLLSILIKANMASDVKPEQKLSDEEVLDQITTFMLAGNET

SSTALTWILYSLTQHPECQERLREEVLAVADDRPSLETLNSLPYMDAVIRETLRLNAPAP

GTLREAKQDTVIPLSMPVIGRDGKQINNVRINKGTVVFIPILTVNTSPAIWGPDARDFNP

DRHLKTSSNSFGGANMHVPGVWGNMLSFLGGARNCIGYKLALAEISAILFVLMRSFEFQE

LKSKPEVEKKTSVVMRPRIKGEESAGLQMPLMVKPL

>CYP5139B1(KIR82527.1)Cryptococcus gattii EJB2

MELLRHLGTSQFFSNCIRPSSLACIALYSFGGITILLFTVYLWLWPFQYATLHFRNLPGP

PSDHWFWGVIPTLIKSPPSVPHNIWTDEYGPTIRYRVALGAQRFLTIDPTALNYILTHVD

IFPKPSRVRKALSDLLGNGLLTAEGYTHKKQRKALNPSFSPAAIRGMVPIFYDKAYELKA

KLLGIISDDETEQASSTPCIKEDEVEGGKKIDVMKYLGKTTLDVIGIVGFNYDFKALSEP

HNELSEAYSKMFQASMDPDIWDFLRAVIPLVKKLPNKRAAEIAARKAVTLRIGKKIVEDK

KREVMSAHSEGLEKREDIGNDLLSILIKANMASDVKPEQKLSDEEVLDQITTFMLAGNET

SSTALTWILYSLTQHPECQERLREEVLAVADDRPSLETLNSLPYMDAVIRETLRLNAPAP

GTLREAKQDTVIPLSMPVIGRDGKQINNVRINKGTVVFIPILTVNTSPAIWGPDARDFNP

DRHLKTSSNSFGGANMHVPGVWGNMLSFLGGARNCIGYKLALAEISAILFVLMRSFEFQE

LKSKPEVEKKTSVVMRPRIKGEESAGLQMPLMVKPL

>CYP5139B1(KIY33850.1)Cryptococcus gattii E566

MELLRHLGTSQFFSNCIRPSSLACIALYSFGGITILLFTVYLWLWPFQYATLHFRNLPGP

PSDHWFWGVIPTLIKSPPSVPHNIWTDEYGPTIRYRVALGAQRFLTIDPTALNYILTHVI

FPKPSRVRKALSDLLGNGLLTAEGYTHKKQRKALNPSFSPAAIRGMVPIFYDKAYELKAK

LLGIISDDETEQASSTPCIKEDEVEGGKKIDVMKYLGKTTLDVIGIVGFNYDFKALSEPH

NELSEAYSKMFQASMDPDWDFLRAVIPLVKKLPNKRAAEIAARKAVTLRIGKKIVEDKKR

EVMSAHSEGLEKREDIGNDLLSILIKANMASDVKPEQKLSDEEVLDQITTFMLAGNETSS

TALTWILYSLTQHPECQERLREEVLAVADDRPSLETLNSLPYMDAVIRETLRLNAPAPGT

LREAKQDTVIPLSMPVIGRDGKQINNVRINKGTVVFIPILTVNTSPAIWGPDARDFNPDR

HLKTSSNSFGGANMHVPGVWGNMLSFLGGARNCIGYKLALAEISAILFVLMRSFEFQELK

SKPEVEKKTSVVMRPRIKGEESAGLQMPLMVKPL

>CYP5139B1(AFR96161.2)Cryptococcus neoformans var. grubii H99

MELLKALHHETLQLFPDCIRSSSVACIVLYSLSGIAILLSTVYLWLWPFQYAKLHFRNLP

GPPSDSWFWGVIPTLIKSPPSVPHSMWTDEHGPTIRYRVALGAQRFLTIDPTALNYILSH

ADLFPKPSRVRKALSDLLGNGLLTAEGYTHKKQRKALNPSFSPAAIRGMVPVFYDKAYEL

KAKLLSIIEDETEQASPTPCKEEDEVEGGKKIDVMKYLGKTTLDVIGIVGFSYDFKALSE

PHNELSQAYSKMFQAGMDANFWDFLRGAIPLVNKLPNKRATEIAARKAVTLRIGKKIVED

KKREVMSAHSEGLEKREDIGDDLLSILIKANMASDVKPEQKLSDKEVLDQITTFMLAGNE

TSSTALTWILYSLTQHPECQKRLREEVLAVPDDRPSLETLNNLSYMDAVIREALRLHAPA

PGTMREAKEDTIIPLSMPVTGRDGKQIDSVKINKGIMVFIPIMTVNTSPAIWGPDARVFN

PDRHLKASSNSFGGANMHVPGVWGNMLSFLGGARNCIGYKLALAEISTILFVLIRSFEFQ

ELNSKPVVEKKASVVMRPRIKGEESAGLQMPLMVKPLLM

>CYP5139B1(EAL20013.1)Cryptococcus neoformans var. neoformans B-3501A

MTMELLKVLHHGASQLFPNCIRSSPVACIVLYSFGGIAILLFTVYLWLWPFQYAKLYFRN

LPGPPSDSWFWGVVPTLIKSPPSVPHSMWTDEYGPTVRYRVALGAQRFLTIDPTALNYIL

SHADLFPKPSRVRKALSDLLGNELLTAEGHTHKKQRKALNPSFSPAAVRGMIPVFYDKAY

ELKAKLLGIIEGDETEQASPTPCKEEDEVEGGKKIDVMKYLGKTTLDVIGIVGFSYDFKA

LSEPRNELSEAYSKMFQAGMDANFWDFLRGAIPLVNKLPNKRATEIAARKAVTLRISKKI

VEDKKREVMSAHSEGLEKREDIGDDLLSILIKANMASDVKPEQKLSDEEVLDQITTFMLA

GNETSSTALTWILYSLTQHPECQKRLREEVLAVPDDRPSLETLNNLPYMDAVIREALRLH

APAPGTMREAKEDTVIPLSMPVIGRDGKQIDSVKINKGTMVFIPIITVNTSPAIWGPDAR

VFNPDRHFKTSSDSFGGANMHVPGVWGNMLSFLGGARNCIGYKLALAEISTILFVLIRSF

EFQELKSKPEVEKKASVVMRPRIKGEESAGLQMPLMVKPLLM

>CYP5139B1(AAW43969.1)Cryptococcus neoformans var. neoformans JEC21

MTMELLKVLHHEASQLFPNCIRSSPVACIVLYSFGGIAILLFSVYLWLWPFQYAKLYFRN

LPGPPSDSWFWGVVPTLIKSPPSVPHSMWTDEYGPTVRYRVALGAQRFLTIDPTALNYIL

SHADLFPKPSRVRKALSDLLGNGLLTAEGHTHKKQRKALNPSFSPAAVRGMIPVFYDKAY

ELKAKLLGIIEGDETEQASPTPCKEEDEVEGGKKIDVMKYLGKTTLDVIGIVGFSYDFKA

LSEPRNELSEAYSKMFQAGMDANFWDFLRGAIPLVNKLPNKRATEIAARKAVTLRISKKI

VEDKKREVMSAHSEGLEKREDIGDDLLSILIKANMASDVKPEQKLSDEEVLDQITTFMLA

GNETSSTALTWILYSLTQHPECQTRLREEVLAVPDDRPSLETLNNLPYMDAVIREALRLH

APAPGTMREAKEDTVIPLSMPVIGRDGKQIDSVKINKGTMVFIPIITVNTSPAIWGPDAR

VFNPDRHLKTSSDSFGGANMHVPGVWGNMLSFLGGARNCIGYKLALAEISTILFVLIRSF

EFQELKSKPEVEKKASVVMRPRIKGEESAGLQMPLMVKPLLM

>CYP5139AK1(2740582810)Cryptococcus amylolentus CBS 6273

MGALDVLPAHLPTVLRDAPPLYKASATLLALVLATYLWLFPIANARTPFRNLPDPGPGHW

LLGHLMDVFLSPTPNATNIAFHKAHGPTVKYRTQLGRFEVSTIDPTAIAYVLNHPDIFVK

PPALRDWLYSRIGNGLVTAEGEHHKKQRKAMIPSFSPAAIRELAPVMLDAAYELKDKLEG

LIAHKDEYTLSPSPAKPIDAVPGGAKVNVLRYLNMSTFDVIGLTGFGHSFGALSERKNGL

TASMQRFLRNMFAAGLADYLFGLYQFQLPTEKNRVVNKEKQEILEFARWIAAERKREILE

QSHGEGINKKQDIGKDLLSLLSESFRRLLCITELTCWTVKANMASDMKPNERLTDEDVAD

QILTFLVAGSETSATSVSLYLDIISQHSDVQDRLRKELLSVNEERPSIETLDNLPYLEAV

MRECLRFFPAASFVFRHATEEATVPLGTPVIGKNGKLMTEFKMDTVNMLFIPVRAVNTST

TLWGPDAESFNPSRFLDDDRTPGTGTGGKSSTKVPGVYGNLMTFIGGPRNCIGYKFAIQQ

MKILLFVLLRNFEFRPLKSGSEMVYRPSAVVQAYVKGEEHLGVQIPLMVIPLAGAGHDKD

E

>CYP5139AK2(2740582808)Cryptococcus amylolentus CBS 6273

MAPVFYDVAYQLCDKFRQLVADKNNAGLSPSPPRPIDAVSGGAKTEVLKYINMATFDVTG

LAGFGHAFEALSEKPNRLTSCMHRYIAAFFEARLSDYLAGTYHIQIRSESRLTPPKRIAG

ERKNDILQQSFGEGINKKQDIGNDLFSILIKANMAFDLEPHERLSDEVLADQLLTFARSE

TVATSLSIFLRLLSEHPDVQNRLRTELVSVDDDRPPVEVLDNLPYLDAFVREALRFFPAA

PWVHPSATGNATVPLGTPIKGKDGKLITSFNIDERSYIFLPLRAMNISTTFWGPDAETFD

PSRFLDHRTVNMAKLPGVYGNMISFLAGPRNCIGYRFALLQMKVMLFALLRDLEFRELKS

KPETVYRPSPAVRAYVKGEEHLGVQAPLMVIPLDNSGHVSKDRC

>CYP5139AK3(2740582809)Cryptococcus amylolentus CBS 6273

MDTLPTVLRDLPPLYKAPATLLALAFATYLWLFPIANARIPFRNLPDPGPGHWLLGHALA

NFHPPSPNAAHIDLHKTHGHTIKYRTQLGNFEVSTIDPTAISYIVNNPNIFVKPPAARKW

LEWRIGHGVSTAMGDDNKHQRRLMGPSFSAAAMRDMVPVFHDVAYELSDKFRSPVVDREG

APSPPSPSPPKPIDAVPGGGKIDVLKYIGMATFDVMGLTGFGHAFRALSEQPNTLTDCMH

SYLAAFFKTGISDFLAAMYSIQLVICQRESLLSAQTVAPLTLASKRIAREKREQIFEQTH

GEGIHKKQDVGKDLLSILIKANMASDLKPNERLSDEVVADQIMTFLVAGSETVATSLSFF

LDLLGKHPNIQDRLREELMSVNEERPSAEVLDSLTYLDAFVREALRFCPAGPWMYRLATE

PVAVPLGTPVKGKDGKMMNSFTIDSSTFVFTPIRALNTSTSFWGPDAETFDPSRFLDGRS

ETATKLPGVYGNTMTFMAGPRNCIGELGSRPERVSKISLVVRAFVKGEEHLGVQVPLMVV

PLGGSSRDKEVV

>CYP5139AS1(420274)Cryptococcus werringae

MAQLIPELLQRILTLIPLSHLLYLASLYALFLLVNRYFLRPAFAPYRNLPGPTRRESWGP

IIGHLRPIIKGLPGEAAGKWFERFGRVFRESPCDRSGQRMVWLNTSNQSFLIEYEGIFGT

PRLMVGDPTAISYILQHTEIFQKDDLTRTFLRRILGEGLLNVEGSDHRRQRKVMNPAFGP

GLIRDHHLPIFYDKAYQLKEVLLGLISSPSSSGSGSEASSQGIRMDISKWLGKTTLDVIG

LAGFGYDFNSLHDPTNELALAMERMFTATQNPSLLHFLTEFIPFGKYIPSKRKRYQKESA

DTTHRIGTALLEEKTAAVRAGEASLEKGKGKDLLSMVVRANMEPSLPLNARMSDAEVIAT

IQTFVLAGAETSSTALTWGSWVLAKMPQVQDALRKECEAVLEDEPSMDELNALPYLDGFV

HEVLRLHSPVPQTRRDSISETVIPIGKPVRGKDGQMMDSIRVPKGMACMLAIINVNKSTE

WGPDALEFKPERWLNKEKSPVLEPVGGVWGNMFSFIAGPRACIGFRFSLIEIKCILFVLI

RNFAFAEAVPTEDVIAKTAIVTRPTIRGEEKTGPQMPLYLRALA*

>CYP5139AS1(817600)Cryptococcus terricola

MSFLTTISSDLNNLLSFLPPLTLLNSIAAFIVLIAGGAFYQIFWKQWMLPYKNLPGPWHW

SPILGSMGPVIKAEPGQQHKLWMKKYGHTMYYTGFLFEPRLFTTDPQALQYILSHADDFP

KSEGIRRSLAGILGKGVLFAEGEDHRRQRKIMNPSFAPNQIRDHYTPIFLNKAYELRDCF

TRLVPESSTESIRIDTYQWLGKCTLDVIGLAGFDYDFGSLSNPDNELAKAYSKMFAASTT

INALAILQSIIPGLNKIPTERVKTQRASLETTRRIGKELVEKKKQAALESAKAGDNGAGY

VGKDLLSVVVRANMAHDLKESQRMTDEEVLGQITTFMLAGQETSSTALNWGLYMLSLHPD

VQDKLRAELLSVQEDMPSMDTLNSLEYLDAFVHEVLRLLAPVPSTRRDSVVDSVIPLSKP

VKGRNGKMIHSVFLPKGTPVIIPILNTNTDPELWGEDSWEFKPSRWLKGEDGVVHAVSES

VPGVWGNMMTFIGGPRACIGYRFAIVEIKAILFVLVRSFVFEAIQPAIDIEKKTSLVTRP

VIRSEKDKGAQMPLMVRVVS*

>CYP5698B1(320875) Naganishia vishniacii v1.0

MPTPVALIISSLGVVVVTCIISRLRQNSSKKIAVPWAQNSTPFVGHVLAYGADPVKFLLS

QKELLGDVFRINLVIIKITFCVGAQWNRWLLRETSEADVSFYRLIPKISCGLIPERLLHT

GWIHHSLKALRLGLNRPERLKQATQMSAQISSQLYAEWAESATILLFAQASKLLLLANLV

IFFGRDVVTDYESELIAAVKAYQKALVCPWARLLPLWASPNGRMMARAQAYFERIIQGEA

ETRLKDLDGCREAEDYLSYLLTLNHDEGFQESYSEYAAHFIILAHVNTAGTYAWTLLHLL

RNPDLLSEFENEIAHNSPADDGTYSFKDMLFSEACLRETGRLYGNMVVLRYVPKDLCAPD

GTIIPQGFAAASPLAAHYDPSLFPNPGKWNPRRFMPASDGTPSTFNALFRNTEFYQFGAG

KHTCPGERLTNCLLRGNLWPALLDNYRLELVDGLVDGEGVDEVGVNPNHGESLGTPYGVR

EVSIKVTKRPVALSAAPLSASLSAKIEA*

>CYP5698A1(326953) Naganishia vishniacii v1.0

MSPSVLLAIILSIIVILWQSLQRTRPTSTVPWTRTGLPLVGSAMAFTAEPIQFLLDQQQR

LGNVFRVDLLVMRVTFVIGPQWNRWLLREAKEEQVSFWRAILAMHRGLIDTSMHFPGWLD

RAVKALGTGMNRPARLEAYERTFSRVAQRIFSDEWSTATSPIPLFQGSSALILWCTMTAF

YGSAFVHRHGEEMIPLVREFERLFVKPGVRVLPLWASADGRRLKHVYGRMKELIEDEVRR

RLVDDGLKENSEEEGDYLGFLLRMDDRAEDFFECYGEHMMTIISASHVNTAATFSWTLLH

LLRNPKHLATIHAEITAHPPNSHGVYPYREMPFSDACLRETGRLYTNLMMLRYLPNEVER

TPDGTVLSQGWVAASPLTTQQDPSLHPEPKSWNPGRWLAGGTYGSKFRSQEFVQFGYGAH

ACLGERFTHSLLRGVLWPLLLDGYELEIMGGLREGEGMDGVGVSANWQESMGPMGVREIS

LTVRKRKERMSGNPTGEIQAERVIIMVNSD*

>CYP5698A2(778392)Cryptococcus terricola

MTLIVAYRLLSPASGTNVPWAKTSIPFLGGAIGYGKQPVNFLVEQRQKVGDVFRINLLVM

KMTFVIGSKWNQWLLKNTKEEDVSFWNAMLALNCGLLDTSFHYPGWVDRGTKALINGMNR

PARLEKYSEEIAHVAASLFDSWSTEPRVPLFISSSDLLLQASMTTLYGPAFVKRHATELI

PIVRAFERAFVHPLTRVLPLWMSPSGRVLLRAYRRMKEVIEAEVRMRLKDLERWRGEGDY

MSYLLTMDDKAEDFFACYGEHLMIMISASHVNTAGTFSWTLLHLLRNPDLLAQFEDEIRH

NPPTNGIYPAKNMPFSEACMRESGRLYTNLLLVRYATHDLMTPDGMVIPKGWIAASPLAT

QQDPELYPHPDEWDPHRFLVSAGPGDYSSKFRNNEFVQFGYGKHACLGEKLTHTLLRSNL

WPTLFDGYQVEIVDGVIEGEGIGGVGVKPNFRENLGTPFGDREVWVRVRKRAVKLTEVE*

>CYP5231B1(315405) Naganishia vishniacii v1.0

MVNLTPPSTLTGLFPAHFSTLHLILLAFAIYYRTYVYPYRNLPPGPPGHWLFGNMYQIPK

SRPWVQMAKWSEQYGPMYTIWMGPTKPVLIVGNAAIANELLDKRGAIWSSRPRMIMTSEL

VSRGLRMTFMPFTDQWKQQRKLLHTMTQPRAATTYEPIQDEESRQLVQDMLTRPSDYWSH

CQRYAGSTIMQIAFNKRALTITDPAIIDMRRINEQMTKTAVPGRYMVDSLPILNYIPERF

ASWKQEASTLYEDTLRLFRSHVDDVYREVKSGSDVNCFAKTILENKDKYGLSNDEATFLA

GAMYGAGSDTTADMISTAIMTFTKYPSVVAQAQAELDRVVGRDRLPTFEDEKDLVFCSAV

VREIMRWRTVIAGGLAHASIKDDFYEGYHIPAGTTLIPNHWSIHLDPKLYPDPETFKPER

FIQDGKLVGTAQSERGHHSYGFGRRICPGMYIADRSMFITFTRLLWCFTFTEDPRHPIDA

DAFSEGFSSHPLPFKSAIVPRGPWVEDVSKGEH*

>CYP5699A1(344597) Naganishia vishniacii v1.0

MQYSSFLAAVVLRNVLLPLVLASLAVRGCSRSFPLVIRFAIYIAALPLNHLLLSRLSVAR

QTWAARRLHPEAIPIPRVRGRWPLNLDVMLDWMESSKGEYLAEGMVSKLEQTYGKTINTR

VLGEDSIITTSAQHVSHILNASFPAFEKGPKWKERVHEMIGDGVFGSDGELWKLHRATTR

PFFSRSRLPLDEILEPHTANYITALKRFFHSTTSIAADTGPPAFDVQELTRRLTLDVAMS

WFCGHTTGLLDDALGTDNRPGKACSGKPEGVKVFEAFAEAQAVASMRIKIGTLWSLFEWR

ENQMSTPMMTIRGFIRPIVANAIRATEARPASVRATSVSDAIEKRREETLLDYLVQVLDD

PKIIEDEIINILVAASDTTAAALTASVYCLAMYPECLTKIAQEAHRLSPEHIIADDLKSC

EYLNAFIHEVLRLYPPVPLNIRRSNRAVLLPPHDINSPPLVFPAHTSVILSTLAIQRDES

IWGPDAQSFDPSRWLDEDFRSQEDYLYGIPRYGRAAFPAFHLGPRSCLGQTFALQQLAYT

VLRLVEALRPSAPEAKIYRFAFATEAQPAGTTVPAKWKSLIGRGREERAFWSSSLTLYFK

GGLWMNIIDHGE*

>CYP5700A1(345215) Naganishia vishniacii v1.0

MSPVLQIPSSFTLLLAIVGLIPLAVIYFWINRPCAQAQFAQLPPGPKPHWLLGNELPSKY

PWRYFEELTKQYGDQGGEGCITIWQGQTPLVIVGRVPAAQELLEKQAGATADRPRSIAGN

IMSGRMRILLIGYGDHWRKLRKALHSHLSPVSARSYEKVQERAAKRVLGDILSCPKAHQD

HVKTYAATIVLNIAYGRTERVSYTDPDIQLVNQCGDRLGQTLRPGSFMVESLPWLRYVPG

YTKTIDQWHSDELTLFRGQLDMARERLKSSSVACFTRFISEKQQEYRLSDNECAYLCGSL

FGAGSDTSASAINIMIMAAAVFPEAQKIVQEELDRVIGRDRLPTFDDQDDLPVTWAFIRE

SYRWRPVSSGGFQHKTTEDIHWKGFFIPAGTSILGNHWGIHRDPLVYPDPETFNIHRWLV

IDDAGNHSLNKDIRHFQFGFGRRVCPGQHIADRSVFINTSNLLWSFNIAQSKDKPIDTLA

FTNAANSHPLPYEVRFIPRFEGVQEVLNVEQTAAEL*

>CYP5703A1(324614) Naganishia vishniacii v1.0

MDSLLSPNHLILYASLALAYTLLKCIYIAPLTSPLAPIRMPPGGHGMLGHQFAVLDGGSV

ATNIPRWTAELGKTFRTQAFCGLYHRLWTIDHKALHHVLRHTEMYEKPNMLRKTLGRYLK

SGLINMEGPRHRLVRKICQPLFSQQNIRNMMPTFLEKGDQLCQLWQDHLASAEQPGSGGA

KNPPAVLMDVLDSLNRLSFDIIGMTAFEHDFDALQDMRCESIAYDWASGLPNGGGGGGGA

EKERGKKPGEGGRIYEDYDEMFGPYEGRAGLRGMLHVVFPILDKWFPTENSNRVRRGMDN

IDHLTSRLTRAKRVQLVELEESGETEGQGVLGRDEKRSKGRDLLSFIVKADMANDPALRM

TDDEIAGALATFMFAGTDTGSIIMAWCLFHLAGSPTLQARLREECLPYGDNISMEDLDGL

SFLDMVVKEVYRLNPSIPTTIREAKKDDVIPLADPIKLACGKFVSEVRIRKGQYVYIPIE

ALNWAPLVWGADCMQFNPDRWSDKRISASSGLGIDYDVLTFLDGPRKCLGYRMGTLEVKT

ALFTLIRHFEFSRDPQIQVRSWNMFTNRPFALDKRFKSFSNMPILIRQYRNIA*

>CYP5139T1(342166) Naganishia vishniacii v1.0

MATLDTLLRQPLPHFSTLQALAGFLGVGVLAFLYRYVWVPYTLPYKNFRGPLSESWIFGV

FPAVLKANPGEAHGKWMKEYGFTMWYKGLFGAPRIWTADPSVIQHVLQHAYDYPKDESTR

GFLAQILGPGVLVAEGADHKRQRKIMNPSFAPNAIRDNHTPIFLNKAYELRDTFRRIIAD

AENSTGSSDYLDVAYWLGKCTMDVIGLAGFDYDFNSLAAPDNELANAFHDMFASDQAQSV

MSILQFVIPPFRLIPTRRMRTQRRALAMQQRIGTALIEKKKQAVISGDGEEQGAKDLLSL

IVRANMDPDLKANQRMSDTEVLGQITTFFLAGQETSSNALTWCLWQLAQHHDVQDKLRRE

VSAVDDDYPGMDTLNSLPYLDATVHEILRLMAPVPSTRRDSRVDATIPLAKPVKGRDGQM

IEEIYIPKNTAILISILNLNTTEDVWGSDAGVFRPERWIEDKEQMSKANAIPGVWGNMMT

FLGGPRACIGFRFALIEIKAILFVLIRNFAFDLPTPKVEIEMKTSLVTRPFVKGEREKGP

QMPLAVRVVS*

>CYP5700B1(384861)Cryptococcus curvatus

MSASALVDTLLSHSTLTKVVLAVLTLLLAVVIELARRRATYAHLPPGPLPHLLTGNDYPA

TYPWRAFYELSKTYGPVITLWTGTQPSVILNDMASATWFLDKHSRDTSDRPPHWAYISGG

KRVILQPHGERWRRMRRALYSILQPAQATQLRGYQERVARGVVLDILGGGEFQDHIRTYA

ATISVHMAYGRMERARYSDPDIAKIVENSARFGNFLRPGGSKLDAFPWLAYVPGYMRQMN

RWNAEELVLFRSALDDVKARSVTGPERCFATYLLERKKELELSYDEVAYLCGSVFGAGSD

ATSAAIQIIVMAAATHPEWQARVQAELDQATAGQPPGFDDLAVDAVPHLHAFVAESYRWR

PVSAGGFIHRTTAPVMYGDYVVPAGVLISGNHWAIHRDTSYFGADVETFNPSRWIKDGKF

NEAMKHVQFGFGRRVCPGHHVAHNSVLINAALLLWAFNMTEQTNSKGKLIPIDTLAFTNT

ANSHPLKFNVSFHERVANLAELMSVDNI*

>CYP5880A1(290852)(Cryptococcus terricola)

MHLPPYSPLLADIIWHNIIIPILCGVCGYLLTAYLVIIPSSTAVIYHVSSWVLGYLAHYV

VSVAHRSSTSHKIAKLADTGNLSGLTIPIVPSEYPFTLDILLQWMNEGRTEYLGDGMIVR

LSKRFGKVNGRRIKTFNTKVLGENSIITFSAHHAHQILSLPLATKVFEKGSKFRERAYSL

LGDGVFASDGNVWRLHRQMSRPFFAALAVRSSVMNYKTDDEEEQSENDGINIDEWRDHET

PARDRIDTHVIRLMRCMMVYEKEEKGFDIQAMFSDFTMEMTIDLVTGGNGQDQIGEDKGI

RPSIQEFAVAFSEAQSILSTRIRIGTLWSLFELTGDKMKSPMKVVRGFVGPLVDEGMKNK

GRGRGSLLDHLVNMTDDRKFIEDEVINILIAARDTTTSALSSCVYCLAIYPEMQDKLMRL

IHQSSTNPQSGMKIPYMKAFIHEVLRLYPPVPLNIRRSNAVAILHPEHPDDSPLIFPPNT

SVILSFALIQRDKDVWGDDAEHFKPERWLDSEGKFKIAIGAKKGFQAFNLGPRTCLGQGF

ALQSVAHVLMRLLSHASENDCQLLLAPELQPEGTKPPAEWQKLTGRGQEEKCWMTSDMVL

NYKGGLWVRWHGKFSNN*

>CYP5699B1(558169)Cryptococcus werringae

MNLPPYSPILLKASNRNLLIPTILVHLVLPSCSLSLRLIIHLITPTLVYVCYTTTLRWLH

ARQAHLLGAQLIPVIKSRTDAPWYLKWGNIDLMLAWDREGKSGYLGEGMVRSGVEGIGGT

VNTRILGEDSMITTSPRHILQILTTSHSSFPKGTKFHERAKDFMGPKGVFVSDKEDWVFH

RSSTRPFFAHTTTLNPAEVFEPSTTRFISLLHQPPSSSSGGNKEHRAIDAQALALIYTMD

VASSFLFGQNVDGLVRLEDAGKRDEEKQRAEEGVGREELARAFSKAQAVLARRIKMGTPW

ALFEMTGNQMTRPVQTIKRFVRPLVDKAIEQDQDGGKAKGEKGKGVLLDHLVKVMKDRDA

IEDELINILVAARDTTASALTFCIYCLALYPEIAERLREEVCSSPAELVARDLKVMKLLN

AFVLEVLRLYPPVPMNIRRSSEAVLLQGSDADSPPLYMPKETSIILAILLMQRSKEVWGK

DAEEFKPERWLTTDEDDQSQVSEARRRREGFVAFNAGPRTCLGQNFALMQLSYVMAKLLR

SLSDPETKSARYKVVLAPDEQPKGTSPPEAWKDLSGRGKEEKVWVASNLTLDVKGGLWVR

IVESG*

>CYP5139AL1(2740583105)Cryptococcus amylolentus CBS 6273

MDRFATLAHLIASLPPLQLTAVSLAILTFAGYLWLYPIAYARLAYRNLPGPDPGSFFWGN

ITGVFAPPTPNSAHADWHKAHGHTIKYRMQFGSHDLSTIDPAALSYIVNNPYIFDKPPVV

KAFLGRLVGAGVILSTGDHHRNQRKIMGPAFGQGAVKEMFPVFYDVSYALKDKLRDLISS

QGRIVTSPSPIKPSELVAGGAKIDVLKYLTLAAFDIIGISGFGFVFNSLSGEKNMVVDLA

EAFLQSGSELGVQYILKQYFPFTLLVSVNQNVCGKGRSLWRGARAGTSLTRKRIQEILDI

TSGEGVQKRQDIGKDLLSLLVKSNMASDLKENERLAEDDIADQILTILLAGGETTSSAAS

ATLRFLTQYPDVQDRLRKELLTIDEDRPSTETLNKLPYLEAVIREAIRLAPGVSLYFRTV

VHDVIIPFSQPLVGADGKLMTEVKMKRGDQLFLPLAAINTSSALWGPDGESFNPSRWLQD

DPNKVKLPGVYGNNFTFVGGTRYCIGYRYALQQIKVVLFVLLRGFDFQELKSKPELFRAT

SSVVQTYIRGEEHLGTQNPLMVVPLGGSAEAPAA

>CYP61A1(576873)Kockovaella imperatae NRRL Y-17943 v1.0

MIPLPTSLHFRNGGVWGLESLSKTRFNISFDNRTLAATIFTLIISLLVLEQAVYRAKKGN

LPGDKWTIPVIGKFTDSLHPSLVKYKKQWYSGALSAVSVFNIFIVLGSSNDMARKILNSP

TYAEPCLVHAAKKVLLPENWVFLHGKPHADYRKSLNVLFTRKALSMYLPVQERIYHDYFN

KWMSDPSPAHPYMRKMRDLNMETSLSVFLGPYLTQAQKQEINDKYWLITLALELVNFPFA

FPGTKVYNAIKARELVMKYLRAASAASKIRMEDPNAQSECLLDEWTKAMIQSRRGANGDG

EEAKLLSREYSDHEIGMVVLSFLFASQDAMSSAIVYAFQLVADHPEVLAKIREEQYRVRG

NDVDAPLTLDLLDDMVYTQATIKEVMRLRPSVIMVPYMTTKKFPITKDYTVPKGTMLIPV

FWNSLHDETCYPEPDSFNPDRWLKNEDGSEPLAESKPQNFIVFGSGPHKCIGGAYANMHL

VATLGTASILMDWKHERTPDSDEIQVIAGIFPKDDLLMQFTPRAPPS*

>CYP51F1(627561)Kockovaella imperatae NRRL Y-17943 v1.0

MDVFNNPFVANMMMNTGRGFNRQPTEAQREFARNFREGKIQPIDPTIPLYILSSIAILLF

TVVGSNLMQQFLAPRDPSHPPVVLHYFPWFGSAYSYGTDPYKFLFKCRAKYGDVFTFIMF

GRKMTVALGPKGNNLSLGGKVSHVSAEDAYTHLTTPVFGKGVVYDCPNEMLMQQKKFIKH

GLTPQAFQTYAPLIWQETHQFFEQEKGFNVYPGPKSYEAIKFFSELIILTASRTLQGKEV

RAGLTSSAAKHFEHLDKGFTPINFLFPNLPLPSYRKRDKAQKAMSDFYLEIMRKRREGES

DVDEHDMIAALTGNEYKDGTPLTDRDVAHMMIAILMAGQHTSSATSSWTLLHLAQKPLIF

KALFEEHRDLFQNEDGTWEDVTYESTKEMPLMAAVIRETLRMHAPIHSIYRKVLQDLEVP

PALAAGGNGSRPYVVPKGHFVVAAPGVSAMDPKVWPDADKWDPFRWLEDKGMAQEALDSY

SGATSEQIDYGFGQVSKGTESPYMPFGAGRHRCVGEQFAYLQLSVIMSYIVRNYDIQLLG

AFPQTNYNTMIVLPLNGYVTFKKRGQSKWILSTK*

>CYP5892A1(633869)Kockovaella imperatae NRRL Y-17943 v1.0

MSWFRRAPPDPKLPPGDLGRSVAGNTFTFHDDAQSLILDNIDRFPGSPVYRAKVIGKDVA

LVTDYRLAEKVMRTHEPTDDGPRFSQRAAYSDLMAPFFIEPNVLLEDQCEETAYANRDIW

NRHVSTLLDSQDWEPINERIKQIIVTYRDKWLQMDAFDVYEECKDMAHELVLYLFLGIDK

ASADWDSVVELSTTSLRGQFSMPVKANFGSMFRSSYSRGLQAQEELRIVAEDKLQAGQCP

FIQSRTPSLLNSAITHTAMFASSLVIKAVASYLTFSLIQLSRTETRQADLDKVLRETERL

CPPVIGALRRVMDHPWVLTGDKEYEIPIGWEAWVYFPLANRDAKVYGEDAWEFKPGRWTD

STPPSLTFGYGEKSCLGMGMVRRIAKSVLAVLLGDDDRNHGELELISTLDPSLKDFLGWE

RHVGGWQGIKQLPVQKPRDKVSMRYRRQ*

>CYP5891A1(650743)Kockovaella imperatae NRRL Y-17943 v1.0

MSLYPALSSMASQLTWTVILILASYLLVRRLSQSRVKYRDGLPIPPLVGNWIPFLGNAID

IARGDNFWRERMSAYGPVFRVNAMGSEKIYTVDPSIISWVYRNSKTCDFPWYRRHMSNHV

YGMSRDVAFSPVWDEHLFAMHHRAMSPGMIGTLSERYVDELEVLMSAWTKDVEALGNGQG

TLVDLADSVFDIMYIASSKAFFSSSFPAKESIPAFKAFDQSIPLLLLETLPSLLMSPARR

TRTDLNKIIMDWWKTIREDLKAREALAPSLIGMLDTGEEEGWSDDDLVSCALPELWALEA

NAPYAAVWTIVEIVRRPALYDLLYEEITQAIHSLSPSHLSTLIHSTQPELLAKCPGLNAA

FQETLRLHTSSFSIRKVMEPLVIPGNLITSGPGKGVGYSVAKDDDIVCVTRHGHVSTDME

WGVTADIWDHTRHLNANAKTAQMWPFGGGTSMCEGRHFASIEILTFLISFIHTFDVQVVS

DNMQVDMTRVGLGIIQPKGKTIVRLSRR*

>CYP5893A2(155052)Kockovaella imperatae NRRL Y-17943 v1.0

MSAFGLYRSSFGELFPVIAALDIAVAGLLLGVAWILRARVLTPYNHLPCERCNFHPSFTR

TDHSSAPPGLSLKHDHSSLSLGKPYGSTWREWFKTYGSTMLIRVAPYYRPVILTKDPSFI

RHVFANINDFEGSPRFSKAFKLMGLNGALPGVHGERRQRIRAVYGRSASLAAMRAKFPQM

LKVANKVKQEISQASEVVNGQPFNPAPHVIAFQMGILSKIVFGFTDEEIVNGKPKEFQDI

LAEYLKSRQSLRWWDLHTHRILPSGRWRKHQHNRRCIANFGKRVLREKQAKITGAFGHDL

KVEEIDEMGDDIITSWCRYNMANDIPENQKISDEDLVDNVPLAIIATMDVSRLIDTEILH

RLAISPDIQARLRDELSALPDEPSLEDLSKVKYLEAFIEEIVRINNPYTTIERTCTRTSV

VPLRDPIQMSNGKTVDSITVHAGTDVSVAVYQVAQDESLYGSDPEAFRPERHLGGSCEKV

DGGVALHAAWGSSVGFSGGGRGCPVYQFPLAFMRAFIFVMVRSFEISEPKDQNVEVWTHT

VADIRQWKINGKVATYIPTIFTPIIQDE*

>CYP5139B6(618988)Kockovaella imperatae NRRL Y-17943 v1.0

MDHLSELATAAIEFARHRPVLTIIGIIITSLASLWFYYWPWAEWTLSFRNVPGPPSDNWL

FGNLRAIIKAGPMELHREWAKEYGPTYRYRIIFAQHRFYTLDPKAIAHVLSHPDTFPKPD

RARQQLAKTLGNGLLTAEGADHRRQRKALNPSFSPLAVKNMQDIFFDKAYELREKLVDII

ADDTIQASPTPAKPEDTVSGGKKIDVAKYLGQTTLDVIGLAGFNYDFQSLSQKDNELAEA

YRSMFAASSQLTLMLFLAAFVPGFDKIPTKQQRTVRASQEVTRRIGNRLLEEKKRAVKAA

YSDGFEKGSDIGKDLLSILVKANMASDVPPDQRMSDDEVLAQITTFMLAGNETSSTSLTW

ILYLLCQHPEIQTKLREELAAVESERPSHEEIAALPYLDAVVREGLRLLPPATSTIREAK

QTTVVPLGTPIRGRDGKMMDTVTLQAGTTVFLPIMPVNTSQDRWGSDAEDFNPDRFLSPP

PESTKEVPGVWGNMLTFLGGTRSCIGYRFALAEMKAILFVLMRNFEFEELKSKPQIEKRS

ACVSLSFHGADGVIRIVMRSKVVGEETQGYQMPLMVKPISA*

>CYP5888A1(649398)Kockovaella imperatae NRRL Y-17943 v1.0

MDTVFGMARWAIAQPRQGWMGVSALAIVLVFLAIVSAGLFLFLRGALVPFRDLPGPAPDH

WFYGSFKTLMASPSGAMVDKWTKQYGTTFRWTSIFRRPEIISLDPAVASYVWSHPDTFDR

AQSSLTQFKELIGPSVLSSAGDEHKRMRRALLPAFSVAQMRNNLPIFWKKAYQFKDKLAQ

QIGEHGKDINPVKLLDQLTIDIIGRAGFNHEFNSLGDGQDELLKAFQRRQVANTVSGMVR

MLQFTGVPVGRLFPNKTQRESQEAKRLSYKLSKEIYDRSVQAVAGDTDAKISKGDIVESD

LISVLIKSNMASDVRSEQKMSAEEIIGNMFAFILAGNVTTSRTLVWALHVLSTRQDVQDN

LRTELSRVPLDELELDELNELPYLEAFVRELLRLYTLVPMNVRQATKATTISLSRPIKGR

SGKTIDTLRINKGTTVLLPLLNINTSTEFFGQDADTFRPERFLDRSKEGQSWSSVPGIYG

NITTFTNGSHACLGYRFAMIEIKVVIFVLLRGFTINAVPGQSDVVRQGAIGISVPVVKGA

KVPGLSFNIRPNVK*

>CYP5890A1(651333)Kockovaella imperatae NRRL Y-17943 v1.0

MSSSTLADALASLTSLTTATALATILSGLAVLWMSREAKQRFWFSFSNLPGPRADSFMRG

SFPSLLAKETGQTTREWFDKYGPTIQWRSLLYTPNILTCDLGLASYVLSHDDEFGRSKSH

EADFRRLLGDRSLMTVDRSDHKRLRKIMAPAFGVGPMSDQWPIFLHQARRLVDKVGPEGR

QIGASSFMGMFTTDVMGMASSGTNLGAMDGAPNELYTAWMGILQTTRLRGVLWQIKTKLA

IPGTWLHSERVLGKSKDTFQRIGDDIVQTRKRELLGEGHEVTRSDFGSKNVISTLLKANM

AQDVKDSSRMSDEELVSLLSTLILASTVTTTSTLIWCLSLLATRPETQARLRAEVMDMED

EPTMNDISKLPFLEAFVRELLRLNSAAPTITRHTHKTVTVPLSTPIVGKNGSLIHAVTID

KGTDINIAVPSVNTSKEIYGPDAEEFRPERFMDGAVKATGLPSIWGQSLTFSAGTRPCLG

FRFAIMQVKATVFALLRSFEVSQPEGQEVVRMGMIASSKTIVKGTNKSDIPIIFKPLSS*

>CYP5889A1(622196)Kockovaella imperatae NRRL Y-17943 v1.0

MQLVLSLLLATILVLLYFWRPKTRRQGIKLQGPQGKGLLSTAPYGQLQSEWFDTYGHTLA

YRNDSDKLSILTCDPAAVAFIHREHDSILQPASIKSFALKNMGPSLMGVEGAAHRRQRRV

IAPAFGSKAIRAMTPTFLSMAIELKNKLLSTAGQEIDVTTSIHQLTVDAIGSVAFGCEFG

ALAGGGHELLDVFYRMTSAAQPSEKDQAIEAAHVTMHRIGSTLVKQRTEQILAETAGGPL

DKKSISANDVLTNLLRANMATDVAEDQRLSDEEVAAQIPLFLFAGNTTVAVTLDWASALL

ARYPDCSAQLFAETDEIGSDNPSWCVAFYLHRSRQGHHQRFAIPRRVCQGGAETLRSSPN

FVPGSSKRHTTSVGAPCGGSERANSHIGPRPCWYHHPHR*

>CYP5893A1(680485)Kockovaella imperatae NRRL Y-17943 v1.0

MSLIAYYTSLLGNISPGLAGIICLASSTLLILIGWVVRTRILTPYRHLPSPPGLSLKHNH

SSLTYSTPFGQKWGDWFKTYGNTIVVRVAPYHREEILTIDPGFVRYVHANVNDFEGSPRL

GRAMVDMGIHGAISCAKGEKRQKIRAVFERSCSLAAMRGYFPRFINLAYKARDKIMEELE

SDTEKRSGKKPINPSTHAVGYQMGIVSTVLFGFNEEDIAKGRSTEVRDTIMAFHKARSEL

KWWDTKSLFRTPRYRRYNRSRDQLAVVGKKLLRDKQEKITRAFGQDLKSDDILEAGDDII

STWCRLNMSKDIPASQKISDQDLADNVPIAMAATLDVSRMMIYEIIHRLASHRDIQDKLR

KEFSALPEEPSLDDLAKVAYLDAFLDELVRIDNAYTTIERTCTRTCVVPLHDPITLSNGQ

TVNSITVHKGTDVSIPIWNLSADERLYGSDATEFRPERHLDGSTKGAEGGTAIHATWGSS

AGFSGGQRGCPVYQYPLAFMRAFIFVMVRSFEISEPTDQDVKIWVSSSAERREHRVNNKP

VTWIPTIFKPISQDV*

>CYP61A1(566522)Naematella encephela UCDFST 68-887.2 v1.0

MNKFGASHTILRPTSVDLTTSKAWGLDGLSKAKFTFDGKTTAATVLTLIISLLIIEQIVY

RTKKSYLPGDKWTIPIIGKFADSLNPTLSNYKSQWNSGPLSAVSVFNIFIVIGSSNEMAR

KILNSPNHAEPCLVQSAPKVLLPENWVFLHGKPHADYRKALNVLFTKQALSTYLPIQEKI

YREYFQKWMNDPAPHKPYMMEMRDLNMETSLSVFIGPYVTEEQKKEINVKYWLITLSLEL

VNFPFAFPGTKIYNAIQARKLVMRYLTSASAQSKKRMADPSNEPECLLDHWTRAMVQSRA

GGNVDGDGEQARLLSREYSDHEIGMVVLSFLFASQDAMSSAIVYAFQLAADNPDKLAKIR

EEQYRIRGNDLSAPLTLDLLDDMVYTRATIKEVLRLYPPVIMVPYTAKKPFPVSPDYTVP

KGSMIIPAFWNSLHDETVYPEPDSFQPERWLPQPDGSEPLADGSPQNYLVWGSGPHKCIG

GQYASMHLAAALGTASVLMDWDHERTALSDEVKVIAAIFPKDEFRVKFTPRAPPA*

>CYP5891B1(525767)Naematella encephela UCDFST 68-887.2 v1.0

MFFSFLLLLPLALVLRRLFYHLRPTETYGGVQRPPFLPDWIPWVGVALSMAGGDSFFCNV

QKKYGPCVRLTAMGDTRIFITSPALITYIYKNPKAFVFEYKRRSIQSLVFGMTHTVAFSQ

AMSDRLFPDHHRALQLVNIGELIQKYTTQLEELVLSKVDEADGAEVDLFEMVYEIIYTAS

AKAFFTPSFPARTTFKPFIAFDRAFPILNLARLPITMRSQAISAREKILTMLEEWWETTT

ELDRAAMAPAVCVQWEIAKDEGWSKRDWATVLLAETWALEANAPFAGLWTVIEVLRRPQV

LASLRAEVSSTISKLDPPEISTLMHLSQPELITLLPLLNLALQETLRVHSSSFSIRVAEY

DTTFPAAVCEGRELSVKKGEEIICVTRPGNIDTTGAWGTDAEIWDTERWIGDDADDEKGK

ARGPMFPFGGGSSMCEGRHFASAEILSFLAIFIATIDVQVVRDTIKADFTRIGLGVMQPK

GELRVKMRRRQGESKVAA*

>CYP5767A2(152747)Naematella encephela UCDFST 68-887.2 v1.0

MSSLTWGQYLYIGGVFVAAIVLHRLRKRASLLRIPGPRSANWLLGNEWEIYNNVAGRVWN

RWIDLYGPVVLYKNAFFNGYTVLLADPIAVKSLLSDGPDGYTWHKPPFNRQTNERLLGRG

VIWAEGDDHRVQRRLLSPAFSPTSTRLVSDAFWETADNLKSVLQRQIDLDPARTARVNAA

DLMSRAALDVIGLAGFEHKFHAVLSASREAVSITDGLHQALGAPPGWRMFLTLNLAKSFP

FIFNHAPLKAIQNQRASKASIDIVAYKVLADAKKSLLEKDGKSILSILLQESQKGGEKLS

EKEIVDNFSTMITAGHETTGITLSLILYELSRNQPFQDLLRKELFAVGGGQLSFDDLQSG

KATHLDAVIKEVLRVYPANIRIIRRAETDQVLPLSQPLPSAKERAGDTQLLIPANTDVVI

PLAAINTLKSIWGPDAHLFKPERWLEKDGIPETVKVLPQGYERIFTFVSGPRACLGMRFA

LAEMRVLLSQILSSYIFTPIDDANIPLDIVSPAQIMIRAQDPTRGIYGVPLRVREVKN*

>CYP51F1(523016) Naematella encephela UCDFST 68-887.2 v1.0

MTVALGPKGNNLSLGGKVSQVSAEDAYTHLTTPVFGKGVVYDCPNDLLMQQKKFIKHGLT

TEALSSYAPLMYKEATDFFEHELGLTPATPGPKKLEALKIMSELIILTAGRSLQGKEVRA

SLNARFASLYEDLDGGFTPLNFMFPNLPLPSYRRRDRAQKEMSDFYLDIIRKRREGETEP

EHDMISVLSGCVYRDGTPLTDRDVSHMMIAILMAGQHTSSATSSWILLHLAHRPDVVEAL

YEEQKKLFGNQDGTFREVTYESTKQMTLMEAIIRETLRLHAPIHSIYRKVLSDIPVPASL

SSPSESSAYVIPEGHFIVAAPGVSQMDPLIWPDAKTWNPYRWLDEKGVAASAKEQYSGAT

SEQVDYGFGQVSKGTESPYQPFGAGRHR*

>CYP5216A4(552749)Naematella encephela UCDFST 68-887.2 v1.0

MKGAAFLLPALFRILVLPILIVQPILRFLPDLTLFRLVAYLLSFALVYAVRGRLDVWSTS

RQASAQGAADVPLVNGRWPFNLDIVRDWAKSGSEDEVARMMFLLGRQYGSTYNTRVIGEN

QILSTDPKVFKHALVDDSNNFVKGKKFKDRAEGFLGDGIFNSDGERWKFHRTLLRPFFHP

SHVSHKLFIPFIQRFLNNLPSGEEACDIQARFRQLALETALMWMTGTDIGDPSSKGSAEW

EQSKNDLGWALVEAQRVVGRRVKIGTVWPLLELGYDPLARPMQVIRTFFAPLITAAVSRR

DQQVQDSLRAGDEVHLIDRLIEATEDMKLVEDQLINVLLASRDTTSSLLTFCTYALALYP

DVADRLRGEVRSVVIGDVNKGAIRECRLCRGFINETLRLFPPVPLNIRRTLRPSLLRTSA

AGPPLFMPANTSIILAFILMQRDQAVWGENALDFDIDRWLKGGLDIKESEGFMSWNAGPR

MCLGQPFALTLVHTFLILLFRHLDSLGSDRGENESSGSFQLQLAVDAQPAETRMPESWKG

KEGDGRARDGRDRVWIMGDVILTVKGGLWVRLAQAAKL*

>CYP5894A1(553062)Naematella encephela UCDFST 68-887.2 v1.0

MVAVYLAFAVVILWFGRRWILSLLTPRSIPGIPAYPDPVPVLGDIPRLAASMKATNGFIG

FMDKVAKDLGPIAQVRISFLNTMVVVTDYQSIDNILTKQTQHCDRDSHTIKAFRTILPRA

LISLPTNDMWKHHRRIIGTAMTSKYLSLTTPRANESVKELVNLWRRKASVCEGRAWAAEG

DMESAALDAICGMAFGHSWGIIPSYSSQLVEGEYPTGINGEVVFDTVRPELAESTWAIFN

SLPTTSSFPYILHKIRTLSPTYRRHARRLDRFLDAKLADSRARAESVGAEVAVDVADNTL

DLMTARQLRGDDWMPDDEMKDEVFQYLVAGTETSGCTLAWWLKYMTNHPEVQRKLRKHML

EKLPEMQDRPPKFEDLNATNTPYLEAVVHETLRLSRTAGGYSREVTQDVTVLGKVIPKGC

TIIIPTVTGLEDRSTPVYGPDPNALGSDSVDPDDIAERMDASLEVLRPGSAKRKVGFWKP

GSGFNFDPERWLNEQGHFDINAGPSLPFSAGQRGCFGKNLAMLELRFFISQLNQTFFFAP

VSDALNSFDMYQTVTTHPRQCFIRPVSWDSDL*

>CYP5393B1(585694)Naematella encephela UCDFST 68-887.2 v1.0

MEQLNEHLTRSNIYALVGLLAATYFGNWILGYIHVHWQVRGLPKIHSGIEYFESGLRGQL

PHIPFIVPVKQYTLDKPWKKYADVGCDLIALTQVTTSYPVYISSNPAVAQQISQAPMRFN

KAVHDFRYRALDIFGKQIVSTSSGPEHKRHKAVVRGCFGEEVMENAWNKIVDALQIMLRE

EGVEDGGVLKGVRDVMVKTTLLVFGKSGFGIDIPWHIPHTNGSILPFPEALHSVEDSLAA

QLLLPQWFMTYSPSSYLRRCAKAQRSLVFHIKQMIASRQAKLTAEHAALDKNEKVKPPTD

LLGALVASQIDVQRDQGMEKGLSESEIVGNIFIFSIAGHETTAYNLTWALAFLALYPDVQ

EKLYEEVNSVTGGELPTYRDVKALPLCLATIYEALRLRDIVMTLTKVATEDTLLPYTKWD

PSSPTPSTVTHHTHTIKKGSYIVTDTPAAELNPFFWTDPHKFDPTRHLGEPQGPFVGFSM

GTRQCIGKRFAEVEQVAFISHFVKHFKILLIQQPGETWEMTKERMLHATQELTYTPANFD

LKLEKRI*

>CYP5889B1(460805)Naematella encephela UCDFST 68-887.2 v1.0

LVGPSSGHILYGYQQELLRSPHGSLQSAWFARYGPTLRYRGMFRQSVILTMDPTAVRYVH

QHSEIFLQPQSTIKFAQENQGPSLIGVEGPDHKRQRRAIAPSFGPAQIRHMTPLLLDKAY

ELSNKLAGMIGQVDMVKVIHQLTVDVIGLAAFDYDFGAMHSDGNELLDAWKRMMMGNHES

RLVAMLQRAGVPLARFKKTERSESLEKASDMMKRIGQRLIEDKRRSLQAEIGEDIKKADL

VEGDLITNILRANVASDVRDNQKMSDEEVLAQIPLFLFAGNTTVAVTLTWAVGLLAKYPA

IAAALRQEVRAVDSFRPDWDTLDSLPYLDAFVRELLRVHPAAPTLFRVASTATTVPLGQA

VRGRDGCLMDHITIQRGDTIHIDYGRMNLDPNIWGPDAAIFDPERFLQKDYQTEAIQTFG

GGARHCIGARFAVAEIKATLFVLARELTFGEIESRPQFTKFGGYVAHVFYCLHIKPRLGP

TESWRKGGNAAHSLQDAMKAIVDRPCLPSMMVRYRAGSSRATAECQMGKTAIFSCTQALR

GRLVGT*

>CYP5889C1(512990)Naematella encephela UCDFST 68-887.2 v1.0

MTMMKLSLSQLTNVIHNVSWSCIWLYNLSVICMGLVVIIASIVWREWTIAHYNLQGPPST

HWFYGNLQALLNAPTGTLQARWISEYGPVFAFTSIFRRPEIMITDPHTIGYIYNHPERFD

RAISAKKILRYLTGHSVIAVDEPDHRRMRKAILPSFAPAHIKELMPIFFQKTYELKDRLL

DRLSDDRAEVDATKYISETTVDIVGLGGFSYDFEALSSEGNELLQAWTKLRDGIMTRNWV

SMLQFAGVPGANWFGYFTKRQKGARAARDAMFSVGERLVQEKKRLVLAETRGEIKKVDVQ

GMDLLTVLVRANMASDVRSEQHLSDDELISQIPLFLLAGNATTTMALTWSLVVLAKNAQI

QDELRADCMSLANEQPSLDQLNSLPYLEAFIRELLRVYPSTPAQVRVVTKDEVLPLSTPV

RSREGRMMDVVHVKKGTGLTLALFNVHMSTELFGPDAAEFRPERWLDPGMNEKVKKIPGM

WANLLTFGGGDRSCIGFRFAIAEMKAVLFTLIRHMAFEETEEKPEVVRVGGLGISRPVIP

GRDDIPIVIRRLA*

>CYP5139B7(520702)Naematella encephela UCDFST 68-887.2 v1.0

MSFLSSFPVFPVYGSVITTYLAGVALALFGLWFYFWPYGEWTLPFRNVQGPESSSFLWGV

LAQVIKEEPMKPHARWLEAYGPTIRYRVFFGKHRLLSIDPTAISYMLSHPDLFPKPERTR

HEMAKILGEGVLIAEGDDHRRQRKLLNPSFSASAIRGMVPIFYDKAYELRDKLQAIIDGD

SNVLPSPTPAREGDEEVKGGKKIDVMQFLGQATLDVIGVAGFDYDFKALSEPKNELAEAY

RQMFTSGQSITPMAIFQAFVPGMDKIPTERVRTVSRSKAITERIGRKLLRDKKDAVRKAH

ADGLEKGDDIGKDLLSILVRANMASDLKPDQRLTDDEVLAQITTFMLAGNETSSTALTWI

LYLLSQNPQVQKKIREECASVADERPSIESLQALPYMDAVVRESLRVCPPAPGTIRQTTA

DCIVPLSIPIKGRDGSTITSVHLPKGMQVFLPIMLVNTSTVIWGPDAAEFKPERHLAPLE

GVRDVPGTWGNLLTFLGGARNCIGYRFALAEIKAILFVLIRNFEFEELNSKPVVERKASI

VMRPRIVGEEAAGLQMPLMVKVLST*

>CYP61A1(336)Trichosporon asahii var. asahii CBS 2479

MATHASETIPRASALPLNFDFVKAWGVENLSKARFQFDTKTTTATILTVIISLLLLEQVV

YRQKKKHLAGDKWTIPIIGKFADSLNPTLANYKKQWYSGALSCVSVFNIFIVIGSTNDMA

RKILNSPTYAEPCLVASAKKVLDPTNWVFLHGKVHADYRKALNVLFTQKALSIYLPIQDR

IYRRFFDEWCAENKTQEMMLKMRDLNMETSLSVFIGPYVNEQEKQEINKLYWDITVALEL

VNFPLAIPGTKVYNAIQARKVVMKYLMRASAQSKVRMADMDNEPECLLDEWIRAMILSKN

GNSEQAKLLSREYSDKEIGMVVLSFLFASQDAMSSAIVWTFQLTADHPEVLEKIREESMR

VRGGDVDAPLSLDLVEDMVYTRAVIKEVLRLMPPVIMVPYLTTREFPVTPDYTVPKGSMI

IPSFWRSLHDETCYPEPDAFKPERWLENDDGSLPIAESKPQNYLVWGSGPHKCIGVQYAS

MHLAATISSAAVLMDWEHERTPDSDEVQVIAAIFPRDHCRLRFSKRAVA*

>CYP51F1(1742)Trichosporon asahii var. asahii CBS 2479

MSIVQGAIEAALALPLAQKLAAVFIGLPVLAIVINVLSQLLLPKDPTLPPVVFHVIPWFG

SAAAYGMDPYKFLFDCREKYGDVFTFILLGRRMTVALGPKGNNLSLGGKITHVSAEEAYT

HLTTPVFGKGVVYDCPNDMLMQQKKFSRSRVCCFCRSASGSPMPRSTPARTFRRNPTNAQ

IKHGLTTEALSSYAELMRGETRQYFRDHVVTGKPFEVLEMMQQLIILTASRTLQGKEVRE

NLDIRFAKLLEDLDKGFTPVNFLFPNLPLPSYKRRDKAQKEMSDFYMSIIEKRRSGEHDH

ENDMIAALQGSVYKNGVPLSDRDISHIMIAILMAGSHTSSATSSWFLLHLAYDQELQQAL

YDEQVQLFGNADGSFREMTLEDTRELPLMTACIRETLRLHAPIHSIYRKVMLDIAVPPSL

AAPSKDGTYVIPKGHFIVAAPGVSQMDPRIWKDAKTWRPRRWLEEGGVANAANEEYTSGE

RVDYGFGAVSKGTESPYQPFGAGRHRCVGEQFAYLQLTMLVSEIIRTFKVEPGAPQFPET

NYQTMIVLPLHGMIKLEPRK*

>CYP504C3(5567)Trichosporon asahii var. asahii CBS 2479

MAGWSSEALSFDKLGLPAIVAAAVVFVLISEYYRYAKGKLHLPGPMPYPIFGNLPQLGKD

AALTMHKWSKKYGDVFKVTLGEREIMIINSAKATKELLSDQGGVYISRPMFHNYHSTLST

SAGFTIGTSPWDESCKRKRKAAATALNKTYVQEYLPIIDREALALVEDLYVNGKGGALEI

DPAAYCSRYALNTSLSVNYGCRLDSVGDDLLAEIIDIETGVSDIRSTTSSWSDYVPLLRK

LPTIRKTSGGYTTQEIKERRDKYMGLLLDDLKEKIAKGTDAPCITGNILKDPDAKLTPLE

LSSICLSMVSAGLDTLGNTFIWSLGFMAKHPEIWEKAYTELNKISNGNPPDSTEETCEYI

VALYREAARYFSVLKLSLPRATLGDSEYKGVHVPSGTTVFLNAWAVHHDEDRYGDVENFR

PERHLEQKDKEGALAHYSFGAGRRMCAGVHLANREMYVGFAKLIYFFKLEVGSEDYDINP

ETACKNTYGLSSTPFPYKVRFVPRDPEAIEQWIQDEKSRNDISIASAIKSKVNA*

>CYP53A55(6505)Trichosporon asahii var. asahii CBS 2479

MTTSVHQDLSTRLDQLKSLPAGFWLAAALVAFSAYYLYPYFVTYAALRDIPAPFPAQFSD

WWLFRVVKRGNRSVTVDKLHQKLGKFVRLQPNHVSIADDSAIKAIYGHGNGFLKSSFYDA

FVSIRRGLFNTRDRAEHTRKRKVVSHTFSVKSVREFEPYMHDNLNLFVKQWDKLVDNAKK

EGSNGGKARFDCLDWFNYLAFDMIGDLAFGSPFGMLAAGADVAELRSTPDAPAEYAPAIE

ILNRRGEISATLGIVPKLIPYAKYIPDPFFSKGVEAVANLAKIAIAQVRGRLENPPADAR

TDLLARLQEGRDDKGKPIGREELTAEALTQLIAGSDTTSNSSCALLYWVTKTPGVLEKLQ

AELDEAIPPGTDVPTYDMIKGLPYLEAVFNETLRHQSTSGMGLPREIPSDSPGVTICGRY

FPPGTILSVPTFTIHHSKEIWGPDALEFKPERWFNVTQRQKDAFIPFSHGPRSCVGRNVA

EMEMKLIAATWMYRYKPTLLQDKLEVAEGFLRKPLGLEITIDRRH*

>CYP5887A1(7281)Trichosporon asahii var. asahii CBS 2479

MSVAYAVAGVFVAWLLYIFFPAVKSRQWFAHQLNGPPSPSFLTGRFFIDFWQKDHAPHMG

HVQRYGKIFKYPFALGIPMIFIADPYIVSDVYSHPDTYKKTKGMITMLDNLFGRGLLSAD

DDFHKQLRRIIAPAFTPSVIRSLFSEFLEEAYRLQGRLQDKVKENTEVDMLNELSRTTLD

IFGTTGLGQDQLGDPLNRLGQAYTSLIGALTADMGKFGMLQVIITQLQIIPSAMMRMIKT

SRAIIESKGKQMIAQAGQKRDKDLLSLLVKSNSNESASKQLSEQQIIEQITTILFAGHET

VSTSLSWTLYHLADNPSLQTRLHDELSELPEEPTFEQLDACPLLGKVVREILRVYPPVPS

TQRELAVDLEIPLSEPVTLKDGRVITSLPLKKGEVLNLSVICANRDKDVWGSDAEEFNPD

RFDRPNTPAKQIPSAWGNVLSFSHGSRNCIGFRFALAEMKALLFVIMRNWRVERVCPVEH

RNGITSRPYVDNKPSCPLRLHAL*

>CYP5139AW1(7657)Trichosporon asahii var. asahii CBS 2479

MGPTFFDKAEELRDIWLKYATRTEPAGWAEAAPFPPKPEDEAQGGRKIDVMKGVMAMAID

IIGQAGFGYQLRALSTLQGEDNKLVDAFRNMIQGGMPNTRLQMFLEMIPWFRGRTKAERT

VEECAKLAESVLAERLRQSKAEVAAQMAAEKEGIATGPSGTEAKDLLSLMLRSNMNPDIK

VNQRLTDYDIQSQLRTFMLAGNETSASSTCWALLRMAKAPEIQSRLRAECMTLGERPSID

ALDALPFLDAVVREVLRTDAVVQASIRTAQRDTVIKLATPVRGRDGNMITEVLMRRGDSA

FIPMQVMNWDPEVWGPDSLEFNPDRFLRPGIPAKTVPGVYGNIMTFITGRHGCIGWRFAV

TEVKVVLFTLIRAFSFEELPSKPEVVGQLKVILRPAVKGEDEETTYQLPLLVRALDTA*

>CYP5139AV1(378)Trichosporon asahii var. asahii CBS 2479

MTVAGAAAEALDRLPVVGPVLASWPKTTLTALIALAIFVALYAYLYVYRSLTLPFANVPG

PEPSSFFWGSLMEIIKAPPSAMHEQWYAEYGSTFKYRAIGGDWRLCSTDPGFLSWMLGHD

ELFPKPPHSQKFLWKLLGPGLVTVEGDHHARQRRVVSPAFSTKALKDQVPIFFDKAAELR

DKLLLFVEGKGDERPALPPSPGDEEKGGRKIDVMKYLGQMAVDIIGQAGFGYDLASLSGH

PKPLAEAFRVMMGAGLNPGPIQVLKTFIPFLSFIPTRADKILEESNVIAHREAAAIIAER

TREVRAAGDVDEGAFGKDLLSMLLKSNMSSDLRPDQKMTDEDVFAQVTTFMLAGNETSAT

ALTWILWRLAQHQDMQQRLRDECQEVNEGDYSAVNSLPYLEAVVRESLRLDSPVPATLRV

TKEDVTVPLSKPIRGKDGTQMDSVRLKKGTTVMVPIHVVNWAKDNWGADSKTFNPDRWLK

EQSPWKYQNPGVFANLMTFNHGPHNCIGYRFSIAEIKVTLFTLIRALSFELLPSRPKFEM

KTGIVMRPMVVGQEKEGPQMPLLVRALEA*

>CYP55A30(3656)Trichosporon asahii var. asahii CBS 2479

MAFSPKFPFNRASGMEPPAEFATLRATNPVSQVQLYDGSLAWLVTKYEDVIKVATDERLS

KVRTRPGFPELSAGGKAAAQAQPTFVDMDAPDHMRQRGLVESFFTPEYVESLKPYIQRVI

DETLDKMAAKDKPVDFVSEFALIVPSYVIYTILGVPFKDLEFLTHQNAIRTNGSSTAREA

SAAAAGLIDYLGKLVDARMKEPKDDLVSTLCKAVEAGKLDRTSAVQVAFLLLVAGNATMV

NMIALGVATLAKYPSQLELLKADPSLAANMVQELCRYHTASAMALKRVALEDVVIGGKTI

KAGEGIIASNYSGNRDADAFPDPDTFDIRRKFDKDPLAFGWGPHRCIAEHLAKAELTAVF

ETLYKRLPNLKPAVPIEDIEYSPLDKDVGIVSLPVTW*

>CYP5139AU2(6415)Trichosporon asahii var. asahii CBS 2479

MLYLVAAVLVPLALYLYLYPLREARLPYKNLPGPKPVSGFFGSLGELTARPLTSRYSQLK

DNYGTTSRFRALLGKWRIVSTDLTAITHVLRHTGTWHRHETFNVMIDRITGRGVLSVEDE

DHRRQRRILNSAFNGTAVNNMVPMFWEEGRILRDHIDQQLGGDEAKPLDVLQNYTGIALS

IIGRAGFNYDFQSHERDTNPLAVAFNNMINASLENKMAALLQNVFALPFNISPTSHFAQK

KSRSIVDSIGAQIVRNRKAEIERDHISLEKGDYEGKDVISLCLRANMLADQRDRLTDAEV

MGQIGALMLAGNETSANALSWATYHLCQNKDIQNRLREEVMAVPDEPSGEELDAIPYLDQ

FVKELLRFEPPLPQVVRQASEDTVIPLGQPVTGRDGKLMTEVHVSKGTDFVVPINLVNRL

PEIWGPDAETFNPDRWAQKGYPNTYIPGVWGNLLSFIGGPHHCLGFRFALLEIKAVLFVM

VRNYEFDFIPQGPQVMRIAPGIIQRPVVMGNEKAGIQMPVMVKKLSAEVREQAA*

>CYP5139AX1(7286)Trichosporon asahii var. asahii CBS 2479

MTAIGAQGSALEAGRHLLTLIQAHPWLFSLAAFLAWFAARIYVTKYHGPFSKLPGPKRAH

WLIGNGIEVMNLTPKDAHQRWLDGTEGTVKIPRVLGQHYACVTDDLKASQYMWTHPDLFI

KPEGGRKILEDALGLGLVAAEGKLHRRQRRVLNPAFGWPQLVPMAPTIFEKAYELQRKMF

RTLEEPDGGKGVDHVPGKRKLDVLSFVSQATLDIISLVGFGYDCQALGDEPNELREAYNQ

TLATVFNITLMSIVQFALGRVAGWIPTERQRISHAVRAKSQEIGMAIVAEKKAQLLASNN

GVIEKNSQDLGKDCLSLLIKANMAPDLRDDQRLSDAEVADQCSTILFAGHETTGTGLMWT

LYHLACNQDVQEKLRRELLEVDAREPSWDELNALPYLDAVVHESLRLDSPVPMSSRVATA

NAVIPLSKPITLTNGEKINELHVPKGAEVSTPIAYINTSTKIWGPDARSFNPDRFARDGI

PAQQTPGVWGNLMTFLAGPRNCIGHRLTVIEMKIVLFVLIRSFRFEIPPSQPQLKKTWLV

IQRCVVVGEDKAGPQMPLMVSAVEN*

>CYP5886A1(3236)Trichosporon asahii var. asahii CBS 2479

MFLFLACAAAFPLSLPLLYWWLQPPRNFAPGLPVVPLWIAFLPLLRSWLGFPFPGQDETY

ARYVEPLMAQHGAVVIYFGSQWNVLVACPQGMKQLFSLERTTFQKQGNHRKLPNAAISAL

TGGNIISETGALWRKYAQIIRPSLRDDTDPSKVDIAAGRLVAQLEGHAGSVTGLVQRYSM

EAFLPSPPAIHRIHALVKRHIFHPLFLSVPALDRFPRLFPSRAIARNLIGNLEVELLKAL

PGAEARLKLGLPANEKDESGNVQSKLDGAFLSGLLSEREYIDNVKSETGPRPKLTAVLFI

AGHENAQQALLTLLWILSRDPELQARIRSDASPSPILHATVLEALRLYPPIPQLINRRIA

HDTLLSLPGLDVPLKEGTYVGWSAYGLHRSIDPEWRPQRWGESIEESGR*

>CYP5878C1(6509)Trichosporon asahii var. asahii CBS 2479

MIWLLTALAVVAALVYHFWSSNNNTKDFYNLPGPSFKWYSPVLGNLVDLETCRDTLVSPD

WVKLGWTGRYRFLFGTERMYTLDPGAIGHILGRPDEWVKPTDFKTIMRRVTGEGLVTVEG

AQHKRQRRVLSPAFSTSALASMGPVLWTKAAELVQLFQALIADDTLEDLAARPPKPEDRV

PGARKIDVIRHMNSFSADVIGLCGFGHDFDSLSRKGPSDPLAEDFANLVDACAEDALMTE

AQNQFPLLDLIPTKPRRIVDRCKKSVDQISAKFVARKRAEIHDLGGGMELSEEEAGKDIL

SRLMAANARESGKAAMSDDEVRDQIATMLFAGSSTLSAVLYRLAENPRVQSKLRAELQEL

ADAPDMSVLDAAPYLDHVVRESLRLDSPVTCTVREAAKDTVLPLEVPVKGRDGRMMSEVA

VKKGTYVLLSFSGMHTAPELWGEDAMEFKPERFEEEGWPRMPIPGIVKNRAVCAPARVRV

CPSAEPAQDRADYDVHPQARGQGRDGIADAPACQECYGVRFSGFPWMEREEEGATLGVAL

SSGLLECLAT*

>CYP505AE1(940)Trichosporon asahii var. asahii CBS 2479

MGDGSSLPQAAAGPTKAAACPALAPTTSTPSATTSVTDHDNSKEKESKAKCPITGQEICP

NVKLTRGKDGYHNVPHPPFWPVLGSIPSIDVKQTIKSITELADTYGRFYMMSGAGHDFYI

CGSYEISKVLNDESRFQKTVHLPLEHLRPLVGDALFTAYPGEPNWDIAHRVLVPVFGPLS

LKKMQPMMVDVLAQMLMHWEHTAGTPFSAADQFTRLTLDTIALCGFRFRFNSFHSERLHP

FVDSMVACLLMSDERSRWPAPLLKLRWNHNRKYNANVKYMFDLCDKLIAERRKNPYPDAG

DMLNVMLHDKDPKTGKHLSDENIRMQIITFLIAGHETTSGMLSFTMFFLLKNLRVLAKAR

EEADRLVAEAGDNMLNINPSKATYIDWVLKESLRLQPTAPAYAVEPFKQDEPLPGDFCLR

KGDHCFVFLPLLHRDPAVWEKPEEFYPERWEHLSDLDPAAYRPFGNGARACIGRGFAIME

GIICLIMILHRFDLKLEDPNYELVIKETLTIKPDNMRIIATPRHSRSQSLLTELAQGSTL

AAGAPKAGEKKRRAMKTDNASGVPINILYGSNAGTCATLANEMAEEASARGLKPFVGELD

DTCGDGMLPSDGATVVIVPSYEGMPPDNARSFVTALETAGPMPNASFMVLGLGHPDWTTT

FHRIPKLVDKRLEELGAQRLMPITLADASQDVMGEFEAFSALVWEQLGVADVEGQTAAVL

EDVTVLPASANSNHADGFGFGSVVEQRTIFPATDSHPWTVHTVVKLPEGHEYQVGDYLCV

LPKNPPATVERALRVSGLHPDDVLKWGSLTVRAADLFTSYVELGHTAPRTLLKGMDYESA

RARHYTVLDLMEDTQLPLDAMLAALPRMKARHYSISSPPSPENTATITYTVHTAKGPSGE

VLGVCSNYLARLQPGERLQCATKSSPGFHLPPPQVPIAMFAAGSGIAPFMGFIAQRARTG

GRMTLWFGCRSAADLPYAAELLQYAQNGLDLRLCLSRSDDTSFEGLPVFHGYVQDRVRAE

KEDFLAMVDTGAQFFVCGSSNRLGSGLKKALIEVLGERSGNGEKDMEALSRGRYKTDVFL

*

>CYP505AF1(2977)Trichosporon asahii var. asahii CBS 2479

MTKTLDYSEGSTPIPGPAGYPLIGNLLDIDMSQCFVSLNALHKKYGPVYQISFLGQTIIM

VSSPDVVHVLSDEDKFHKHIDPTLLAVRGFAGDGLFTALHGEKSWDIAHRILVPAFGAIS

VRKMQEKMLDPLGQMLMWWECHAGEPFEAADQFTRLTFMFYAATSRQWDTDIALLNKVCD

EIISERRNDPMPDEHDVLDRMLFGKDAKTGEGLSDENIRYQMITFLIAGHETTSGLLAFA

TYYMLKTPGVIAKMRSEVEQALADVGGAFAELAVGKLRYVDAVLRETLRLHPSAPFFIRT

PNSEQGARLPGGYHVKHGQAVAISLHALHRDPEVFEAPEEFRPERWLDGTTYPSDAYKPF

GTGGRACIGRMFAMQEAALAMALIVDRFDLSLADENYELQIQESLTIKPVNFNIKVAPRK

KTGLLRELMTRSTVAKEEEAWKPVSAKGKMHVLFGSNNGACEALAGDLAADYRQSGFSVT

LDSLDDAAPDGKLPTDGTIVLILPSYDGQPAENARDFVASIANMEPGSLSGVEYTVFGVG

HRDWAATLHRIPKLVDSRLSELGAERLLPISLGDAAVDLLSDFEDFSARLRDHLEIDVTD

ISAWSIPDSANCAPVTGFTLGRVMKQTKLTDGVMSIAIQADQPWQVGDYLAILPKNPASE

VERALRVLGLKGDDHIRSPMAPGPLRAWDVLESYLELGTIIPKRYLTILPKWALSSASRL

AAMADDYATVRLQRLSLIDVLERFPDVRPPLGHVLYALPRIRARQYSIASTPPGLELTYS

VHERGVASRYLASLSEGDAVLCATRRSTFHPPPAQNTLVMFAAGTGIAPFRGFIAERAER

ARLGEKTGGTVLYYGARDAAHVLHDVELRHWVRNGVLELRPTLSRAERPKYKLVEGCRYV

QDRMWAERTDISAWLEGGAAFYTCGGTEFASGVRKCFTRIIAEKEGAEKAEDEMAKLGER

FKQDVFTS*

>CYP505AD3(2393)Trichosporon asahii var. asahii CBS 2479

MADVSAMVSPPSAQDLQALMSLDRKKDDEEALPPNHRGIVPWPRPPYYPIIGNLLDIDST

QIQDSIRVLVNKMGPAFEMNVLGNQTFVVASQEMCDFICNEAKFEKYISKALQDVRNYAG

DGLFTAFSWENNWDIAHRILVPAFGPIPIRKMQAGMLDIASQMLIYWECHAGQPFEAANH

FTRLTFDTIGWCSFKYRFNSFHSDTLHPFVEGMFELLRGSGHRAFRPAFLNKLFYKENAR

YWDTIHKLWATCDEVVAHRRANPDPDANDLLNNMINDRDPKTGEALSDENIRYQMVTFLI

AGHETTSSTLAFALYYMLKQPRIYQKAREESDAAIAAVGGNYLKLNPSQFPYIDAILKEA

LRLHSPVPAFSVKPKNPNGEILPGGYYVPHGKQISCVVENVHQDIASWGEDAADFRPERF

FEGFPKKPNAWKAFGNGARACVGRIFAIQEAILALALITARFDVELADPSYTLKVKQALA

LKPDNFNIKAYPRKGRHQNLLNEFLLGSAAITKEEAATANGTKTPPVSRRGKLYVLYGSN

SGTCEEFAQEVAAEGQASGFEINIGALDDVVPDAKLPTDAPVIICTTSYEGQPTDNAREF

IQSLRGLNPEDKPMAGVRYIIMGAGHRDWSETFHRIPLLVDKRLEELGGERLLELSLADA

GDDLLGDFEKFKGRLWDHFGTTEDAKDVKSTTADKPSIKVLPATANSASGTFADMSTGTV

VSQRVIGPAERDLPETRYITIRLPDGQSFQAGDYLNILPKNPNSTVDRVLKHFRLDGNAI

ISFDKAMPFIGANTPVRAADLFGSYVELSLPVAKRILPQLASMCSDAKDKAKIDALDADY

THSVTEKKLSLLELLESVPSCRAELDFFLGNLAKLKIRQYSISSSPLESKTEATLTYTVH

KPASKSGNGTYLGVCSNYLAGLSEGDELFCVVKSSGDFHLPEPSTPVVLFAAGSGIAPFR

GFIAERAAQKAAGEPVAGTHLYFGCRDETSFLHGAELGEWVKSGVLVLHPVLSRSQQASF

KKGGLKGTALPPAKKYIQDLVYEDREALRDLYKAGARFYTCGSGAKMAADLRKTAVRIIS

ELKGVSAEVAEEKMEEISREKYKTDVFL*

>CYP5139AT2(817)Trichosporon asahii var. asahii CBS 2479

MPALALDSAPSTYLAWASENWRTLVTYALTLWVSFKVARAVLRVFCPTLWMTSSLANVPG

PKPPSRLLGHQMESVRRSPGVTAEEWHDVYGPTIRLARPFGVAELSTVDPAALSFIYRAT

DERFVKPAGMRQAIAANVGDGVLAVEGHTHRRHRRVLNPAFGWPQIQDMLPRMWEKGYEL

RDKMLSSLSDPFYLRPEKACDDVPGARVMDMFGHLSNAALDIIGLVAIGEDLGALSDRQN

DLRTAYQDVLRVGFITDWLTVLRFAVPITRKIPTERGRVVKRSRETVEAFGHRVIAEKRR

LLQDMHSGRLEKKTDIGKDVLSLLIKANSAADLREDQRLDDTEVIAQITTMLFTGHETTS

TATGFCLRQLALNQGAQEKLRKEVLEAGADEPDFETLMALPYLHNVVRESLRFEGPVPNM

GRVATEDVTVPLSQPIIGRDGKLMDSVQLRKGDYVQAPYQAANRLTDVWGEDARQFRPER

WEQANFPHKKMPGVWGELATFGGGPHNCIGHRMAVLEMKVLLYIMLRNFKFEPVPSGPVI

KPKWMIIQRCVVEGEEARGPQLPLLVRPLEELQPTGTIANGRVLKAYKILGFISSGTYGR

VYKAVLLPPPKPSRGTVSRKSPSVSDDPLNNPELCMRPGDLPAKEGDVFAIKKFKPDKEG

DQQTYAGISQSGAREIMVRLLYNLPPSRADMQLNRELHHRNLVALREVILEDKAIYMVFE

YAEHDFLPANILVTSGGVVKIGDLGLARLWHKPLAQGGLFGGDKVVVTIWYRAPELILGS

KHYTAAVGRGSEARQQEDFAVPARSNGEDLRCPRPSEASNGYDLLTKLFEWDPTKRITAR

DALAHPWFQEDGGCAAESVFEGSTIPYPSRRVTHEDNGDAKMGSRLPGSSNFRSSSGAIP

PAKRTKLR*

>CYP505AD2(1487)Trichosporon asahii var. asahii CBS 2479

MSDPHNDKNDQNGGGEVAKTAEGTTPIPQPKETIFLGNLLDLDLNDKMGSLCRLAALYGE

IYQLKLKEYVVFLSSQRLVHYICNEQKYHKFISQPLKEVRAFAGDGLFTSYTGEHNWELA

HRILVPAFSPVAIRKMQPMMCDVITQMLMFWEHHAGQPFEAADQYTRLTFNRSSRPDLIQ

AIMLGSEREYREDTKELWRQCDEIVAERRRHPDPEAHDLLNNMILDKDPKTGEHLSDENI

RFQMVTFLIAGHETTSGLLSFATYYMLKHPNIFQKAREEADRVIAEAGGNLLKINPSHLT

YIEALLKESLRLQPTAPAWAVTPVSEEGDILPGGFKVNYGQSIAVLLPALGRDPDAWGPD

ADEFNPERWLDGREIKEDAWKPFGNGARACIGRIFAMQEAILAVALIVNRFDLTMADPEY

DLAIKQTLTIKPKDFNIIAHPRKNKNQSLLSELLAGGTASTKNDKVDTASHQQSSAAVAK

NGEKMYVYYGSNSGSCEGLAREVAAEAKAKGFDVTLGELDTACTSGKLPTDGPVVIVTAS

YEGQPTDNARIFVEALKNLPDGSEKGVKYCVMGAGHHDWAATFHRIPKFIDERIAQLGGE

RFMPLSLGDAGGDILEDFEDFKEKLWDHFKGSNPTPRGGVKVEAPEEDIKGGAPLSPEMK

MKILRPSILTEATPNCPQTNYVTIKLPEGLQYRAGDYLAVLPKNPEPTVERALKLFNLDP

NDNIVLNMPTSSLPSGVPIRIGDLLSSYVELGLPVSKRVLPKLEQFCADPLDKQRIKALE

DNYTQAVTEPRLALADLLEQIPGCKPPFGFFLSTLPKMKIRQYSISSTPLKNPEEVSLTF

TVHTTPSKTGKGSYLGVASNYIAFLEPGDCLNCTVKSSQEFHPPVDPAVPIVMFAAGSGI

APFRGFIEERALQKAAGREVGKTVLYFGVRTPNEIYHKDMLAEWVESGALDFRPVLSRSD

ATEIPGFPKDKVHTVPGCKYVQDRVAKESDDIVKLFDDGAQFYTCGSGARLGSGLKKVLL

DIIKEQPRCAGKDPQQILDKLAKDRYRTDVFLLTPGRGSTNHKLSIPDRRFLRAVARPTS

ADPSTGSCITSTWARLLLATSSSISIVDYSPTRLLTPGLRPSSRRVDTTSDCHRPHATTA

TMSFPVLRNSALLARSMPRAMPMGVRFRSTAPVRAAVSSTTPSMTTPPAFLGDANAAAGP

STGIKLEASPPPPRPRRKIASKKAPLTMTPEAIKRLEALQANPAEPKFLRIGVKTRGCAG

MAYHLDYVPQPGKFDEVVEQDGVRVLVDSKALFSIIGSRMDWRDTKLSAGFVFDNPNIVD

TCGCGESFLSERSIVY*

>CYP5887A1(2174)Trichosporon asahii var. asahii CBS 8904

MSVAYAVAGVFVAWLLYIFFPAVKSRQWFAHQLNGPPSPSFLTGRFFIDFWQKDHAPHMG

HVQRYGKIFKYPFALGIPMIFIADPYIVSDAYSHPDTYKKTKGMITMLDNLFGRGLLSAD

DDFHKQLRRIIAPAFTPSIIRSLFSEFLEEAYRLQERLQDKVKENNEVDILNELSRTTLD

IFGTTGLGQDQLGDPLNRLGQAYTSLIGALTADMGKLGMLQVIITQLQIFPSAMMRMIKT

SRAIIESKGKQMIAQAGQKRDKDLLSLLVKSNSNESASKQLSEQQIIEQITTILFAGHET

VSTSLSWTLYHLADNPSLQTRLHDELSELPEEPTFEQLDACPLLGKVVREILRVYPPVPS

TQRELAVDQDIPLSEPVALKDGRVITSLPLKKGEVLNLSVVCANRDKDVWGPDAEEFNPD

RFDRPNTPAKQIPSAWGNVLSFSHGSRNCIGFRFALAEMKALLFVIMRNWRVERVCPVEH

RNGITSRPYVDDKPSCPLRLHAV*

>CYP53A55(2921)Trichosporon asahii var. asahii CBS 8904

MTTSVHQDLSTRLDQLKSLPAGFWLAAALVAFSAYYLYPYFVTYAALRDIPAPFPAQFSD

WWLFRVVKRGNRSVTVDKLHQKLGKFVRLQPNHVSIADDSAIKAIYGHGNGFLKSSFYDA

FVSIRRGLFNTRDRAEHTRKRKVVSHTFSVKSVREFEPYMHDNLNLFVKQWDKLVDNAKK

EGSNGGKARFDCLDWFNYLAFDMIGDLAFGSPFGMLAAGADVAELRSTPDAPAEYAPAIE

ILNRRGEISATLGIVPKLIPYAKYIPDPFFSKGVEAVANLAKIAIAQVRGRLENPPADAR

TDLLARLQEGRDDKGKPIGREELTAEALTQLIAGSDTTSNSSCALLYWVTKTPGVLEKLQ

AELDEAIPPGTDVPTYDMIKGLPYLEAVFNETLRHQSTSGMGLPREIPSDSPGVTICGRY

FPPGTILSVPTFTIHHSKEIWGPDALEFKPERWFNVTQRQKDAFIPFSHGPRSCVGRNVA

EMEMKLIAATWMYRYKPTLLQDKLEVAEGFLRKPLGLEITIDRRH*

>CYP51F1(4578)Trichosporon asahii var. asahii CBS 8904

MDPYKFLFDCREKYGDVFTFILLGRRMTVALGPKGNNLSLGGKITHVSAEEAYTHLTTPV

FGKGVVYDCPNDMLMQQKKFSGSPVCCFCGSASGSPTPRSTPARSFRRNPTNAQIKHGLT

TEALSSYAELMRGETRQYFRDHVVTGKPFEVLEMMQQLIILTASRTLQGKEVRENLDIRF

AKLLEDLDKGFTPVNFLFPNLPLPSYKRRDKAQKEMSDFYMSIIEKRRSGEHDHENDMIA

ALQGSVYKNGVPLSDRDISHIMELQQMLLDEQVEKFGNPDGTFRDMTLEDTRGLPLMTAS

IRETLRMHAPIHSIYRKVTQDIAVPPSLAAPSKDGSYVIPKGHFIVAAPGVSQMDPQIWK

DAQTWRPTRWLEEDGVANAANEQYTSGERVDYGFGAVSKGTESPYQPFGAGRHRCVGEQF

AYLQLTVLVAEIIRNFKLKPVAAEFPKTNYQTMIVLPLDGRIVLEPRK*

>CYP504C3(6658)Trichosporon asahii var. asahii CBS 8904

MAGWSSEALSFDKLGLPAIVAAAVVFVLISEYYRYAKGKLHLPGPMPYPIFGNLPQLGKD

AALTMHKWSKKYGDVFKVTLGEREIMIINSAKATKELLSDQGGVYISRPMFHNYHSTLST

SAGFTIGTSPWDESCKRKRKAAATALNKTYVQEYLPIIDREALALVEDLYVNGKGGALEI

DPAAYCSRYALNTSLSVNYGCRLDSVGDDLLAEIIDIETGVSDIRSTTSSWSDYVPLLRK

LPTIRKTSGGYTTQEIKERRDKYMGLLLDDLKEKIAKGTDAPCITGNILKDPDAKLTPLE

LSSICLSMVSAGLDTLGNTFIWSLGFMAKHPEIWEKAYTELNKISNGNPPDSTEETCEYI

VALYREAARYFSVLKLSLPRATLGDSEYKGVHVPSGTTVFLNAWAVHHDEDRYGDVENFR

PERHLEQKDQEGALAHYSFGAGRRMCAGVHLANREMYVGFAKLIYFFKLEVGSEDYDINP

ETACKNTYGLSSTPFPYKVRFVPRDPEAIEQWIQDEKSRNDISIASAIKSKVNA*

>CYP55A30(4147)Trichosporon asahii var. asahii CBS 8904

MAFSPKFPFNRASGMEPPAEFATLRATNPVSQVQLYDGSLAWLVTKYEDVIKVATDERLS

KVRTRPGFPELSAGGKAAAQAQPTFVDMDAPDHMRQRGLVESFFTPEYVESLKPYIQRVI

DETLDKMAAKDKPVDFVSEFALIVPSYVIYTILGVPFKDLEFLTHQNAIRTNGSSTAREA

SAAAAGLIDYLGKLVDARMKEPKDDLVSTLCKAVEAGKLDRTSAVQVAFLLLVAGNATMV

NMIALGVATLAKYPSQLELLKADPSLAANMVQELCRYHTASAMALKRVALEDVVIGGKTI

KAGEGIIASNYSGNRDADAFPDPDTFDIRRKFDKDPLAFGWGPHRCIAEHLAKAELTAVF

ETLYKRLPNLKPAVPIEDIEYSPLDKDVGIVSLPVTW*

>CYP5139AV1(4313)Trichosporon asahii var. asahii CBS 8904

MTVAGAAAEALDRLPVVGPVLASWPKTTLTALIALAIFVALYAYLYVYRSLTLPFANVPG

PEPSSFFWGSLMEIIKAPPSAMHEQWYAEYGSTFKYRAIGGDWRLCSTDPGFLSWMLGHD

ELFPKPPHSQKFLWKLLGPGLVTVEGDHHARQRRVVSPAFSTKALKDQVPIFFDKAAELR

DKLLLFVEGKGDERPALPPSPGDEEKGGRKIDVMKYLGQMAVDIIGQAGFGYDLASLSGH

PKPLAEAFRVMMGAGLNPGPIQVLKTFIPFLSFIPTRADKILEESNVIAHREAAAIIAER

TREVRAAGDVDEGAFGKDLLSMLLKSNMSSDLRPDQKMTDEDVFAQVTTFMLAGNETSAT

ALTWILWRLAQHQDMQQRLRDECREVNEGDYSAVNSLPYLEAVVRESLRLDSPVPATLRV

TKEDVTVPLSKPIRGKDGTQMDSVRLKKGTTVMVPIHVVNWAKDNWGADSKTFNPDRWLK

EQSPWKYQNPGVFANLMTFNHGPHNCIGYRFSIAEIKVTLFTLIRALSFELLPSRPKFEM

KTGIVMRPMVVGQEKEGPQMPLLVRALEA*

>CYP61A1(6528)Trichosporon asahii var. asahii CBS 8904

MATHASETIPRASALPLNFDFVKAWGVENLSKARFQFDTKTTTATILTVIISLLLLEQVV

YRQKKKHLAGDKWTIPIIGKFADSLNPTLANYKKQWYSGALSCVSVFNIFIVIGSTNDMA

RKILNSPTYAEPCLVASAKKVLDPTNWVFLHGKVHADYRKALNVLFTQKALSIYLPIQDR

IYRRFFDEWCAENKTQEMMLKMRDLNMETSLSVFIGPYVNEQEKQEINKLYWDITVALEL

VNFPLAIPGTKVYNAIQARKVVMKYLMRASAQSKVRMADMDNEPECLLDEWIRAMILSKN

GNSEQAKLLSREYSDKEIGMVVLSFLFASQDAMSSAIVWTFQLTADHPEVLEKIREESMR

YASMHLAATISSAAVLMDWEHERTPDSDEVQVIAAIFPRDHCRLRFSKRAVA*

>CYP5139AX1(5744)Trichosporon asahii var. asahii CBS 8904

MTAIGAQGSALEAGRHLLTLIQAHPWLFSLAAFLAWFAARIYVTKYHGPFSKLPGPKRAH

WLIGNGIEVMNLTPKDAHQRWLDGTEGTVKIPRVLGQHYACVTDDLKASQYMWTHPDLFI

KPEGGRKILEDALGLGLVAAEGKLHRRQRRVLNPAFGWPQLVPMAPTIFEKAYELQRKMF

RTLEEPDGGKGVDHVPGKRKLDVLSFVSQATLDIISLVGFGYDCQALGDEPNELREAYNQ

TLATVFNITLMSIVQFALGRVAGWIPTERQRISHAVRAKSQEIGMAIVAEKKAQLLASNN

GVIEKNSQDLGKDCLSLLIKANMAPDLRDDQRLSDAEVADQCSTILFAGHETTGTGLMWT

LYHLACNQDVQEKLRRELLEVDAREPSWDELNALPYLDAVVHESLRLDSPVPMSSRVATA

NAVIPLSKPITLTNGEKINELHVPKGAEVSTPIAYINTSTKIWGPDARSFNPDRFARDGI

PAQQTPGVWGNLMTFLAGPRNCIGHRLTVIEMKIVLFVLIRSFRFEIPPSQPQLKKTWLV

IQRCVVVGEDKAGPQMPLMVSAVEN*

>CYP5139AW1(6773)Trichosporon asahii var. asahii CBS 8904

MLPLSELTSRLLGSLPPPLDALKDITVPTTATVWASVCSLPYVGPYIAAFPLASLFGTLL

ATLLAYAAYIYWSWFIYYPSQLPWRNLPGPPSKSLLWGNMLEFVYAPPSSAFYSWIQTYG

PTVRYMVACGNARILTTDAAFVQAMFTDCETWHKPWEAQLFLRRILGNGLTAIEGAEHIR

IRRVCMPAFSHRNVRLMGPTFFDKAEELRDIWLKYATRTEPAGWAEAAPFPPKPEDEAQG

GRKIDVMKGVMAMAIDIIGQAGFGYQLRALSTLQGEDNKLVDAFRNMIQGGMPNTRLQMF

LEMIPWFRGRTKAERTVEECAKLAESVLAERLRQSKAEVAAQMAAEKEGIATGPSGTEAK

DLLSLMLRSNMNPDIKVNQRLTDYDIQSQLRTFMLAGNETSASSTCWALLRMAKAPEIQS

RLRAECMTLGERPSIDALDALPFLDAVVREVLRTDAVVQASIRTAQRDTVIKLATPVRGR

DGNMITEVLMRRGDSAFIPMQVMNWDPEVWGPDSLEFNPDRFLRPGIPAKTVPGVYGNIM

TFITGRHGCIGWRFAVTEVKVVLFTLIRAFSFEELPSKPEVVGQLKVILRPAVKGEDEET

TYQLPLLVRALDTA*

>CYP5139AU2(8257)Trichosporon asahii var. asahii CBS 8904

MLYLVAAVLVPLALYLYLYPLREARLPYKNLPGPKPVSGFFGSLGELTARPLTSRYSQLK

DNYGTTSRFRALLGKWRIVSTDLTAITHVLRHTGTWHRHETFNVMIDRITGRGVLSVEDE

DHRRQRRILNSAFNGTAVNNMVPMFWEEGRILRDHIDQQLGGDEAKPLDVLQNYTGIALS

IIGRAGFNYDFQSHERDTNPLAVAFNNMINASLENKMAALLQNVFALPFNISPTSHFAQK

KSRSIVDSIGAQIVRNRKAEIERDHISLEKGDYEGKDVISLCLRANMLADQRDRLTDAEV

MGQIGALMLAGNETSANALSWATYHLCQNKDIQNRLREEVMAVPDEPSGEELDAIPYLDQ

FVKELLRFEPPLPQVVRQASEDTVIPLGQPVTGRDGKLMTEVHVSKGTDFVVPINLVNRL

PEIWGPDAETFNPDRWAQKGYPNTYIPGVWGNLLSFIGGPHHCLGFRFALLEIKAVLFVM

VRNYEFDFIPQGPQVMRIAPGIIQRPVVMGNEKAGIQMPVMVKKLSAEVREQAA*

>CYP5886A1(8319)Trichosporon asahii var. asahii CBS 8904

MFLFLACAAAFPLSLPLLYWWLQPPRNFAPGLPVVPLWIAFLPLLRSWLGFPLPGQDETY

ARYVEPLMAQHGAVVIYFGSQWNVLVACPQGMKQLFSLERTTFQKQGNHRKLPNAAISAL

TGGNIISETGALWRKYAQIIRPSLKDDTDPSKLDIAAERLIAQLEGHAGSVTGLVQRYSM

EAFLPSPPAIHRIHALVKRHIFHPLFLSVPALDRFPRLFPSRAIARNLIGNLEVELLKAL

PGAEARLKLGLPANEKDESGNVQSKLDGAFLSGFLSEREYIDNVKSETGPRPKLTAVLFI

AGHENAQQALLSLLWILSRDPELQARIRSDASPSPILHATVLEALRLYPPIPQLFNRRIA

HDTVLSLPGLDVSLKEGTYVGWSAYGLHRSIDAEWRPQRWGSSIEEMDANLRRWRSEGQF

ATFHGGLRTCPGQPFALASLHCVIRAILRVYEVTLSSEEGTQLTPGGLMAPRDLKLHFGK

RP*

>CYP5878C1(2917)Trichosporon asahii var. asahii CBS 8904

MIWLLTALAVVAALVYHFWSSNNNTKDFYNLPGPSFKWYSPVLGNLVDLETCRDTLVSPD

WVKLGWTGRYRFLFGTERMYTLDPGAIGHILGRPDEWVKPTDFKTIMRRVTGEGLVTVEG

AQHKRQRRVLSPAFSTSALASMGPVLWTKAAELVQLFQALIADDTLEDLAARPPKPEDRV

PGARKIDVIRHMNSFSADVIGLCGFGHDFDSLSRKGPSDPLAEDFANLVDACAEDALMTE

AQNQFPLLDLIPTKPRRIVDRCKKSVDQISAKFVARKRAEIHDLGGGMELSEEEAGKDIL

SRLMAANARESGKAAMSDDEVRDQIATMLFAGSSTAAVSLSAVLYRLAENPRVQSKLRAE

LQELADAPDMSVLDAAPYLDHVVRESLRLDSPVTCTVREAAKDTVLPLEVPVKGRDGRMM

SEVAVKKGTYVLLSFSGMHTAPELWGEDAMEFKPERFEEEGWPRMPIPGIVKNRAVCAPA

RVRVCPSAEPAQDRADYDVHPQARGQGRDGIADAPACQECYGVRFSGFPWMEREEEGATL

GVALSSGLLECLAT*

>CYP505AF1(5811)Trichosporon asahii var. asahii CBS 8904

MTKTLDYSEGSTPIPGPAGYPLIGNLLDIDMSQCFVSLNALHKKYGPVYQISFLGQTIIM

VSSPDVVHVLSDEDKFHKHIDPTLLAVRGFAGDGLFTALHGEKSWDIAHRILVPAFGAIS

VRKMQEKMLDPLGQMLMWWECHAGEPFEAADQFTRLTFMFYAATSRQWDTDIALLNKVCD

EIISERRNDPMPDEHDVLDRMLFGKDAKTGEGLSDENIRYQMITFLIAGHETTSGLLAFA

TYYMLKTPGVIAKMRSEVEQALADVGGAFAELAVGKLRYVDAVLRETLRLHPSAPFFIRT

PNSEQGARLPGGYHVKHGQAVAISLHALHRDPEVFEAPEEFRPERWLDGTTYPPDAYKPF

GTGGRACIGRMFAMQEAALAMALIVDRFDLSLADENYELQIQESLTIKPVNFNIKVAPRK

KTGLLRELMTRSTVAKEEEAWKPVSAKGKMHVLFGSNNGACEALAGDLAAEYRQSGFSVT

LDSLDDAAPDGKLPTDGTIVLILPSYDGQPAENARDFVASIANMEPGSLSGVEYTVFGVG

HRDWAATLHRIPKLVDSRLSELGAERLLPISLGDAAVDLLSDFEDFSARLRDHLEIDVTD

ISAWSIPDSANCAPVTGFTLGRVMKQTKLTDGVMSIAIQADQPWQVGDYLAILPKNPASE

VERALRVLGLKGDDHIRSPMAPGPLRAWDVLESYLELGTIIPKRYLTILPKWALSSASRL

AAMADDYATVRLQRLSLIDVLERFPDVRPPLGHVLYALPRIRARQYSIASTPPGLELTYS

VHERGVASRYLASLSEGDAVLCATRRSTFHPPPAQNTLVMFAAGTGIAPFRGFIAERAER

ARLGEKTGGTVLYYGARDAAHVLHDVELRHWVRNGVLELRPTLSRAERPKYKLVEGCRYV

QDRMWAERTDISAWLEGGAAFYTCGGTEFASGVRKCFTRIIAEKEGAEKAEDEMAKLGER

FKQDVFTS*

>CYP505AE1(8140)Trichosporon asahii var. asahii CBS 8904

MGDGSSLPQAAAGPTKAAACPALAPTTSTPSATTSVTDHDNSKEKESKAKCPITGQEICP

NVKLTRGKDGYHNVPHPPFWPVLGSIPSIDVKQTIKSITELADTYGRFYMMSGAGHDFYI

CGSYEISKVLNDESRFQKTVHLPLEHLRPLVGDALFTAYPGEPNWDIAHRVLVPVFGPLS

LKKMQPMMVDVLAQMLMHWEHTAGTPFSAADQFTRLTLDTIALCGFRFRFNSFHSEKLHP

FVDSMVACLLMSDERSRWPAPLLKLRWNHNRKYNANVKYMFDLCDKLIAERRKNPYPDAG

DMLNVMLHDKDPKTGKHLSDENIRMQIITFLIAGHETTSGMLSFTMFFLLKNLRVLAKAR

EEADRLVAEAGDNMLNINPSKATYIDWVLKESLRLQPTAPAYAVEPFKQDEPLPGDFCLR

KGDHCFVFLPLLHRDPAVWEKPEEFYPERWEHLSDLDPAAYRPFGNGARACIGRGFAIME

GIICLIMILHRFDLKLEDPNYELVIKETLTIKPDNMRIIATPRHSRSQSLLTELAQGSTL

AAGAPKAGEKKRRAMKTDNASGVPINILYGSNAGTCATLANEMAEEASARGLKPFVGELD

DTWGDGMLPSDGATVVIVPSYEGMPPDNARSFVTALETAGPMPNASFMVLGLGHPDWTTT

FHRIPKLVDKRLEELGAQRLMPITLADASQDVMGEFEAFSAAVWEQLGVADVEGQTAAVL

EDVTVLPASANSNHADGFGFGSVVEQRTIFPATDSHPWTVHTVVKLPEGHEYQVGDYLCV

LPKNPPATVERALRVSGLHPDDVLRWGSLTVRAADLFTSYVELGHTAPRTLLKGMDYEWA

RARHYTVLDLMEDTQLPLDAMLAALPRMKARHYSISSPPSPENTATITYTVHTAKGPSGE

VLGVCSNYLARLQPGERLQCATKSSPGFHLPPPQVPIAMFAAGSGIAPFMGFIAQRARTG

GRMTLWFGCRSAADLPYAAELLEYTKSGLDLRLCFSRSDGTSFEGLPVFHGYVQDRVRSE

KDDFLAMVDTGAQFFVCGSSNRLGSGLKKALIEVLGERSGNGEKDMEALSRGRYKTDVFL

*

>CYP505AD3(6313)Trichosporon asahii var. asahii CBS 8904

MADVSAMVSPPSAQDLQALMSLDRKKDDEEALPPNHRGIVPWPRPPYYPIIGNLLDIDST

QIQDSIRVLVNKMGPAFEMNVLGNQTFVVASQEMCDFICNEAKFEKYISKALQDVRNYAG

DGLFTAFSWENNWDIAHRILVPAFGPIPIRKMQAGMLDIASQMLIYWECHAGQPFEAANH

FTRLTFDTIGWCSFKYRFNSFHSDTLHPFVEGMFELLRGSGHRAFRPAFLNKLFYKENAR

YWDTIHKLWATCDEVVAHRRANPDPDANDLLNNMINDRDPKTGEALSDENIRYQMVTFLI

AGHETTSSTLAFALYYMLKQPRIYQKAREESDAAIAAVGGNYLKLNPSQFPYIDAILKEA

LRLHSPVPAFSVKPKNPNGEILPGGYYVPHGKQISCVVENVHQDIASWGEDAADFRPERF

FEGFPKKPNAWKAFGNGARACVGRIFAIQEAILALALITARFDVELADPSYTLKVKQALA

LKPDNFNIKAYPRKGRHQNLLNEFLLGSAAITKEEAATANGTKTPPVSRRGKLYVLYGSN

SGTCEEFAQEVAAEGQASGFEINIGALDDVVPDAKLPTDAPVIICTTSYEGQPTDNAREF

IQSLRGLNPEDKPMAGVRYIIMGAGHRDWSETFHRIPLLVDKRLEELGGERLLELSLADA

GDDLLGDFEKFKGRLWDHFGTTEDAKDVKSTTADKPSIKVLPATANSASGTFADMSTGTV

VSQRVIGPAERDLPETRYITIRLPDGQSFQAGDYLNILPKNPNSTVDRVLKHFRLDGNAI

ISFDKAMPFIGANTPVRAADLFGSYVELSLPVAKRILPQLASMCSDAKDKAKIDALDADY

THSVTEKKLSLLELLESVPSCRAELDFFLGNLAKLKIRQYSISSSPLESKTEATLTYTVH

KPASKSGNGTYLGVCSNYLAGLSEGDELFCVVKSSGDFHLPEPSTPVVLFAAGSGIAPFR

GFIAERAAQKAAGEPVAETHLYFGCRDETSFLHGAELGEWVKSGVLVLHPVLSRSQQASF

KKGGLKGTALPPAKKYIQDLVYEDREALRDLYKAGARFYTCGSGAKMAADLRKTAVRIIS

ELKGVSAAVAEEKMEEISREKYKTDVFL*

>CYP5139AT2(6473)Trichosporon asahii var. asahii CBS 8904

MPALALDSAPSTYLAWASENWRTLVTYALTLWVSFKVARAVLRVFCPTLWMTSSLANVPG

PKPPSRLLGHQMESVRRSPGVTAEEWHDVYGPTIRLARPFGVAELSTVDPAALSFIYRAT

DERFVKPAGMRQAIAANVGDGVLAVEGHTHRRHRRVLNPAFGWPQIQDMLPRMWEKGYEL

RDKMLSSLSDPFYLRPEKACDDVPGARVMDMFGHLSNAALDIIGLVAIGEDLGALSDRQN

DLRTAYQDVLRVGFITDWLTVLRFAVPITRKIPTERGRVVKRSRETVEAFGHRVIAEKRR

LLQDMHSGRLEKKTDIGKDVLSLLIKANSAADLREDQRLDDTEVIAQITTMLFTGHETTS

TATGFCLRQLALNQEAQEKLRKEVLEAGADEPDFETLMALPYLHNVVRESLRFEGPVPNM

GRVATEDVTVPLSQPIIGRDGKLMDSVQLRKGDYVQAPYQAANRLTDVWGEDARQFRPER

WEQANFPHKKMPGVWGELATFGGGPHNCIGHRMAVLEMKVLLYIMLRNFKFEPVPSGPVI

KPKWMIIQRCVVEGEEARGPQLPLLVRPLEELQPTGTIANGRVLKAYKILGFISSGTYGR

VYKAVLLPPPKPSRGTVSRKSPSVSDDPLNNPELCMRPGDLPAKEGDVFAIKKFKPDKEG

DQQTYAGISQSGAREIMVRLFYNLPPSRADMQLNRELHHRNLVALREVILEDKAIYMVFE

YAEHDFLPANILVTSGGVVKIGDLGLARLWHKPLAQGGLFGGDKVVVTIWYRAPELILGS

KHYTAAVGRGSEARQQEDFAVPARSNGEDLRCPRPSEASNGYDLLTKLFEWDPTKRITAR

DALAHPWFQEDGGCAAESVFEGSTIPYPSRRVTHEDNGDAKMGSRLPGSSNFRSSSGAIP

PAKRTKLR*

>CYP505AD2(7360)Trichosporon asahii var. asahii CBS 8904

MSDPHNDKNDQNGGGEVAKTAEGTTPIPQPKETIFLGNLLDLDLNDKMGSLCRLAALYGE

IYQLKLKEYVVFLSSQRLVHYICNEQKYHKFISQPLKEVRAFAGDGLFTSYTGEHNWELA

HRILVPAFSPVAIRKMQPMMCDVITQMLMFWEHHAGQPFEAADQYTRLTFNRSSRPDLIQ

AIMLGSEREYREDTKELWRQCDEIVAERRRHPDPEAHDLLNNMILDKDPKTGEHLSDENI

RFQMVTFLIAGHETTSGLLSFATYYMLKHPNIFQKAREEADRVIAEAGGNLLKINPSHLT

YIEALLKESLRLQPTAPAWAVTPVSEEGDILPGGFKVNYGQSIAVLLPALGRDPDAWGPD

ADEFNPERWLDGREIKEDAWKPFGNGARACIGRIFAMQEAILAVALIVNRFDLTMADPEY

DLAIKQTLTIKPKDFNIIAHPRKNKNQSLLSELLAGGTASTKNDKVDTASHQQSSAAVAK

NGEKMYVYYGSNSGSCEGLAREVAAEAKAKGFDVTLGELDTACTSGKLPTDGPVVIVTAS

YEGQPTDNARIFVEALKNLPDGSEKGVKYCVMGAGHHDWAATFHRIPKFIDERIAQLGGE

RFMPLSLGDAGGDILEDFEDFKEKLWDHFKGSNPTPRGGVKVEAPEEDIKGGAPLSPEMK

MKILPASLNPTGDHFSTGISSVGEIVSQSILTEATPNCPQTNYVTIKLPEGLQYRAGDYL

AVLPKNPEPTVERALKLFNLDPNDNIVLNMPTSSLPSGVPIRIGDLLSSYVELGLPVSKR

VLPKLEQFCADPLDKQRIKALEDNYTQAVTEPRLALADLLEQIPGCKPPFGFFLSTLPKM

KIRQYSISSTPLKNPEEVSLTFTVHTTPSKTGKGSYLGVASNYIAFLEPGDCLNCTVKSS

QEFHPPVDPAVPIVMFAAGSGIAPFRGFIEERALQKAAGREVGKTVLYFGVRTPNEIYHK

DMLAEWVESGALDFRPVLSRSDATEIPGFPKDKVHTVPGCKYVQDRVAKESDDIVKLFDD

GAQFYTCGSGARLGSGLKKVLLDIIKEQPRCAGKDPQQILDKLAKDRYRTDVFLLTPGRG

STNHKLSIPDRRFLRAVARPTSADPSTGSCITSTWARLLLATSSSISIVDYSPTRLLTPG

LRPSSRRVDTTSDCHRPHATTATMSFPVLRNSALLARSMPRAMPMGVRFRSTAPVRAAVS

STTPSMTTPPAFLGDANAANAAAGPSTGIKLEASPPPPRPRRKIASKKAPLTMTPEAIKR

LEALQANPAEPKFLRIGVKTRGCAGMAYHLDYVPQPGKFDEVVEQDGVRVLVDSKALFSI

IGSRMDWRDTKLSAGFVFDNPNIVDTCGCGESCSQQQDSRNSESAPSSRELRVSSQESFC

ASGNLGSLELLPVAVMAHAAWDSRNAARSADAQPASVSVKSGEADRDVIDKMWPGPAVTA

VSRVSPGHQLPIEQITESSLRMGVPTEIK*

>CYP5139AT1(263743)Trichosporon oleaginosus IBC0246 v1.0

MPSLPLVSGAIDWPFLLAVGGGAAALWLAYSLFHLLVPEARLSSSFKNLPGPPNPRNWTR

ILGHQYEALTRGPALTSEEWHDKYGPTIRLSRPFGRSELSTTDPAALTFIYRATDERFVK

SEGMRQAISANVGEGVLSVEGHTHRRHRRVLNPAFGWPQIVEMVPSMWLKAYQLRDKMVS

NITDAWASPPADPRDVVPGARVLNMFNELSNAALDIIGLVAIGHDLGSLSDEPNELRDGY

NDVLRVGFQTDIWTVLRFALPITRKIPLERTRIVRAGRDAVVRFGQRVIDEKRRLLLNID

QGRIEKGTDVGKDVLSLVIKANAAADLRDDERLNDTEVIAQIATMLFTGHETTGTATAFT

LRHLSLNQRFQDKLRAELLTIDVDEPDFQVLDKLPYLHAVAREGLRFETPVPIMGRIATA

DVAIPLSIPVRGRDGKMIDVVQLKKGDLINAPYQAANRLTDVWGADAREFNPERWFRPNM

PAKKMPGVWGEIATFGGGPHNCIGHRMAVAEMKVLLFVLIRNFIFSPAPSGPVIKPKWMI

IQRAIVEGEEHKGPSMPLLVRPVAE*

>CYP51F1(271374)Trichosporon oleaginosus IBC0246 v1.0

MAMDPSAVLDWAHALPLPVKIVGAVIGVPLLVIVLNVLRQLIVPTDPTAPPVVFHYIPWF

GSAAYYGMDPYKFMFECRDKYGDVFTFILLGRRMTVALGPKGNNLSLGGKVSHVSAEDAY

THLTTPVFGKGVVYDCPNDMLMQQKKFIKHGLTTEALQSYASLMPAECHGYFNNELKITK

ANPGPKTVDVLHVMAELIVLTASRTLQGKEVRENMSIRFAKLLEDLDKGFTPLNFMFPNL

PLPSYRRRDLAQKEMSDFYMSIMAKRRTGEHDHEPDMIQALQGSVYRNGTPLSDRDIAHM

MIALTMAGQHTSSATSSWFLLHLAHDQDIQKRLYEEQVEYFGNPDGTFRPMTYEDTKKLP

LMDSCIRETLRLHAPIHSIYRKVLQPIVVPQSLAAPSEDKAYVIPKGNFIVAAPGVSQMD

PKIWDDAPRWNPLRWLVDGGIAKTANEQYQAGEKVDYGFGAVSKGTESPYQPFGAGRHRC

IGEQFAYLQLTLVVGEVVRNYKLTAAQREFPKTNYQTMIVLPLDPKITFEARV*

>CYP504C2(274577)Trichosporon oleaginosus IBC0246 v1.0

MSSKLSAFDLNNVQLGFNSQTLAIVGVALLAISVVQYVRYARGQIHLPGPTPLPIFGNLL

QLDDDAAQTYYKWSKKYGAVYKVTLGEREVVVVNTAEAAKYLFGDLGNIYISRPLFHNFH

NVVSSSAGFTIGSSPWDDSCKRKRRAAATALNKTAVQSYVPIIDRETLALIEDIYRDCGK

GTKEINPYSYLQRLALNVSLVVNYGARLDDISDAMFHEIIEVETTVANFRSLSNDMADYV

PLLRYLPTSKSTVDVAEDARKRRDKYMKKMLDDLKARVANGTDIPCIMGNILKDPEAQLT

ELELSSICLSMVSAGLDTLANTFIWSVGYLAKHPEVQEKAYKAINDVYHGAIPDSTEEVV

EYITALHKECSRYFSVLKLALPRATIGDSEWRGVHIPDGTTVFLNAWAIHHDAERYGDFE

TFRPERFLDKSEANQQAHYSFGAGRRMCAGVHLANRELYVAFCKLIYFFKLELGSEDFDI

NPATACSNPRGLSSQPHPFKIRFVPRDADTIEEWIADEKNRAELKLAASMTNKQ*

>CYP5700B1(281659)Trichosporon oleaginosus IBC0246 v1.0

MSASALVDTLLSHSTLTKVVLAVLTLLLAVVIELARRRATYAHLPPGPLPHLLTGNDYPA

TYPWRAFYELSKTYGPVITLWTGTQPSVILNDMASATWFLDKHSRDTSDRPPHWAYISGG

KRVILQPHGERWRRMRRALYSILQPAQATQLRGYQERVARGVVLDILGGGEFQDHIRTYA

ATISVHMAYGRMERARYSDPDIAKIVENSARFGNFLRPGGSKLDAFPWLAYVPGYMRQMN

RWNAEELVLFRSALDDVKARSVTGPERCFATYLLERKKELELSYDEVAYLCGSVFGAGSD

ATSAAIQIIVMAAATHPEWQARVQAELDQATAGQPPGFDDLAVDAVPHLHAFVAESYRWR

PVSAGGFIHRTTAPVMYGDYVVPAGVLISGNHWAIHRDTSYFGADVETFNPSRWIKDGKF

NEAMKHVQFGFGRRVCPGHHVAHNSVLINAALLLWAFNMTEQTNSKGKLIPIDTLAFTNT

ANSHPLKFNVSFHERVANLAELMSVDNI*

>CYP53A54(285638)Trichosporon oleaginosus IBC0246 v1.0

MDSKHVNVKLPAVIQDLPAWAPFAAVAGCVLWYYLYPYFVTYGALRDIPAPFPAQFTNWW

LFLVVRRGDRYATADREHKRLGKMIRLAPNHVSIADDSAIKAIYGHGNGFLKSDFYDAFV

SIRRGLFNTRDRAEHTRKRKVVSHTFSPKSVREFEPYMQENLHLFVKQWDNLIAKEKMNP

KSNGQPKVDCLDWFNFLAFDMIGDLAFGAPFGMLEAGADIAELRSTPDAPATFAPAIEIL

NRRGEISATLGVFPQIRPYAKYLPDPFFSKGIEAVQNLAKIAIARVKQRLDNPPADARND

LLSRLQEGRDHKGQPIDREELTAEALTQLIAGSDTTSNSSCALLYYATKTPGVLAKLQRE

LDEAIPEGTDVPTFDMIKSLPYLEAVFNETLRYHSTSGIGLPREIPPGSPGVTICGHYFP

AGTVLSVPTYTIHHSKEIWGPDADEFNPERWFNLTARQKEAFIPFSHGPRSCVGRNVAEM

EMKLIAATWGNRYHPTLLQDVMETREGFLRKPLALNIEFKQRRRAVV*

>CYP5878A1(285737)Trichosporon oleaginosus IBC0246 v1.0

MADLAEYRDTVMHPDWVAMGWTGRCQHIMGQETIWTYDPVAMGSILQSADVWERAKNTER

LLRRITGSGMLTAKGAAHRRQRRIVNPAFSTTAIKAMVPAMFEKAELCATLLGRCVDDDS

LESFASRYPPKPEDRVPGARKVDMLALCSKLTQDVIGAAGFNTDLESLRPADNPLDNSIQ

YMLNTIFDDTIISMGQNLYWLLDKIPTRTRRAMKTCRTEMERVSERLMTERAAKQEGSVV

DADEDKVPDMLDLLLKANSAEREDQRLSDEEVRSQLSTLLFAGSTTTAGTICSLLRFLAL

HPDIQARLREEIEATEERPSFETLNALPLLDATVRETLRLEPAATCTVRTNTKDTVIPLS

VPVRGRDGTMIEKALPVAKGSYVLLSISSLQRHPDVWGPDAEEFNPDRYKDPSIPKMNIP

GMWGGLASFISGPHHCIGWRLALAEIKIVTFTLLRHFAFEELPSKPDIFIVMNVASRPEV

KGESGGQMPLLVRRIEA*

>CYP5139AR1(286793)Trichosporon oleaginosus IBC0246 v1.0

MTESLIASGAARAVAAITSLSLLQVTGYLVLGVLVGLVGTWAYLYPYAAYFSSSRNLPGP

PSEHPIAGNLLYVLRRQHPRPVYQAWIDKYGPTGRIRGLLGVERIYTSDPAVISHIFQHA

DMWPKSKSTNIMLRRMLGNGLITAESSDHRRQRRVLNPAFSPRAVHEMAPIFFEKARAVR

EKFAQLLEESATTRDRVVLVDTDAMQATDNKGGKIDVGLLMEQATLDTIGAAGFDYDFAT

LFSRRSELLDAFHSAVKALQASALISALQNRFVLFNALPTPGLKLGRASRATMNRVGDRI

VAEKTRAITNMGIEKDTDLATADLLSRLIRANMAVDLEERARLSHAEVQAQICTFIFAGS

ETTGVALTWALYRLATDKAMQDRLRAELVTISENPDLDEIHSLPFLEKFTREILRMDSPV

PAVGREPAAKTTLPLSRPVRGRDGKMMDSVVVDKGTEVVILVGNVNRDPAIWGPDSNEFN

PDRYDRPPLASVPGVYGNLLTFIGGPRNCIGWRLALAEFKIILFTLLRAYELAEVPGTTL

HSETTLGIMRTRSNEEGVVVPLLVRPVGE*

>CYP61A1(288204)Trichosporon oleaginosus IBC0246 v1.0

MSSQAIPRATSLADKTWGLDSLTKARFSFDSKTTAATILTVVISLLILEQIVYRTKKAHL

PGDKWTIPVIGKFADSLNPTLENYKRQWYSGPLSCVSVFNIFIVIGSSNKMARKILNSPN

HAEPCLVASAKKVLDPENWVFLHGKVHADYRKALNVLFTQKALAIYLPIQERIYRRFFKE

WVSYKGARPMMMRMRDLNMETSLSVFIGPYLTDADKDLINKKYWDITTALELVNFPFAIP

GTKVYNAIQARKVVMKYLMNASASSKVRMADMQNEPECLLDEWTRAMILSKNGNSEEAKL

LSREYSDKEIAMVVLSFLFASQDAMSSAIVYAFQLTADHPEILAKIREEQMRVRGGDLDA

PLTLDLVEDMVYTRAVVKEVLRLRPPVIMVPYLTTREFPITEDYTVPKGAMIIPAFWNSL

HDETCFPEPDKFMPERWLPNEDGSAPLAESKPQNYLVWGSGPHKCIGPQYAGMHLAATLG

TASVLLDWEHVRTKDSDEVKVIAAIFPKDEFIASFTERAAM*

>CYP5139AP1(297921)Trichosporon oleaginosus IBC0246 v1.0

MEGVVELARAHPILTLLSLATLFPAFLILWAYPYREYTLPYRNLPGPKPSSVFWGNYSEI

LKEPTNAPQTRWIADFGNAFRYRTLFGKPRICLADPVAIAHIAQHTYDYHKAGPTVYALE

LVLGRGLLTVEGDDHRRQRKIMSPAFGLPAIKSMHPIFMEKAWELQRKLSAILDTEEETY

CSTPVKPEDRVLGTKKVDMIPYWGQMTLDIIGLAGFDYDFGALAGRSTDLADSFRDLLRA

GNSPGILAILAAVIPPLRALPNKLTNAVGAAKAQTDRVAREIVQQKKVLVAAELATGEKD

ETLGRDLLSRVVRANMDPALRPEQRLTDEEVIAQVVTFIIAGHETTATALTWMVFRLAEN

WHVQKRLRDELRAFPHDNPSFDELNALPYLDKVVHEGLRLDPPVAGGVRVAAKHYIIPLG

QPIRGRDGKMISSIDVPVGTDIFIRVYGNLLTFFNGARNCIGYRLTLAEMKAALFCIFRH

LEVLPLPSNPEIRLKAQIVMRPLVVGEEEAGYQMPVLVRAIPDDD*

>CYP5139AU1(301118)Trichosporon oleaginosus IBC0246 v1.0

MSTVLAQPLPLTLTPQVVGYVLLSLVVVAPLALWFYLYPYTAATLPFKNLRGPEPTSGFF

GSLITVSTKPIGQRYASLTEQYGASTVRFRGLVGRWRIMSTDTTAIAHVLRHTGQWHRNE

GFNGLIERMTGANVLCVEDEPHRRQRRILNSAFNGTSVSAMMPMFWEEAYDLKKNVEELL

VEGQEKGTQVDMLQVYVRTAMDIIGRAGFNYYFEQHKVNGENPLSKAFNDMINGMLENRK

LVLVQNIFPQALDLPTANHRRVAKSRAVLDGIARDIVQSRRAEIARDHISLDKGDYEGKD

VISLCLRANMGASERDRMTEAEIEGQIGALMLAGNETSATALSWATLHLFENPDIQAKLR

AEVMAVPDDEPSVEVLNGLPYLDGFVRELLRLDPPLPQVVRTAAVDCVVPLGTPVQGRDG

SLISEIRVSKGTDFVVPISEMNRDPRIWGADADKFNPDRYDGTVPRVSLPGVWGGLLSFI

GGPHHCLGFRFALLEIKAVLFVVLRNFAFEPVPGKQFIRFSAGIIQRSIVIGEESKGMQM

PVIVRALESE*

>CYP5882A1(324737)Trichosporon oleaginosus IBC0246 v1.0

MEALKTFTAQLTPATVSAQSNILAGATALGVLAHLYWQRFEPDPISYGQFALALGGGLVY

FFYHFLGSLPTAILYTAEVFTVFNTVVLLSMVIYRLSPWHPLAKYPGPAAAKVSKIYWWY

QAKIGQTGYTQAKLHDQYGDFVRIGPNWISVRSADAIPIIYGGSKLGAGHGRAPWMKNDW

YEGAIRGATRISVHQEVDVDKHHIRRKLWDHGFTGKALADYKPEILDFCDKLVRNVNMVV

ARGNATGKPQAIDMGLQSSYFSFDVMGVLGFGEPFGMLDAGVKHLFVDTLEKAMRTLIPF

HEISWIRILLRKLLPLPERLKVFEKQNEERFERREKLGSARRDLFTYLLGEEKDGAETFK

LTRLELIADAGTIVVAGSDTTSSLLTWFWYYSTKHPETYKTLRDELAGLNADQLIDTTLL

SKLPYLNAALNETMRMQPAVPGGLRRLVPMGGVEIEGHYLPEGTVLSVPGYAIQHDPRFW

GKDCNEYRPERWLDTSGKYNKNAYLPFSYGSRGCPGKGLAMVEARLAVAQLATRFEFAFP

PGFSQREFEEGVRDHFTIQNMPLPLVPIPSPVAKAA*

>CYP5139AQ1(240056)Trichosporon oleaginosus IBC0246 v1.0

MLAGLADAPRWALYSVAATILFTAFMIYRYPYRERVLHYRNLPGPPSSHFLFGDFPKVQS

HPTGRVMQAWFDEYGPTVRAKLLLGDNLIVTSDTTAMAFIMQHADHFIKPPAQTRMITRL

LGHGLLNVNFDTHRRQRRVLNPAFHPDAIRDMVPHMFTKAYELRDKLMEACADPEYYDPE

TAAPRPEDVVPGARKVNMNKYLINTTFDVIGLAGFDYEFGCLRTGNHIVTTFRNAMSELQ

RLTLFGVLQQMIPALDVIPSKRNKLADQARIGTSGVGRECIARKRREMEAMHQDDLNKGT

FVGKDLLSLMLKANMAKDLLPRDRLDDTEMAYQVSTFILAGSETTSNSLTWTLYRLAMNP

DVQQRLREELDTLASDEPSLEQLNTLPYLENVIHESLRLDPPVPESMRMVTQDVMLPLHT

PVKGRDGTMIDSVPMKKGDMIITGYMQVNHNKEIWGADADRFNPDRFDRPGIPARKAPGT

WGNITSFNGGNRNCIGYRFALAEIKAILFVLLRHFQFEMLPSSPTITAKNFIIMLPWVVG

EENVGSQMPLMVRPIVE*

>CYP5139AN1(241190)Trichosporon oleaginosus IBC0246 v1.0

MVFRDHFGAWRNFPGPRPTHWFWGNIHEVMQTSPQQCHQRWLDKYGRVVRVHDVGGRAKL

VCSDPVATQYLLLHPDAFIKPAGPNRILTDALDKGLVTVEGRAHRRQRRVLNPAFGWVQI

QGLAPMIMDKVGEMRDKFARLIEEEMAGTGRVGRRMDVLHYMSETALDIIGEAGFGYDFN

AIGDANSPLRNAYTSAVQHAFEFDATVMLTVMDARFGAIPTSRVRNMHAARSAAQVIGRE

IVAEKKKQVINYNKGLDKESLGKDVLSLMIRANMDPALRPDQRLTDDEVVAQIATVLFAG

HETTSTALMWCLYHLSLHQDMQQRLRDELLEVPDERPDFDTLMNLPYLDKVVHESLRLES

PVPNISRVATQDGVVPLSAPVTGRDGRPIEAIRVRKGEVIEVPLTYMNTLAEVWGPDARE

FNPDRRDTGVPTQQLPGVWGGIMSFIGGPRNCIGHRLTILEMKAVLFALLRSFVFEQLPD

PPKIKRTYMVIQRAVVVGEEEYGPQMPLLVRPYVET*

>CYP5139AJ1(285220)Trichosporon oleaginosus IBC0246 v1.0

MSTMSSLTDIPFVAKLADIPVVSTLAAHPYITAVLLTLVLGYIYCFPYSDYRYGIHNVPG

PPNPHLFWGNLQEVIKAPPNAMHEQWFNTYGNVIRYKFLMGAQRLTSVDMGFIGYVLQHN

DEFIKPVRAQEMLERLLGNGVLIAEHAAHRRQRRVLNPAFSLAAIRDMTPIFYDKSYELQ

RKLAELIEEPSELDQAAPTPAKPEDVVKGARKIDVMRYMGAATLDVIGLAGFDYDFNALR

DNKNELAEAFRTMFSAGTSPSAMNILQNFIPILQFVPTKAREATRRSRAVTVRIGKELMD

NKKAAVRALFAGEVEKGSDIGKDLLSLVIKANMASDLRPDQQLTDDEVLAQITTFMLAGN

ETSSTALTWLLYRLALHKDVQDRLRAECQAVPTDRPSMDEINALPFLDQCVHESLRFDNP

VASTIREAVSDQVIPLSEPVMGRDGKMMSEIHVKKGTTIFLPIMNVNRSKEIWGEDADKF

NPDRFSVGGIPRKHVPGVFGNLLTFLGGARNCIGYRFALIEIKMILFVLMRSFEFDELPS

KPEYEAKSS*

>CYP5878B1(283338)Trichosporon oleaginosus IBC0246 v1.0

MANTTLRVPLLPFSGGWASIPLASWWAPLALTLALWLISRLLKPPHSPRNTPEVRALPGP

ARPRFGWLLGNMAQLADHRETLIHPDWFQWGWTGVYESLFAQKRIYTFDPAAVGYIFAHP

DEFERPRDLRTILGHIMGADSLVVNEGEKHRRMRRALNPAFSAGAIKEMMPAMLEKAGEM

VALFERLIDEEALESYAARAPAPEDRVPGARKIDVFKFAGNLTTDIIGIAGFEHDFNSLS

PEDDSPALLANRFNRLVDAANGMVLLTEAQNIMPVLDKIPTQSRRVIGEVRGQMERLSDT

LFARRKEVGYDPGAKDLLSLLVKSASATKSDAAMSHDEVRAQMASLLFAGSTTTAATISF

ILFELAKLSEWQEKLRAEVLAGHARPSFEALNAFPLLDAVIRETLRLHPPASCTCRATTK

DMVLPLAKPIPLRDGRTIDALPVSKGSYFFISMVCLNRLKEIWGDDAEEFNPARHADPAL

PRMQVPGVWGNVSSFISGPHACIGYRLAIIEIKVVLHALLRAFEFELLPSRPHIQHFSSL

ATQPQVKGEPGGQMPLLVRRHRV*

>CYP505AD1(253354)Trichosporon oleaginosus IBC0246 v1.0

MPSKEVTRPAGKSGEKGRPIPQPAPKLIVGNIFDIDPLKSMQSMMRLARIHGEIYQLKLF

EMIIFVSSQELVHYISDETKFEKCVSKPLQEVRNFAGDGLFTAFNSEHNWTLAHRILIPA

FGPLAIKKMQPMMLDVITQMLMFWEHHAGEPLEAADQFTRLTFDTIGYCAFKYRFNSFHT

EKVHPFIDIMVKLLAESQARSRRPGLMQSVMWSSEAEFRANVKELHRLCDEIVAARRRNP

DPNAHDLLNNMILDSDPKTGEHLSDENIRYQMVTFLIAGHETTSGMLSFATYYMLKNPST

FAKAREEADAAIKKAGGSLLKVNPADLKYIDAICKEALRLQPTAPAWAVVPKSEKGETLP

GGYYIAPGQNVVALLLMLHRDPKAWGEDAEAFRPQRWLDGRVIPPDAWKPFGNGQRGCIG

RGFAMQEAVLAIALIVARFDLEMADPTYDLEIKETLTLKPENFKIIARPRFGRNQSILSE

LLASANGSNNYTYTTEAKGDVATSSGGHGEKIYVLYGSNSGSCEGLSKELVSEGDARGFN

MRIGELDSVAGGGVLPTDAPVIIVTASYEGQPTDNARMFVAALAANKNTDSLKGVRYMVM

GAGHHDWAQTFHKIPKFIDTRLAELGAERLVPLSTGNAAEDIVGDFEAFKARVWKHFGGG

PASTDAAAMPADESAQPFLLLPRTANPAAATVGCGIETVGTVIEQDVLTRVSENSLQTNQ

ITIQLPKGQQYRAGDYLAVFPKNPKPTVDRALEHFGLHTEDTVVFNRPTSFFPLKLPMRV

GDMLSSFVELGQPVSKTVLPQLLPHCDEQATKRLEALEANYVQEVVGPRLSLLDLVLTTP

GCKPPMDVFLANLPKMKIRQYSISSTPLEEPDKVTLTFTVHTAPSAVGDRVLGVASNYLA

FLNRGEDLLCTVKGSAGFHLPADPEVPVVMFAAGSGIAPFLGFIAERAQMAAGGRKVGRT

VLYYGTRTSVEMVRKDKFAQWVKDGVLDFRPVLSRSNAKEVAGVPGVALLPEAKYVQDRV

WVDKDDMADLFAAGAQYYTCGSGQRLGHDLKQTLVKIIAEKKPEGDNETPEAIMERFARE

RYRTDVFL*

>CYP51F1(38441)Tremella mesenterica Fries v1.0

MSLSIASVPWAPQWLLSLPSPLLVLLGIIGFPTLIIFLNVFRQLVLPRDKNLPPVVFHYI

PWFGSAAYYGEDPYKFYFECREKYGDVFTFVLLGRNITVALGPKGNNLSLGGKTTQVSAE

EAYKHVTTPVFGKGVVYDCPNEMLMQQKKFIKNGLSTEALASYPPLINAECEHYFEKELG

FSPTSPGPKTIEVFKIMSQLIILSASRTLQGKEVRESLDSKFAEYFHDLDGGFTPLNFMF

PNLPLPSYRKRDKAQKSMSDFYLNIMQKRREGATDVNGSFDMLAALQGCVYRNGVPLTDR

DIAHMMIAILMAGQHTSSATSSWALLHLAHRPDVADALYEEQKTLFGNPDGSFRPVEYGD

EKRMTLMNSIIRETLRIHAPIHSIYRKVISPMPVPASLSAPSESATYIIPKNYYIVAAPG

VSAMDPRIWKDANVWEPFRWSDEKGTAAMALNEYTTGGEKMDYGFGQVSKGTESPYQPFG

AGRHRCVGEAFAYVQLSVIIAYIIRNYSLRLETPDGLFPKTNYRTMIVLPLNGLMSLTKR

KQEV*

>CYP61A1(40696)Tremella mesenterica Fries v1.0

MNVVSHTIVRPTSVDLAGLTTWGLEGLTKTNWKFDTKTTAATILTAIISLLILEQLVYRS

KKAQLPGARWTIPIIGKFADSLNPTMANYKAQWNSGPLSAVSVFNIFIVIGSSNEMARKI

LNSPTYAGPCLVQSAKKVLLPENWVFLHGKVHADYRKALNVLFTKTALSIYLPIQERIYR

SHFAQWVSDTSPAKPYMMIMRDLNMDTSLSVFLGPYLTEAQKIELNQKYWDITVALELVN

FPFAWPGTKVYNAIQARKLVMRDLEAASAESKRRMADPNSEPQCLLDAWIKAMIDSRHGD

DNVQGEEGEQQRLLSREYSDHEIAMVVLSFLFASQDAMSSALVYAFQLTADHPEVLEKIR

EEQYRVRGNDVDASLTLELVDDMVYTRAVIKEVLRLMPPVIMVPYTTTRPFAITPDYTVP

KGAMLIPAFWNSLHDETVYPEPDGFKPERWLPNPDGSAPLADSKPQNYLVWGSGPHKCIG

GQYASMHLAATLGTASVLMDWEHERTKDSDEIQVIAAIFPKDGMKLKFHPRVAPS*

>CYP5139B2(36021)Tremella mesenterica Fries v1.0

MKSSDVFRTLSAALSQRELSLADITLYALGTSCVALLGAYLYLWPYRAYTLPFKNLQGPE

PDSWFFGSMRRIIKEPPLAPHQAWLETYGPTYRYRLFFGSWRFFTADPLALSYILNHSDS

FPKTGMARREMETILGRGVLIAEGEDHKRQRKVLNPSFSLTAVRGMLPIFYDSAYELRDK

LQGLIEDPNAMASPTPAKEEDKVEGGKKVDVMKYLGQATIDVIGKAGFDYDFQALKAPTN

ELADAYRSLFSTSMTITPMAILQALFPVFKKLPTKRMRVASEAIRTTKRIGMRLLEEKKQ

AVRKAHSEGLEKGTDIGRDLLSILVKANMASDLKPEQRMSDSEVLAQITTFMLAGNETSA

TALSWICYVLSMHQDVQDRLREEVTSVSDDRPSFETLTQLPYMDKVIREVLRLYPPAPST

VRQAEEATTLPLSKPVKGRDGKMIDHVNLPKGCVIFVPIMCINISTDVWGSDASTFNPDR

YDNLPSSSNQVPGVWGNLLTFLGGTRNCIGYRFALAEIKAILFVLIRSMKFEELPSKPQF

EKKAAIVMRSRVIGEEDAGYQLPLLVKLLSQ*

>CYP5215B1(69516)Tremella mesenterica Fries v1.0

MTIPIRIPSLGEVVLLVGTYVGLRFLHYVVWRPLTSPFRQLRAPPGGNGWMGHYPLLVDY

YFIQGLDEWVTKLGKTFQINGLLWVNPRLVTIDPRAISHIFGNTSLYVKPERLRLLLGRY

MANGLIPAEGERHRLQRKVVQRLFSRQGLKDMTEGIQKKVEQLRDTIADLCSDLTLSPPY

TRDDLMMNLPETYRIIDIYDLTLRCFYDVIGQTTLGYDFDCVAEWNGQGGKIYEKYEKMM

QPYNGMYSWDQHLRIILPLLNKIWPSENLRRVNAGMGPLKALAKKRVAERKREMAEGNDS

KDQKDLISVMLRANLANPPDQQLTEDEIIGQLATFQFAGSDTSAGTVAFCLWQLALQPEF

QSRLRAECLAYGDKLPFENIDDLPYLNAVMMEALRCNPSIPGTLRQATQNDIIPLASPII

NTDGKPITEVRIRKGQFVQIPIELFNMTHTSWGSSPQKFDPSHWLDYDGRSINLPSVTSS

PTDTSISQDRDNENEHVVENIGYHGLLTFIDGPRKCVGYKLGMIEIKLVLFNLIKEFIFM

PVPNRKIYKWNL*

>CYP5216B1(72868)Tremella mesenterica Fries v1.0

MTSWILLLGIVIIPLIFLRRWLSLRHDLQSAARLGARPIPVVKGKWPLNFDVVLSWTRWS

QDEVGRMLFELEKKYGTTFNTRTLGEDQIISSDPIVLRHVFMTGFDNFVKGPKFHTRTRG

YLGDGIFNTDGSTWHSHRTTLRPFFRAERIRPELFEPIIDKFLLSLPTAASLTPDHPVPH

KDIITASPPFTMPNEHSASLADSKTTFNGNTTKAFDMQALLGRLTLQIALLWLTGEDISS

NLSDNDNSSVLPRAPEWRQALSELPTALKQAQEIVAFRLKIGWPWRLLELGRSPLRKPMK

TVHAFFRPLIDEAYLRQSGTSLLDALVHQEGMSKEKVQDQLINVLLAARDTTTVLLTCCT

YLSLLQPHLQDDSEYKNEHDTGYVEEVKMTRTALKELTKCRAFIDETLRLFPPVPINVRR

AVNDCVIPTSQGPMFMKGGTTIILCPLLMQRSSDYWEDPLDFKLDRWRKETDGNVNREKR

QKEEQSYMPWNVGPRTCLGQNMAIAVSMTFLVKWIRYLHRMKEEGVELVLAESPTALRTG

KGGEDDGDGKIPGWWFIHGAGRLRDGRDKVWMTSNMTLNVKVSSSASPPYFL*

>CYP5393A1(60431)Tremella mesenterica Fries v1.0

MNSFQQVFDRWQTNKETFSSKNAWLAAEIVLATCMVLWLYRYVQLFQQVRGLYTIHSLIE

VLEAPHRTKIPHIPVLIPTRKYTLQDPWKKYKTAKSDLIAYTQATTPGYAVYSISNPQVV

QKVLADVLSFGKPVESMGYKQLNVFGKQLVSTQSGPEHRRHRSVVKGCFSEEVMRTVWDR

SIESLDTMLSTEKVEDGGVLEHVREAMYKLTLLVIGAAGFGLKFPWSIPTTEGGSLTFHE

ALHLVEYHLVAELLVPVWILENFPLPSVRRLGNAQRSLRHHFKTMIATRRDALQAEQAGL

GPQEKIKSPSDLLGAIVASQMDIEAEEKARGGKGTSKAGLTSEEQMGNVWIFIFAGHETS

GQTLTFTLGHLAMYPEWQEKIFQEIEEVCQGSHPTYRDMNNLPSVLACVYETLRLRDLIS

LNQKLALKDTVLPYTTWDDLGQITNHTHTIKKGSHVLVDFAASGQNPFFYRDPHRWDPSR

WYGEEGNKNRSHLFNFSAGPRACIGRRFAEVEMTAILSTLIKKVKFEPVRQKGETDEQMY

DRMTQGHEVFLLTPNSFPLKFTKRQGA*

>CYP5393A2(60432)Tremella mesenterica Fries v1.0

MDMKHLRSIGSEIWAKIGQHDWTKEDAWMTVGIGFGTYIFIWFFRYLKVHYRVRGLKKIN

SAIEVFEAGHRWNLPHIPFLLPAKDFTLKEPWKKYEHAKSDLIAYTQATTPGYAVYSTSN

PQVAQKISTNVLTFGKPIHVFRYRAINIFGLQLTSTQSGPEHRRHRNVVKGCFGEEVMMT

AWDKAVECLGDMYVAEGLEDGGVLDNVRLAMYKLTLLIIGAAGFGVKFPWKVPETHGEIL

PFYEALHLVEVNITAELLVPLWLLENFPLSSVRRLGKAQRSLVHHFKNMIETRRAVLKAE

QEGLNPGEKIKPPSDLLGAIVASQMSVEAEEKLRGGSRQVGLTAEEQVATVWIFILAGHE

TSGNMLAFTLGYLSVYPEWQEKIFKEIEEACHGAYPTYREVNNMPSILACVFESLRLRDL

AMTLPKLAMEDTSIPYTTWDDHGNTTHHIHPIKKGDHIVIDGPAVGHNPFFWRDTQTWDP

TRWFGGEGVINKNLSISFSAGQRQCIGKRFAEVEMTAVLASIVKKFRFEPIRNKDETDEE

LKLRMTDGYEVLTLTPNNFSIRFIKREGV*

>CYP5394A1(18232)Tremella mesenterica Fries v1.0

MYPSGLSQIFISIAVTVTGWLFRSFDDFRGPFDPLPIIGHLHKTVRGRQFFFEWVLDGTR

YHKEKDWLLNVMGARTLLIKGPLWRAHRTAANHLFASPKTIAHIVDHVLPRNKHVLTSAL

SHAADQNVVVDAQQVMTTWLNAGFGDLAFGASSGEIHAKFSRAWTAASRPVIRRFITPFW

RMKERFQSYGLQIQKDIATARGACHDLVTRTRQAIKEEEIGIQPPRDDGSGMVIRTLLES

DYTFTEEELEDASLGFMFAGDDTTSHGLAWALYAMLRYPSYLPRLQAEIDSLPLELSSNT

ELSSHLPLLEAFISESLRLHPPTAVILSECTASQPVVMPGGAVIKPGDRIYMLPWIMARC

RSIWGEDAEVFRPERWMEGGKRKSAYEFPVFFAGPRNCPGQQLGRSEMIYTLTVVLREFE

FEAAWPMDPKDEAGKKCMEYGITSGVLGGLPVKIKRRKSGKTA*

| Species name | Protein ID | Sequence |
| --- | --- | --- |
| *Cryptococcus amylolentus* CBS 6273 | 2740583263 | MGLMDNTFIGASALVSQLITSISWSSFAQASLALLLGFYLYSYWRYRSFSFHKLPGPPADNYFLGILYSLLGSESEAPQTAWHAKYGSTLQTPFLPFPFYSSFQTTDPTALNYIVSHPDLFPKPKYIWTEIASVGGGEGLVMAEGDQHRKQRKVLNGCFTPAAIAGMVPTFYDKAYELREKLLEIVEGISDETPSLTPPQPMDQVAKGRKVDVMRYIMQATFDIIGLTGFGYQFNQLQQKGVEEDELSNVYGNLYKASMDxxxxxETLNSSPYLDAFVREVIRLSPSIPSSTRSAKEDAVIPLRHPVVGRDGKMMNRVEIPKGTDIYISIIAINTSPLYYGPDAAEFNPDRFLSPQAENDIPGVWGGSMSFFAGPRSCIGYRFALAELKTILFVLINGFEFAELPSKPMMERRRATMMRARVVGEESVGPQMPLLVKALAF |
| *Cryptococcus terricola* | 778825 | MTHLLTFFPAPPKFPFSRPSGVLPAVEYAQLRAQEPVSKVQLWDGSYAWLVVKHKDVCSV  LTDERLSKIRTRPGFPEMTAGGKAAAENKPTFVDMDPPEHMQQRSMVAPLFTREYIENMR  PHIQTTVNSLLKTMMDDGCKSPVDLVEKFALPIPSHIIYGILGTPIEDLAYLTSCNAIRT  NGSATASQASNASNELLQYLGKLVDAKTKSPGKDLISKLVMEQLEPGHLARDDVVQIAFL  MLVAGNATMVNMINLGVVTLLDHPKQLTELKKDPSLAKSFVEELCRFHTASSFATRRVAK  EDITLGGKTIKAGEGIIAATQSANRDESIFPNADTFGRHRT |
| *Cryptococcus gattii* VGIV IND107 | KIR85413.1 | MFSMMLEQLPSLQLLTTTESILLALTVFALLWLLHVFVYKPFTSPLKNVPCPPGGTGSQG  HIAEIMDLQGTKIHDWIKAYGSTFMVRGPFGVHHRIFSVDPRVLNHVLKHTNIYTKSDLL  RDLVRRYMKEGLIAAEGERHKVQRKVSQKLFSMGGLKSMGQVVQDKSNQLRDILLNLCAN  PTASNPYSPVNPTLSPGCREVDVYSTVRDVLSISLGLLALATNLIRLAIGKAQGESFSKN  MSECSFSVLAQWASGCY |
| *Naganishia vishniacii* (formerly known as *Cryptococcus vishniacii* v1.0) | CYP5697B1P (301174) | MSQDTTSRVLSWGMKYLARCPDVQKRLKEELLRHGLHERAMSFGDLQPQKMPYLEATVHEILRLATAVPGVTKTASRDCL  VLGKRIPKRATGYFPMALIATLPPTAFVADGTRWQGGPLKEFSPGRWLNDLGEFDSEAGLQSLRFSAGQRGCFGKASAML  ELKTALSKINLAFLLESISWEHHSNDFVEDLSRKPKAAHVRPVPWESIGLD* |
|  | CYP5697A1P(327266) | MDHINATIIVAGISTFAIASYFFLSILLKLVTSRPLPGIPYRDGRQYPIIGDGLDVGKWLSSQDGLMGDEFNKPTLKRKSGYKGMWQLMLGWGARSRYVVATDVSGEPHRCASLVVRVTLKLSLSSEAHRILIRRNAESDRGECVSPDLSLRTMASPTRYGSYHELKANDLVELSKRKSARLGKGQFFEAGEDFVYVTMDAISDIAFGKSFGLLPGYIDDINQSPDSHLNDQAEFPYKPNEIFYATQYLLLNLPLYSASPRLSNILQSFRLSFGHARSLVHAFVNEQIEQGRKRAILRGEENEELADCAIDIALLKDGTADELSKVELRDEAFLVIVGGVESTSQALAWGVKKLSRSPEIQGRLLNELMDAGLHEREMGYDDLVAERVPYLEATVNEVLRLAGVLGALTRTVTKDTTILGKHIPRGTNLIFPLTLMATMVTEDLAADPSCTPSLHPKWIGDSLDKFMPERWLNEDGKFDGPAPSVEYTFCSGPRSCFGRALAMLELKTTIAKIMLAFSLDKIPAEYNDNNFIEYLTRKPKTTPNRPLPRAL |
|  | CYP5705A1P(345495) | LLELPCPSPDAVVTASTRSLQQVLPSWLSDSIPPAHDYTSYPVAASGIVVATLLYLLYFRSEGPGSRGGRVPPHVASWVPWIGSGFHLATDPDRFSISRKDGSTFSITVVGKKMIYVTSPALIAQVYGNPKSFDFALIRRHMQKHIFGQKDMDEELFPANLRQLQPGTIHQLVSTFARHLSTSMVQLSGEIGVIRSYLKGPANDTHRCRKSISTMAAMMGTTFPAQEFLQKFTNFASEFTKLAEELPA*LCKGAIKHREACLDILERWYIAWNTRFDAGELQPNEQVASVTAAVLEFAHKHRTKTRDVAAFLLVDMFATQGNAPPAAAWLIIKYAENPRQLQHFPDEIDAALGKSQDGTIDGLIQAQETMNGPAFEYLTSAIKETLRYVTSVASIGRVMEETILEEKNDGVGGVLLHKGEMIYCLTRCTHIDAKVHAEPEQWVPERFMAEYRAREATKKVGKNDWLPFGGRTSMCEVGRHFALQELRTYLVAFFYHFDIQSSGPQAQLDTSRGSGSGVMPVKGSWKIAVSQRERDCRPVAP* |
| *Trichosporon asahii* var. *asahii* CBS 2479 | CYP505AE3P(5559) | MYYLLKHPETYRKAREEADKLIAEADGNMLNLNISKARYIDWVLKEALRLLPTAPGWGVE  PRNGDEVLPGGYVLKKGSGVMVMGWLLHRDKTVWGEDAYDWRPERWEHLDDLDQAAFRPF  GNGARACIGRTFAMMEGVLALIMILHRFDVEFADPGYELDIQESLTIKPRGMKVVAKPRF  SRNQSLLTELAGSTRPSKAATKTKKRANAGSGVPVNILYGSNAGTCQTFANEMAEEAAAR  GLKPFVGALDTVAGDGVLPSDGATIIFVPSYEGQPPDNAREFVQSLQRLADKPLTNASYM  VMGLGHKDWTTSFHRIPKLVDERLEALGAKRLLPLTVADVSGDATGEVEAYLETVWDVLG  VAGVEGQTTEVLDVEVLPASANAAIAEGYSFGTVLEQRTILPASESHPWTVHTVVKIPEG  QSYRVGDYLCVMPKNPEASVQRALRVSGLDANDVVKWGSLTVRAQDLFTSYVELGHTAPR  SLLPPTISYEEARAQHYTILDLMQDTHLPLSVMLASLPRMKPRHYSISSRPGDTATITYT  VHTANAGAVLGVSSNYLAHLQPGQQLQCTTKPSPGFHLPDPSVPIAMFAAGSGIAPFMAF  IAERAATGGAMTLWYGCRSAADLPYAEELVQWARSGVLDLRLSFSRSDVAEFQGLPVSKG  YVQDRVAVEKADFLRLVDGGAQFFVCGSSNRLGSGLKKTIISILDECGDGAALMDELAKK  RYKTDVFL* |
|  | CYP505AE2P(5560) | MSNVCPASASASTLEQCPVSGEQSKYASAMELPQETKPIPYPPRKFIVGNLFDLDPTRGVQCIHEIGERPEFRDIYELKI  MGQAFSIAGSYKMAKALCDETRFKKNAHRPIMLLRDMLGDGLFTALDEEESWEIAHRILLPAFGPLSLKKMQPMMVDTLT  QMLMHWEHTAGTPFDIVDQFTRLTLDTIGLCAFRFRFNSFHSEKVHPFVDNLMKVLVQIDERSKFPDGLLKLRYFANKKY  YGAIQEMFDMCDQLIAERKRNPYPDAGDLLNTMLHDKDPKTGKGLTDENIRYQVITFLSAGKCG* |
| *Trichosporon asahii* var. *asahii* CBS 8904 | CYP505AE3P(6650) | MYYLLKHPETYGKAREEADKLIAEADGNMLNLNISKARYIDWVLKEALRLLPTAPGWGVE  PRNGDEVLPGGYVLKKGSGVMVMGWLLHRDKTVWGEDAYDWRPERWEHLDDLDQAAFRPF  GNGARACIGRTFAMMEGVLALIMILHRFDVEFADPGYELDIQESLTIKPRGMKVVAKPRF  SRNQSLLTELAGSTRPSKAATKTKKRANAGSGVPVNILYGSNAGTCQTFANEMAEEAAAR  GLKPFVGALDTVAGDGVLPSDGATIIFVPSYEGQPPDNAREFVQSLQRLADKPLTNASYM  VMGLGHKDWTTSFHRIPKLVDERLEALGAKRLLPLTVADVSGDATGEVEAYLETVWDVLG  VAGVEGQTTEVLDVEVLPASANAAIAEGYSFGTVLEQRTILPASESHPWTVHTVVKIPEG  QSYRVGDYLCVMPKNPEASVQRALRVSGLDANDVVKWGSLTVRAQDLFTSYVELGHTAPR  SLLPPTISYEEARAQHYTILDLMQDTHLPLSVMLASLPRMKPRHYSISSRPGDTATITYT  VHTANAGAVLGVSSNYLAHLQPGQQLQCTTKPSPGFHLPDPSVPIAMFAAGSGIAPFMAF  IAERAATGGAMTLWYGCRSAADLPYAEELVQWARSGLLDLRLSFSRSAAAEFEGLPVAKG  YVQDRVAAEKADFLRLVDGGAQFFVCGSSNRLGSGLKKTIISILDERGDGVALMDELAKK  RYKTDVFL* |
|  | CYP505AE2P(6651) | MANVCPASASASTLEQCPVSGEQSKYASAMELPQETRPIPYPPRKFIVGNLFDLDPTRGVQCIHEIGERPEFRDIYELKI  MGQAFSIAGSYKMAKALCDETRFKKNAHRPIMLLRDMLGDGLFTALDEEESWEIAHRILLPAFGPLSLKKMQPMMVDTLT  QMLMHWEHTAGTPFDIVDQFTRLTLDTIGLCAFRFRFNSFHSEKVHPFVDNLMKVLVQIDERSKFPDGLLKLRYFANKKY  YGAIQEMFDMCDQLIAERKRNPYRDAGDLLNTMLHDKDPKTGKGLTDENIRYQVITFLSAGE* |
| *Cryptococcus neoformans* var. *neoformans* B-3501A | EAL20446.1 | MSLTLDVPPNRGIREKLEWELRDLRNFMLYLEYGIPFPQPSNSSPEPSNRSNNDNFDEDN  PYQIPSYERIASMGVFELAMYAAKYFLPKASSSPANEKAKQDLYLQIWRRDELENLVDLT  TPVHIFLGVTHCTPNAGHRYAQTRYVWSKTLEHNFHNESIMHFVLPLLSILLEDAEIRRD  DALKLCVYMEVAAPEKPRFSLPNDIARNTTKGLARLLDKKSGDSQPSAAHDRAMEAESEE  DVSSIVKRLRQSRKRILYAHSEILEEKSEYFKDLLTSGFSETQRYNTIIVDDASFSTLYW  ILHYLYTNELTFSEHEDYHYINDEHRLDNAHVDRILRPRTSTQNGLAEWNYQHLPLAGDW  DTKVDPTSLQDEPLNVKSMPSRVTNIPYPYGDKGTPQQPKRMKKDASSTSPSKIPTMAVP  LNRTFATSNNSKPHLVASHLSALGPPNNSFDAFARIGTENDPHPHPTPCPPPASALDVYM  LAHRYRLEELREMAMEQILRSLSYETCMSAAFVSYPYEELHSKVLAYIVKNWTQVKVSSA  FLRCIQEVREDVWGEYGPMVLHNIYLKI |
| *Kockovaella imperatae* NRRL Y-17943 v1.0 | CYP5393-C-term-fragment(650249) | MFIPKVVLEDSLIPYTTWDEDGNVSRHERFIRAGSHVVIDSPACSVNPFYWEDPLEFRPRRHVDERGLHIKEGFTGFSIG  QRSCIGKRFAEVESVALLSHLVKTYALKPAPVRPGETLDEMRERMVWTASEELNLTPGNFSLGFTKRT* |
|  | CYP5393-N-term-fragment(392320) | MMSQRLLVQPEAIIGTLFALVLGRWLYNYLRVRLEVLGLPTTHSLFELFESGIRSKLPQTPFVSPVASLSLQKPFEKYAN  AKTDLLAFTQATTPYAVYATANPSVAASISNKPAKYVKPVRMFRYQAINRFGRQIVSAQNGEEHKRHKGVVRACFGKEIM  ETGWTTMVDAYQVMCREEGLENGGILKDVKEVLAKVTLLVIGSASFGVQFPYNSSTSEDALMPFSDAIQLVETSMIFQLP  LPLWLMTWSPFPYLRRCAKAQRSMVKHINEMIASRRKQTTALQATLSADEQVLTQRDILGALVASQIQVEEGKKKNTAGL  TADEIFGNAFIFIVAGHTTTAHTLTFALALMALYPQTQEDIVQELASVCGDEYPTYRHMSKLPLTLATMYEALRL* |
| *Trichosporon oleaginosus* IBC0246 v1.0 | 279913 | MAPHTIRWGIISTGGIATTFAKDLLVDPTTRNVHDVAHKIAAVGSRSVASAQAFIDKLKSFSGPSSWGVQHGGLDGATPY  GSYDEVYADPSVDVVYIGTPHPFHHQNAKAALLAGKHVLCEKPFVMDLAELDELIAIAKEKRVFLMEAVWTRFQPIAYAV  QEVLRSGKLGRPRRVTAELSSCINMDELADDDRMIDPKLGGGPLLDLGPYPAVWGMLVLHQHPQNQDKEPQVVDFSQRIY  KRNGIDEMSAFTVRWEGLAEARLLTDFTACTPLDNACVVDCEKAQLIIDHPLYRPERFRIVPHAGSTDDIPKETEHRYPV  PAGNGMHYEADHVARCLRDGKLESDRMPLTESRIVQGWFDTVRKGGNSVLKDWPHTAGK* |
|  | 288219 | MAPFIASWGIIGAGWISSMFVADLALPRPEVKDVLHSVAAVGARDKARAAAFIEKHLAKGATAQQQGLRPPPVAVGGYAD  LFARKDVDIVYIGTLHPTHYEDVKGALNAGKHVLVEKPATLNAAEWQELVALATEKQLFLMEAFWTAFQPAVVALRAKLH  EEKVIGDIQAVSSNFSVPNYNVLPDDHRIIGLAAAGGPLLDIGAYGMVPARVALTDNPANKGAAPKVTCAMSKTRMGTDL  DTTLILDYANLGARAVCNLSFNARMPREHTATISGTDGEVVIHDVLCRITKFSVTKYTPGQTPQTPGKWGEPQMFEYSFP  GTGLYFEADVVARDIRDGRIENELVSHKYTSETLEIFDEARRQGGYILPQGMEKVGRVGVEDARL* |
|  | 289007 | MTFTLRWGIIATGGISTKFAEDLFIDPATRESDVAHKIAAVGSRSVASAQKFIDRLQSNPSPNQWGEVGWNAWGLEHGVL  DGAKAYGSYDEVFADANVDAVYIGTPHTFHHANAKAALLAGKHVLCEKPFTFDLEELDELIKIAKEKNLFLMEAVWTRFH  PIAYALQDLLKSGRLGKIKRMSADFSMNFEPDTRPDSNRMVDPEQGGGSLLDQGPYPSVWAMLALHQHPDNTDSDPRVVA  SYQKVYERSGVDAASTWVVSWKDFADATLVTDMTASGFNDACAVITCEEADVALAYPPWRPERFTIVPHASFEPGSIKER  ETNEFPATKAGGGMHYEADEVARCIRDGKTESERMPLAESRITQRWLDDVRKAGGTVLKDRKSTVGQ* |
