# Supplementary figures and images for: Distribution and Diversity of Cytochrome P450 Monooxygenases in the Fungal Class Tremellomycetes
Source: Int J Mol Sci. 2019 Jun 13;20(12):2889. doi: 10.3390/ijms20122889 (PMC6627453; doi:10.3390/ijms20122889)

**Taxonomy**

- CYP51
- CYP61
- CYP505
- CYP5139
- CYP5215
- CYP5216

0.1

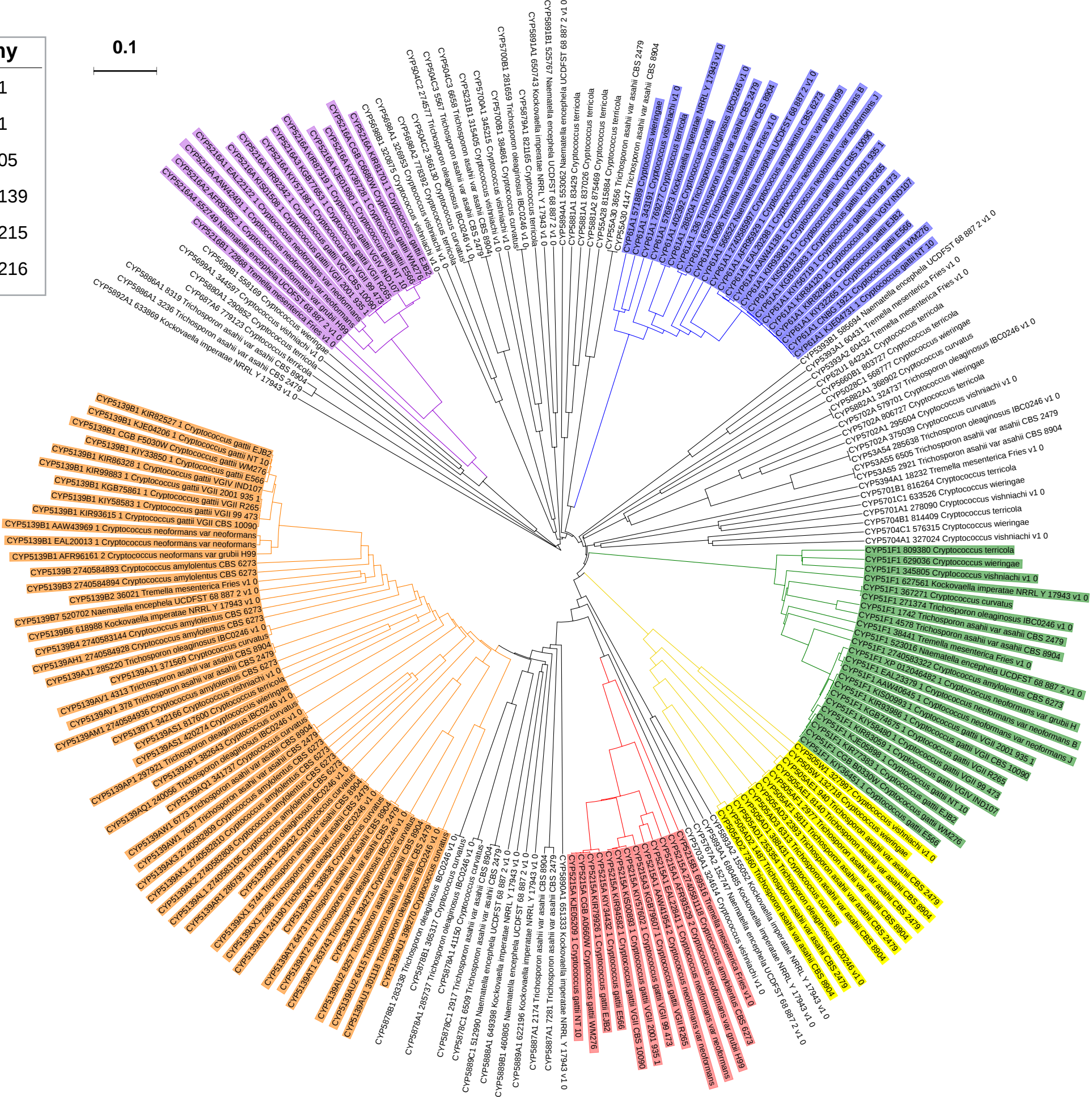

Supplement: Supplementary file 1 [file ijms-20-02889-s001.zip › Supplementary Information/Figure S1.pdf]
